# Mendelian randomisation study of body composition and depression in people of East Asian ancestry highlights potential setting-specific causality

**DOI:** 10.1186/s12916-023-02735-8

**Published:** 2023-02-01

**Authors:** Jessica O’Loughlin, Francesco Casanova, Zammy Fairhurst-Hunter, Amanda Hughes, Jack Bowden, Edward R. Watkins, Rachel M. Freathy, Iona Y. Millwood, Kuang Lin, Zhengming Chen, Liming Li, Jun Lv, China Kadoorie Biobank Collaborative Group, Robin G. Walters, Laura D. Howe, Karoline Kuchenbaecker, Jessica Tyrrell

**Affiliations:** 1 Department of Clinical and Biomedical Sciences, Faculty of Health and Life Sciences, University of Exeter, Exeter, UK; 2 Clinical Trial Service Unit and Epidemiological Studies Unit (CTSU), Nuffield Department of Population Health, University of Oxford, Oxford, UK; 3 MRC Integrative Epidemiology Unit (IEU), Bristol Medical School, Population Health Sciences, University of Bristol, Bristol, UK; 4 Department of Psychology, University of Exeter, Exeter, UK; 5 MRC Population Health Research Unit (PHRU), Nuffield Department of Population Health, University of Oxford, Oxford, UK; 6 Department of Epidemiology and Biostatistics, School of Public Health, Peking University, Beijing, China; 7 Division of Psychiatry, UCL Genetics Institute, University College London, London, UK

**Keywords:** Mendelian randomisation, BMI, Depression, East Asian ancestry, Public health, Setting-specific causality, Sociocultural factors

## Abstract

**Background:**

Extensive evidence links higher body mass index (BMI) to higher odds of depression in people of European ancestry. However, our understanding of the relationship across different settings and ancestries is limited. Here, we test the relationship between body composition and depression in people of East Asian ancestry.

**Methods:**

Multiple Mendelian randomisation (MR) methods were used to test the relationship between (a) BMI and (b) waist-hip ratio (WHR) with depression. Firstly, we performed two-sample MR using genetic summary statistics from a recent genome-wide association study (GWAS) of depression (with 15,771 cases and 178,777 controls) in people of East Asian ancestry. We selected 838 single nucleotide polymorphisms (SNPs) correlated with BMI and 263 SNPs correlated with WHR as genetic instrumental variables to estimate the causal effect of BMI and WHR on depression using the inverse-variance weighted (IVW) method. We repeated these analyses stratifying by home location status: China versus UK or USA. Secondly, we performed one-sample MR in the China Kadoorie Biobank (CKB) in 100,377 participants. This allowed us to test the relationship separately in (a) males and females and (b) urban and rural dwellers. We also examined (c) the linearity of the BMI-depression relationship.

**Results:**

Both MR analyses provided evidence that higher BMI was associated with lower odds of depression. For example, a genetically-instrumented 1-SD higher BMI in the CKB was associated with lower odds of depressive symptoms [OR: 0.77, 95% CI: 0.63, 0.95]. There was evidence of differences according to place of residence. Using the IVW method, higher BMI was associated with lower odds of depression in people of East Asian ancestry living in China but there was no evidence for an association in people of East Asian ancestry living in the USA or UK. Furthermore, higher genetic BMI was associated with differential effects in urban and rural dwellers within China.

**Conclusions:**

This study provides the first MR evidence for an inverse relationship between BMI and depression in people of East Asian ancestry. This contrasts with previous findings in European populations and therefore the public health response to obesity and depression is likely to need to differ based on sociocultural factors for example, ancestry and place of residence. This highlights the importance of setting-specific causality when using genetic causal inference approaches and data from diverse populations to test hypotheses. This is especially important when the relationship tested is not purely biological and may involve sociocultural factors.

**Supplementary Information:**

The online version contains supplementary material available at 10.1186/s12916-023-02735-8.

## Background

Obesity and depression are global health challenges and leading causes of disease burden and disability, estimated to cost the global economy billions of dollars per annum [1, 2]. Understanding the complex relationships between obesity and mental health outcomes is crucial to facilitate public health and medical intervention planning. There is extensive evidence linking higher body mass index (BMI) to higher odds of depression in adult European ancestry populations [3]. Previous work, using the genetic approach of Mendelian Randomisation (MR), by ourselves and others has provided some evidence for the causal role of higher BMI [4–8] and higher body fat percentage (BFP) [9] on depression in European populations.

However, our understanding of the relationship between obesity and depression in diverse populations is limited. The social patterning of body weight changes over the course of economic development; as a country’s wealth increases, obesity moves from being more common in higher socioeconomic groups to being associated with socioeconomic disadvantage [10]. This is likely to reflect larger body size being regarded as a positive status symbol in some cultures [11–13]. Observational studies from people of East Asian ancestry have shown both positive and negative correlations between BMI and depression [14–16], but the study designs applied have not enabled causal inference. Ideally, evidence of causal effects comes from well conducted randomised trials. However, large-scale randomised controlled trials (RCTs) cannot always be performed because they can be costly, impractical or even unethical. One of the alternatives is to perform MR experiments that are similar to RCTs in terms of study design. MR uses genetic variation as a natural experiment to investigate the causal relations between potentially modifiable risk factors and health outcomes in observational data [17]. Understanding the relationship between obesity and depression in different sociocultural contexts, using methods designed to avoid biases from confounding, such as MR, will provide important information about whether and how the relationship differs across cultural settings (‘setting-specific causality’) and therefore whether the appropriate public health response should also differ.

A recent genome-wide association study (GWAS), in people of East Asian ancestry, with 15,771 depression cases and 178,777 controls demonstrated an inverse genetic correlation between depression and body mass index (*r*_*g*_ =  − 0.212, SE = 0.084) [18]. This is contrary to findings in European populations. Here, we aim to examine this relationship in more detail by using two complementary MR approaches to test the causal role of adiposity on depression.

We use two complementary MR approaches to test the causal role of adiposity, using BMI and WHR, on depression in people of East Asian ancestry. Firstly, we perform two-sample MR [19, 20] using the inverse-variance weighted (IVW) method. Further analyses include using meta-analysed results from people of East Asian ancestry living in (a) East Asia (predominantly China) or (b) the UK or USA, allowing us to investigate the role of BMI and WHR with depression in people from the same ancestral group but residing in different cultural contexts. Secondly, we use one-sample MR approaches which draw on individual level data in the China Kadoorie Biobank (CKB). Individual level data in the CKB enables:

1. Testing effects in males and females separately, as previous studies have suggested sex-specific effects;
2. Testing further the role of cultural contexts, by comparing estimates in urban versus rural dwellers;
3. Testing non-linear relationships, as in Europeans there is some evidence that the relationship between adiposity and depression may not be linear, rather U- or J-shaped, where individuals with low BMI have higher odds of depression as well as individuals with higher BMI [21].

## Methods

### China Kadoorie Biobank (CKB)

The China Kadoorie Biobank (www.ckbiobank.org) is a study of 512,891 adults aged between 30 and 79 years at recruitment, with baseline data collected between 2004 and 2008. The CKB has been described in detail elsewhere [22], but briefly the baseline survey took place in ten geographically defined regions in China (5 urban, 5 rural). Detailed questionnaire data, physical measurements and blood samples were taken from all participants. The participants agreed to have their health followed via linkages with clinical registries and health insurance databases. Genotyping data was exported from China to the Oxford CKB International Coordinating Centre under Data Export Approvals 2014–13 and 2015–39 from the Office of Chinese Human Genetic Resource Administration. CKB analyses were conducted under project 2019–0003 as approved by the CKB Research Committee, using dataset DAR-2021–00,041 from data release 17.02.

### Exposure and outcome measures in CKB

#### A) Body mass index (BMI)

Standing height was measured to the nearest 0.1 cm using a stadiometer and weight was measured using a bioelectrical impedance device (TANITA-TBF-300GS, Tanita Corp). BMI was calculated as weight (kg) divided by the square of standing height in metres (m^2^). We also defined two binary variables to compare individuals with a normal BMI (measured BMI between 18.5 and 23.9 kg/m^2^, *n* = *51,006*), to individuals with (a) an overweight BMI (between 24 and 27.9 kg/m^2^, *n* = *33,106*) and (b) an obese BMI (greater than 28 kg/m^2^, *n* = *11,243*) as recommended by the Working Group on Obesity in China [23–25]. Participants with an underweight BMI (measured BMI less than 18.5 kg/ m^2^, *n* = *5219*) were excluded from these binary measures.

#### B) Waist hip ratio (WHR)

Waist circumference (WC; measured from midway between the lowest rib and the iliac crest) and hip circumference (HC; measured at maximum circumference around the buttocks) were measured using a soft non-stretchable tape. WHR was calculated as the ratio of WC to HC.

#### C) Depression

Several measures of depression were considered in this study. Firstly, we defined a broad depression episode. Participants were asked “During the past 12 months, have you had a period of two or more weeks in which:

1. You felt much more sad, or depressed than usual?
2. You felt worthless and useless, that everything went wrong was your fault, or that life was very difficult and there was no way out?
3. You lost interest in most things like hobbies or activities that usually give you pleasure?
4. You felt so hopeless that you had no appetite to eat even your favourite food?”

Participants responding “yes” to one or more of the above questions were included as a depressive symptoms case. A total of 17,723 participants reported that they had experienced at least one of the above Composite International Diagnostic Interview (CIDI) trigger symptoms (i.e. feeling sad/depressed, loss of appetite, loss of interest or feeling worthless) and were categorised as having depressive symptoms in our analyses. Amongst genotyped participants, we defined 3398 cases and 96,979 controls.

A major depression episode was defined using the Chinese version of the Composite International Diagnostic Interview-short form (CIDI-SF), a diagnostic instrument based on criteria from the Diagnostic and Statistical Manual of Mental Disorders-IV (DSM-IV) [26]. Major depression was defined by the presence of depressive symptoms with the addition of at least 3 of the 7 following symptoms from the CIDI-SF, including weight or appetite changes, sleeping problems, psychomotor changes, fatigue, concentration problems, feelings of guilt or worthlessness and thoughts of suicide. We defined 760 cases and 96,979 controls.

## Data analysis

### Observational associations

The association of BMI and WHR against depression was tested using logistic regression models. All models were adjusted for age at baseline, sex and region; additional models were also adjusted for socioeconomic status (SES, see Additional file 1 for details) and smoking status. We also repeated our observational analyses using the binary BMI variables.

### Two-sample Mendelian randomisation using depression GWAS summary statistics in people of East Asian Ancestry

Two-sample MR analysis was used to estimate the causal relationship between BMI and WHR with the risk of depression. Details on identification of SNPs used as genetic instruments are shown in Additional file 2: Table S1. We identified 941 SNPs for BMI [27] and 316 SNPs for WHR [28] from the GIANT (Genetic Investigation of Anthropometric Traits) and UK Biobank combined. Since some SNPs for BMI and WHR were not available in CKB, we excluded them from our analyses (*n* = 103, *n* = 53, respectively).

### Genetic variants

The genotyping and quality control of the summary statistics used have previously been described [18].

First, we extracted 838 BMI variants and 263 WHR variants (Additional file 2: Table S1) from five depression meta-analyses [18] to estimate the association with the exposure-trait-SNP. These included:

- The broad discovery analyses (15,771 cases and 178,777 controls);
- The clinical depression analyses (8223 cases and 85,370 controls), where cases were likely to fulfil DSM criteria for major depressive disorder or have a depressive disorder diagnosis in medical records;
- The symptom-based analyses (6124 cases and 73,095 controls);
- Resident in East Asia (12,027 cases and 83,727 controls);
- Resident in Western countries (UK or USA) (3744 cases and 95,050 controls).

Full details can be found in Supplementary Table 1 of Giannakopoulou et al. [18].

The instrument strength, as measured using the median *F* statistic, was 43.71 and 15.56 for BMI and WHR, respectively. Four two-sample MR methods were performed using a custom pipeline: inverse-variance weighting (IVW); MR-Egger [19]; weighted median (WM) [20]; penalised weighted median (PWM) [20]. Results from the IVW approach are presented as our main analysis method, with MR-Egger, WM and PWM representing sensitivity analyses that account for unidentified pleiotropy, which may bias our results. If the genetic variants related to the exposure of interest independently influence the outcome, then horizontal pleiotropy occurs. The IVW method assumes there is either no horizontal pleiotropy under a fixed effects model or, if using a random effects model after detecting heterogeneity amongst the causal estimates, that the strength of the association between the genetic instruments and the exposure is not correlated with the magnitude of the pleiotropic effects (the InSIDE assumption) and that the pleiotropic effects have an average value of zero. An assumption of the weighted median method is that at least 50% of the weight in the analysis stems from variants that are valid instruments. MR-Egger can provide unbiased estimates even when all SNPs violate the exclusion restriction assumption (i.e. they affect the outcome by means other than via the risk factor of interest). However, to use MR-Egger there must be negligible measurement error (NOME) in the genetic instrument and the Instrument Strength Independent of Direct Effect (InSIDE) assumption must be satisfied.

Results from MR analyses represent a valid causal effect estimate under the condition of four core assumptions:

1. The genetic instrument needs to robustly associate with the exposure (‘relevance’);
2. There should be no joint causal influence affecting the exposure instrument and the outcome (‘independence’);
3. The instrument must not affect the outcome through any mechanism other than through the exposure (‘exclusion restriction’);
4. The true relationship between the exposure and outcome in each specific analysis is correctly modelled.

An example where assumption 4 would be violated is if a linear relationship between the exposure and outcome in the model was assumed, implying a constant causal effect across all levels of the exposure, but in fact the true relationship was non-linear. We aimed to test the linearity assumption by additionally fitting quadratic causal effect models in our one-sample MR analysis.

We have reported all findings at *P* < 0.05 as tentative evidence of a relationship and used a more stringent threshold of *P* < 0.025 to account for multiple testing. We selected two groups for multiple testing based on WHR versus BMI. Other analyses are highly correlated and therefore correcting for more groups would be too stringent.

### Sensitivity analyses

We performed several two-sample MR sensitivity analyses. Firstly, we ran the analyses using 80 BMI variants identified in the Japanese GWAS of BMI (Additional file 2: Table S1) [29]. The instrument strength, as measured using the median *F* statistic, was 43.34. Secondly, we excluded SNPs where the trait raising allele in people of European ancestry was different to the trait raising allele in people of East Asian ancestry. Thirdly, we excluded any SNPs with a minor allele frequency in people of East Asian ancestry greater than 0.45 and less than 0.55 to ensure that our findings were consistent in case of a harmonisation error due to MAF close to 0.50 (*n* = 113 for BMI, *n* = 27 for WHR). Finally, we removed a variant in the *MC4R* gene (rs2229616) due to the potential strong influence of this SNP on both obesity and depression [30]. The number of SNPs remaining in these sensitivity analyses can be found in Additional file 2: Tables S2-S4.

### One-sample MR analysis in the CKB

The BMI and WHR variants that reached *P* < 1 × 10^−8^ in a European ancestry GWAS [27, 28] were extracted from the imputed CKB data. Variants were then coded based on the trait raising allele in CKB (Additional file 2: Table S1), this was assessed in each sex by regressing rank-inverse normal transformed (RINT) BMI and WHR against the corresponding set of variants in a multivariate model. When the trait increasing allele differed between males and females, the sex in which the effect size was largest was used to code the effect allele in both sexes. In a subset of unrelated CKB participants with valid genetic data (*n* = 72,698), the variants were regressed against BMI and WHR in males and females separately, with adjustment for 12 national principal components, to derive weights for genetic risk score (GRS) construction. To weight the variants in the unrelated subset of individuals, these were split into 100 randomly selected blocks, which were then iteratively removed from the multivariate regression and estimated effect sizes applied as weights to the individuals excluded from the regression. These weights were used to create GRSs in CKB. Multiallelic variants or variants with a MAF < 0.01 in CKB (*n* = 102) were excluded from GRS generation. Similarly, to the two-sample analyses, we have reported all findings at *P* < 0.05 as tentative evidence of a relationship. A more stringent threshold of *P* < 0.013 was used to account for multiple testing. We selected four groups for multiple testing based on WHR versus BMI and rural versus urban. All of our analyses are highly correlated and therefore correcting for more groups would be too stringent.

### Genetic variants

Genotyping was conducted using a custom made 800 K single nucleotide variation array (Axiom, Affymetrix) with imputation to 1000 Genomes Phase 3. Genotype data were available for 100,574 individuals whose samples passed quality control (call rate > 99.97% across all variants).

We employed the two-stage least-squares regression estimator [31] method which uses predicted levels of BMI/WHR per genotype and regresses the depression outcome against these predicted values. First, we calculated the association between the BMI or WHR GRS and BMI or WHR, respectively. These predicted values were then used as the independent variable and depression as the dependent variable in a logistic regression model. The analyses were performed by region using region-specific principal components and stratified by sex. The resulting estimates and standard errors were then meta-analysed using a fixed effects model to provide estimates for urban and rural dwellers and all individuals. The heterogeneity of the estimates was assessed using the *I*^2^ statistic.

### Sensitivity analyses

We repeated our analyses using a more clinically relevant measure of depression (major depression) defined using the CIDI-SF (760 cases and 96,979 controls). Further, we meta-analysed the region-specific estimates excluding regions with less than 1% prevalence of depressive symptoms.

### Non-linear Mendelian randomisation

Here, we used advances in MR methodology [32] to test the non-linear relationship between BMI and depressive symptoms in the CKB. We implemented a control function (CF) method. In the first stage, we regressed the instrumental variable (BMI GRS) on the exposure (BMI) whilst accounting for covariates in sex and region stratified analyses. In the second stage, the outcome (depressive symptoms) was regressed on the exposure (BMI), covariates and the residual of the first stage regression. The coefficients of the second stage were taken as the control function estimates. The residual of the first stage was used to account for unmeasured confounders. The control function method fits a quadratic model to the data and allows us to detect evidence of non-linearity between our exposure and outcome.

## Results

The demographics of the 3398 individuals in the CKB with depressive symptoms and the 96,979 controls with valid genetic data are summarised in Table 1. Generally, individuals affected by depressive symptoms were younger, more likely to be female and to live in rural areas of China.

**Table 1:** The demographics and lifestyle characteristics of individuals from the China Kadoorie Biobank with valid genetic data available

Demographic	Depressed	Not depressed	*P*^a^
*N*	3398	96,979	
Mean age at recruitment (SD)	52.6 (10.6)	53.8 (11.0)	6.00E − 10
Female, *N* (%)	2228 (65.6)	55,215 (56.9)	< 1.00E − 15
Lives in urban region, *N* (%)	1080 (31.8)	42,800 (44.1)	< 1.00E − 15
Region, *N* (%)			< 1.00E − 15
*Qingdao (Urban)*	58 (1.7)	8201 (8.5)	
*Harbin (Urban)*	460 (13.5)	12,637 (13.0)	
*Haikou (Urban)*	103 (3.0)	5679 (5.9)	
*Suzhou (Urban)*	298 (8.8)	7660 (7.9)	
*Liuzhou (Urban)*	161 (4.7)	8623 (8.9)	
*Sichuan (Rural)*	344 (10.1)	10,.248 (10.6)	
*Gansu (Rural)*	371 (10.9)	9651 (10.0)	
*Henan (Rural)*	573 (16.9)	10,782 (11.1)	
*Zhejiang (Rural)*	575 (16.9)	11,452 (11.8)	
*Hunan (Rural)*	455 (13.4)	12,046 (12.4)	
Mean BMI (SD)	23.1 (3.5)	23.7 (3.5)	< 1.00E − 15
Mean WHR (SD)	0.87 (0.1)	0.88 (0.1)	5.60E − 15
Mean BFP (SD)	27.7 (8.7)	27.8 (8.7)	0.96
Sleep hours	7.00 (1.7)	7.39 (1.4)	< 1.00E − 15
Mean MET hours (physical activity metric) (SD)	6.25 (3.7)	6.02 (3.4)	2.00E − 04
Smoking status, *N* (%)			1.30E − 07
*Never smoker*	2191 (64.5)	57,995 (59.8)	
*Occasional smoker*	196 (5.8)	5460 (5.6)	
*Former smoker*	167 (4.9)	6742 (7.0)	
*Current smoker*	844 (24.8)	26,782 (27.6)	
Highest education, *N* (%)			< 1.00E − 15
*No formal school*	836 (24.6)	19,260 (19.9)	
*Primary school*	1173 (34.5)	32,047 (33.1)	
*Middle school*	877 (25.8)	26,001 (26.8)	
*High school*	384 (11.3)	13,827 (14.3)	
*Technical school/college*	88 (2.6)	3414 (3.5)	
*University*	40 (1.2)	2430 (2.5)	
Household income, *N* (%)			< 1.00E − 15
< *2500 yuan*	243 (7.2)	3670 (3.8)	
*2500–4999*	403 (11.9)	7163 (7.4)	
*5000–9999*	824 (24.3)	18,813 (19.4)	
*10,000–19,999*	962 (28.3)	29,210 (30.1)	
*20,000–34,999*	665 (19.6)	22,479 (23.2)	
> = *35,000*	301 (8.9)	15,644 (16.1)	
Occupation, *N* (%)			0.02
*Agriculture*	1615 (47.5)	39,480 (40.7)	
*Factory*	276 (8.1)	11,448 (11.8)	
*Administrative/manager*	40 (1.2)	2286 (2.4)	
*Professional/technical*	79 (2.3)	2740 (2.8)	
*Sales and service*	160 (4.7)	4543 (4.7)	
*Retired*	383 (11.3)	18,883 (19.5)	
*House husband/wife*	549 (16.2)	11,027 (11.4)	
*Self employed*	64 (1.9)	2402 (2.5)	
*Unemployed*	156 (4.6)	2740 (2.8)	
*Other*	76 (2.2)	1430 (1.5)	
Marital status, *N* (%)			< 1.00E − 15
*Married*	2676 (78.8)	86,855 (89.6)	
*Widowed*	558 (16.4)	7998 (8.3)	
*Divorced*	120 (3.5)	1383 (1.4)	
*Never married*	44 (1.3)	743 (0.8)	
Has health cover *N* (%yes)	2423 (71.3)	76,532 (78.9)	< 1.00E − 15
Has own home *N* (%yes)	1374 (40.4)	41,450 (42.7)	7.60E − 03
Has private toilet *N* (%yes)	1467 (43.2)	49,196 (50.7)	< 1.00E − 15
Has motor vehicle *N* (%yes)	1586 (46.7)	46,387 (47.8)	0.18
Has own phone *N* (%yes)	2832 (83.3)	84,915 (87.6)	4.10E − 13
Recent holiday *N* (%yes)	212 (6.2)	9500 (9.8)	8.70E − 12
Satisfaction level, *N* (%)			< 1.00E − 15
*Very satisfied*	348 (10.2)	17,207 (17.7)	
*Satisfied*	1145 (33.7)	49,521 (51.1)	
*Neither satisfied or unsatisfied*	1192 (35.1)	26,600 (27.4)	
*Unsatisfied*	624 (18.4)	3449 (3.6)	
*Very unsatisfied*	89 (2.6)	202 (0.2)	
Self-rated health *N* (%)			< 1.00E − 15
*Excellent*	230 (6.8)	16,942 (17.5)	
*Good*	624 (18.4)	26,525 (27.4)	
*Fair*	1512 (44.5)	42,986 (44.3)	
*Poor*	1032 (30.4)	10,526 (10.9)	
Comparative health *N* (%)			< 1.00E − 15
*Better*	374 (11.0)	17,870 (18.4)	
*About the same*	1619 (47.7)	60,289 (62.2)	
*Worse*	1320 (38.9)	16,101 (16.6)	
*Do not know*	85 (2.5)	2719 (2.8)	
Loss of interest *N* (%)	1483 (43.6)	0	
Feeling worthless *N* (%)	1021 (30.1)	0	
Loss of appetite *N* (%)	860 (25.3)	0	
Continuous anxiety *N* (%)	485 (2913)	0	
Sad depressed *N* (%)	3233 (95.1)	0	
Major depression *N* (%)	760 (100)	0	

*P*^a^ Comparison of cases and controls using linear (for continuous) and logistic (for binary) regression models

Within the CKB, the BMI GRS was robustly associated with BMI, explaining 1.5% (*F* = 637.87) and 3.1% (*F* = 1815.94) of the variance in males and females, respectively. Similarly, the WHR GRS was robustly associated with WHR explaining 0.4% (*F* = 191.74) of the variance in males and 0.9% (*F* = 516.41) of the variance in females. Within each region the BMI GRS was robustly associated with BMI (Additional file 2: Table S5), but the WHR GRS was not as robustly associated across the different regions. For example, in males from Qingdao, a unit higher BMI GRS was robustly associated with higher BMI [beta: 0.57, 95% confidence intervals (95% CI): 0.45, 0.70], but the WHR GRS was not as robustly associated with higher WHR [beta: 0.25, 95% CI: 0.03, 0.47].

### Observationally, higher adiposity was associated with lower odds of depressive symptoms in the CKB

**Fig. 1:**
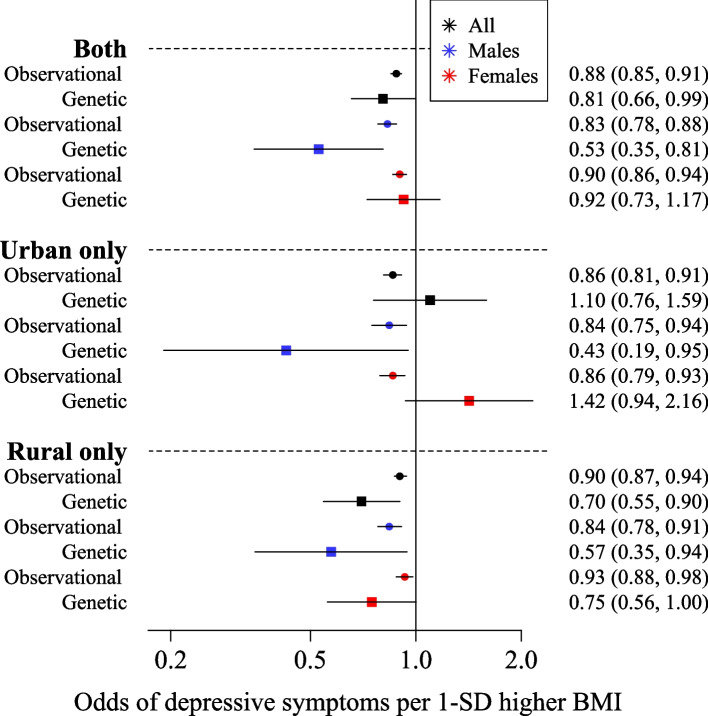
Forest plot of the observational and genetic associations between a 1-SD higher BMI and the odds of depressive symptoms. The plots display the observational association and the genetic association using the two-step instrumental variable analysis with the BMI genetic risk score (genetic one-sample)

Observationally, higher BMI was associated with lower odds of depressive symptoms (Table 2). For example, a one-standard deviation (1-SD) (3.50 kg/m^2^) higher BMI was associated with 0.88 [95% CI: 0.85, 0.91] lower odds of depressive symptoms in all individuals (Table 2, Fig. 1). The observational associations were consistent in males and females and when stratified by urban and rural regions (Table 2, Fig. 1). Adjusting the observational analyses for SES and smoking status slightly attenuated the effect estimates towards the null, but higher adiposity remained inversely associated with depressive symptoms (Additional file 2: Table S6).

**Table 2:** The observational and genetic one-sample associations between BMI and WHR with depressive symptoms in individuals with valid genetic data from the China Kadoorie Biobank

				**Observational**		**Genetic**			
**Instrument used**	**Strata**	**Region**	***N* cases (controls)**	**OR (95% CI) per 1-SD higher adiposity measure**	***P***^**a**^	**OR (95% CI) per 1-SD higher adiposity measure**	***P***^**b**^	***P***_***sex***_	***P***_***region***_
BMI	All	Both	3398 (96,979)	0.88 (0.85, 0.91)	1.50E − 12	0.81 (0.66, 0.99)	0.042		
	Male	Both	1170 (41,764)	0.83 (0.78, 0.88)	2.00E − 09	0.53 (0.35, 0.81)	***3.00E − 03***	**0.024**	
	Female	Both	2228 (55,215)	0.90 (0.86, 0.94)	1.80E − 06	0.92 (0.73, 1.17)	0.508		
	All	Urban only	1080 (42,800)	0.86 (0.81, 0.91)	2.20E − 06	1.10 (0.76, 1.59)	0.621		**0.049**
	Male	Urban only	326 (17,639)	0.84 (0.75, 0.94)	2.70E − 03	0.43 (0.19, 0.95)	**0.037**	**9.24E − 03**	0.542
	Female	Urban only	754 (25,161)	0.86 (0.79, 0.93)	7.90E − 05	1.42 (0.94, 2.16)	0.100		**0.014**
	All	Rural only	2318 (54,179)	0.90 (0.87, 0.94)	3.70E − 06	0.70 (0.55, 0.90)	***5.00E − 03***		
	Male	Rural only	844 (24,125)	0.84 (0.78, 0.91)	8.00E − 06	0.57 (0.35, 0.94)	**0.029**	0.363	
	Female	Rural only	1474 (30,054)	0.93 (0.88, 0.98)	6.90E − 03	0.75 (0.56, 1.00)	0.051		
WHR	All	Both	3398 (96,979)	0.91 (0.88, 0.94)	4.30E − 07	0.74 (0.55, 1.00)	0.049		
	Male	Both	1170 (41,764)	0.88 (0.83, 0.94)	3.90E − 05	0.66 (0.37, 1.18)	0.162	0.668	
	Female	Both	2228 (55,215)	0.92 (0.88, 0.96)	1.10E − 04	0.77 (0.54, 1.10)	0.148		
	All	Urban only	1080 (42,800)	0.87 (0.81, 0.93)	2.00E − 05	0.82 (0.48, 1.40)	0.460		0.656
	Male	Urban only	326 (17,639)	0.85 (0.75, 0.95)	4.40E − 03	0.45 (0.17, 1.16)	0.096	0.132	0.308
	Female	Urban only	754 (25,161)	0.86 (0.80, 0.93)	1.50E − 04	1.09 (0.57, 2.08)	0.806		0.216
	All	Rural only	2318 (54,179)	0.93 (0.89, 0.97)	1.70E − 03	0.71 (0.49, 1.02)	0.061		
	Male	Rural only	844 (24,125)	0.89 (0.84, 0.96)	1.50E − 03	0.83 (0.40, 1.72)	0.624	0.597	
	Female	Rural only	1474 (30,054)	0.95 (0.90, 1.00)	0.053	0.67 (0.44, 1.02)	0.059		

*P*^a^ adjusted for age, region, and sex

*P*^b^ adjusted for age, regional principal components, and chip

*P*_sex_ comparison of male and female one-sample estimates using Fisher’s *z*-score method

*P*_region_ comparison of urban and rural one-sample estimates using Fisher’s *z*-score method

**Bold** *P* values represent a significant (*P* < 0.05) difference when comparing the by sex or by region estimates

**Bold** *P* values reach *P* < 0.05 significance and **bold italicised** *P* values reach the multiple testing threshold *P* < 0.013

Using the binary obesity measure as the predictor in these models did not alter our findings. Observationally, overweight, and obese individuals were at 0.87 [95% CI: 0.80, 0.94] and 0.84 [95% CI: 0.74, 0.95] lower odds of depressive symptoms respectively, than those of normal BMI (Additional file 2: Table S7).

Similar results were seen when WHR was considered, with a 1-SD higher WHR (an increase of 0.07) associated with 0.91 [95% CI: 0.88, 0.94] lower odds of depressive symptoms in all individuals (Table 2, Additional file 3: Fig. S1).

### MR analyses provide evidence that higher BMI causes lower depression in people of East Asian ancestry

#### Two-sample MR in people of East Asian ancestry using GWAS summary statistics

**Fig. 2:**
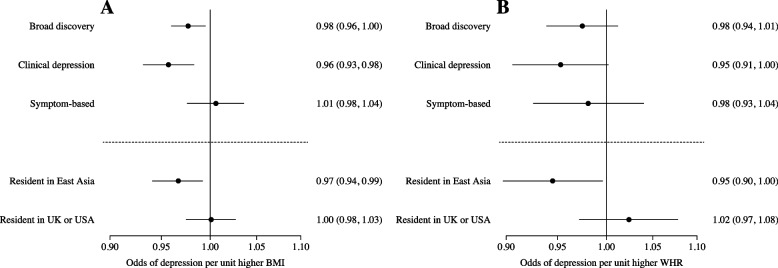
Results of the two-sample Mendelian randomisation analysis for the five meta-analyses from the recent GWAS in people of East Asian ancestry using the inverse-variant weighted method for **A** BMI and **B** WHR

Two-sample MR provided evidence that higher genetically instrumented BMI was associated with lower odds of depression. The results were consistent for the broad discovery outcome and the clinical depression outcome, but not with the symptom-based measure of depression where the effect estimate was on the null (Fig. 2, Additional file 2: Table S8). For example, using the clinical depression definition, a unit higher genetically instrumented BMI was associated with and odds ratio for depression of 0.96 [95% CI: 0.93, 0.98].

Genetically instrumented WHR was associated with lower odds of both the broad and clinical depression outcomes, but the confidence intervals crossed the null (Fig. 2, Additional file 2: Table S8).

Results using more pleiotropy resistant methods were directionally consistent, but all confidence intervals crossed the null (Additional file 2: Table S9). There was no strong evidence of horizontal pleiotropy with MR-Egger (Additional file 2: Table S9).

#### One-sample MR in the China Kadoorie Biobank

One-sample MR in 100,377 people of East Asian ancestry provided evidence for a causal role of higher adiposity in depressive symptoms (Table 2). In all individuals, a genetically-instrumented 1-SD higher BMI was associated with 0.81 [95% CI: 0.66, 0.99] lower odds of depressive symptoms (Table 2, Fig. 2).

One-sample MR methods also provided casual evidence that higher WHR was associated with lower odds of depressive symptoms. For example, a genetically-determined 1-SD higher WHR was associated with 0.74 [95% CI: 0.55, 1.00] lower odds of depressive symptoms in all individuals (Table 2, Additional file 3: Fig. S1).

#### Differential effects were observed when stratifying by international place of residence

Using the summary statistics from the five meta-analyses of depression, we were able to compare the relationship of higher BMI with depression in people of East Asian ancestry living in East Asia (predominantly China) with those of the same ancestry but dwelling in either the UK or the USA. Here, as with the main analyses, a genetically-instrumented unit higher BMI was associated with 0.97 [95% CI: 0.94, 0.99] lower odds of depression in individuals living in East Asia. However, this was attenuated to the null in people of East Asian ancestry living in the UK and USA (Fig. 2, Additional file 2: Table S8).

The results were similar when instrumenting WHR, with higher genetic WHR associated with lower odds of depression in people of East Asian ancestry living in East Asia, but not in those who live in the UK or USA (Fig. 2, Additional file 2: Table S8).

#### Differential effects were observed when stratifying by sex and place of residence within China

We were able to test whether sex and place of residence altered the relationship between higher BMI and depressive symptoms within China using data from the CKB.

Observationally, associations were consistent in males and females and when stratified by urban and rural regions, with higher BMI and higher WHR consistently associating with lower odds of depressive symptoms (Fig. 1, Table 2 and Additional file 2: Table S6). Classifying individuals as having a normal or obese BMI provided consistent results, with males and females with obesity at lower odds of depressive symptoms (OR:0.74, 95% CI:0.58, 0.95 and OR:0.86, 95% CI:0.75, 1.00 respectively; Additional file 2: Table S7).

One-sample MR methods demonstrated that the inverse relationship between BMI and depressive symptoms was stronger in males than females (*P*_*sex*_ = 0.024; Table 2, Fig. 1). Here, a genetically-instrumented 1-SD higher BMI was associated with 0.53 [95% CI: 0.35, 0.81] lower odds of depressive symptoms in males. In contrast, in females, there was no conclusive evidence of an inverse relationship between BMI and depressive symptoms (OR: 0.92, 95% CI: 0.73, 1.17; Table 2).

When comparing all individuals from urban and rural regions, genetic analyses suggested that higher BMI was associated with lower odds of depressive symptoms in rural regions only (Table 2, Fig. 1), with the overall estimate for urban regions in the positive direction, although the confidence intervals crossed the null (OR: 1.10 95%CI: 0.76, 1.59; *P*_*region*_ = 0.049).

In urban regions, strong sex-specific effects were observed, with higher genetic BMI associated with lower odds of depressive symptoms in males only (OR: 0.43 [95%CI: 0.19, 0.95]; Table 2, Fig. 1). In contrast, one-sample MR estimates from urban females were in the opposite direction (*P*_*sex*_ = 0.009), with higher BMI associating with higher odds of depressive symptoms, although confidence intervals crossed the null (OR: 1.42, 95% CI: 0.94, 2.16).

Further, there was evidence that higher BMI was causally associated with lower odds of depressive symptoms in all individuals from rural regions. A genetically-determined 1-SD higher BMI was associated with 0.70 [95% CI: 0.55, 0.90] lower odds of depressive symptoms (Table 2, Fig. 1). Estimates were consistent in both males (OR: 0.57, 95% CI: 0.35, 0.94) and females (OR: 0.75, 95% CI: 0.56, 1.00) with higher BMI associating with lower odds of depressive symptoms.

Analyses were performed within each of the 10 regions in the CKB, with higher heterogeneity observed between the estimates in rural regions, especially for the female only analyses, although the *P*_*heterogeneity*_ > 0.05 in all cases (*I*^2^ = 49.3%; Additional file 2: Table S10). At the region level, there was tentative evidence of an inverse relationship between BMI and depressive symptoms in males from Suzhou (*P* = 0.03) and females from Henan (*P* = 0.004, Additional file 3: Fig. S2), whilst with the major depression outcome, we observed an inverse relationship with BMI in females from Henan (*P* = 0.03) and males from Zhejiang (*P* = 0.03, Additional file 3: Fig. S3).

Findings for WHR were less conclusive with directionally consistent results observed in males and females but wide confidence intervals which crossed the null limiting the interpretability of our findings (Table 2, Additional file 3: Fig. S1). Similarly, there was no conclusive evidence for a relationship between WHR and depressive symptoms in individuals from urban or rural areas (Table 2, Additional file 3: Fig. S1).

### Secondary analyses

#### Two-sample MR analyses

We performed several two-sample MR sensitivity analyses and observed the following results:

1. Repeating the BMI two-sample MR analyses using 80 BMI genetic variants identified in a Japanese population. Here, our findings were directionally consistent, with higher BMI associating with lower odds of depression, although the confidence intervals crossed the null (Additional file 2: Table S11).
2. Excluding SNPs where the minor allele frequency was close to 0.50 (> 0.45 and < 0.55) provided consistent results when compared to using all SNPs (Additional file 2: Table S2; Additional file 3: Figs S4-S13).
3. Excluding SNPs with a different trait raising allele between people of European and East Asian ancestry were consistent, although confidence intervals were wider (Additional file 2: Table S3; Additional file 3: Figs S4-S13).
4. Removing the *MC4R* SNP which appeared to be an outlier and is in an important BMI-related gene in people of European ancestry also did not alter our results (Additional file 2: Table S4).

#### One-sample MR analyses

**Fig. 3:**
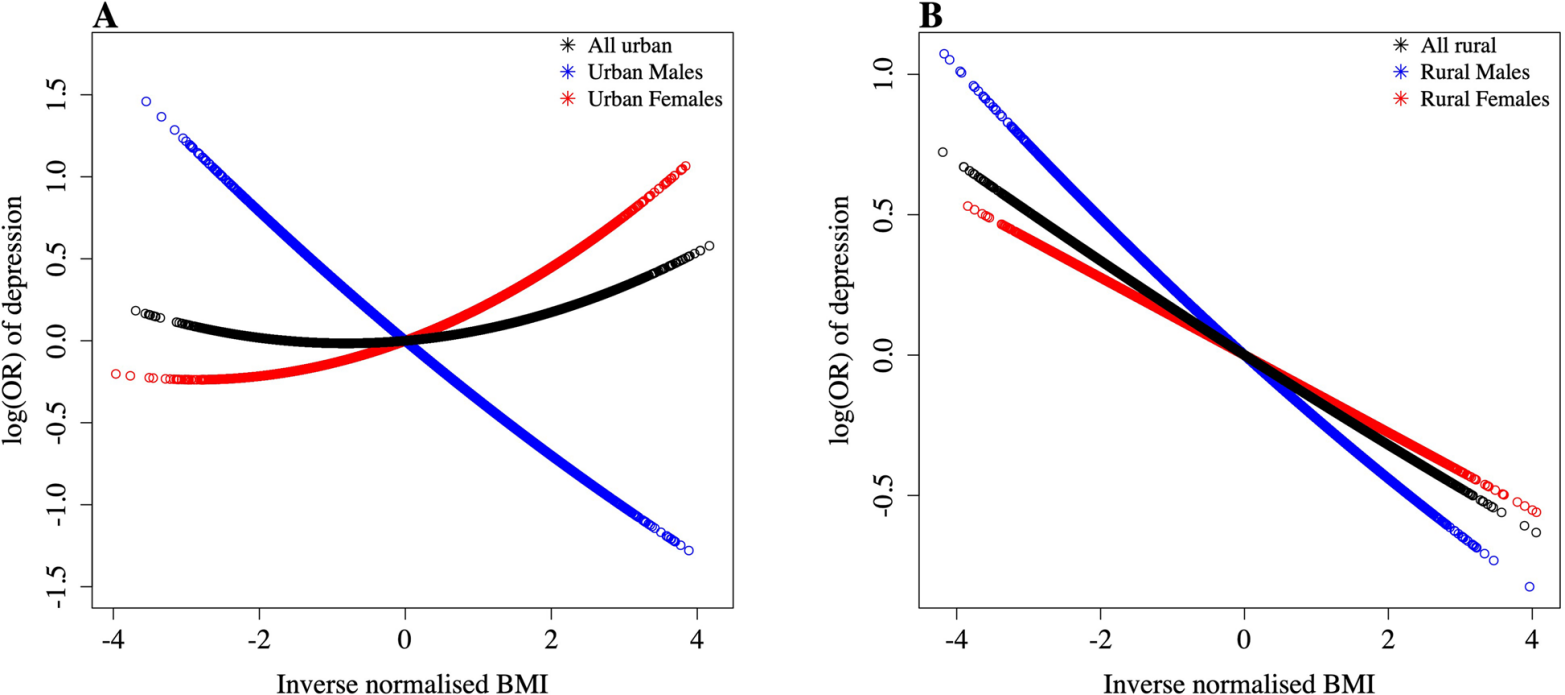
The fitted NLMR regression estimates using a CF approach in **A** urban regions and **B** rural regions stratified by sex

We performed several secondary one-sample MR analyses in the CKB and observed the following results:

1. We tested for evidence of non-linearity between BMI and depressive symptoms in the CKB, using a CF approach. This provided some evidence for a non-linear relationship in urban dwelling individuals, especially females, suggesting both high and low BMI is associated with higher odds of depressive symptoms (Fig. 3a, Additional file 2: Table S12). There was no evidence of a non-linear relationship in individuals from rural areas (Fig. 3b, Additional file 2: Table S12).
2. Analyses were repeated with a more stringent measure of depression: major depression defined using the CIDI-SF. Although broader confidence intervals were observed, both the observational and genetic one-sample results were directionality consistent with the main analyses (Additional file 2: Table S13).
3. Finally, we repeated the genetic one-sample analyses excluding Qingdao as the prevalence of depressive symptoms in this region was < 1% in both males (0.55%) and females (0.81%). The results were consistent when Qingdao was excluded (Additional file 2: Table S14).

## Discussion

This study tested the causal role of higher adiposity (as indexed by BMI or WHR) on depression outcomes in people of East Asian ancestry. Using two-sample Mendelian randomisation methods and summary statistics from a recent GWAS of major depressive disorder in people of East Asian ancestry with 15,771 cases and 178,777 controls, as well as one-sample methods in 100,377 individuals in the China Kadoorie Biobank, we provide evidence that higher adiposity reduces the risk of depression in people of East Asian ancestry. We also provide evidence that this relationship varies depending on place of residence both internationally and within China. The GWAS data provided evidence that higher adiposity associated with reduced risk of depression in people of East Asian ancestry living in East Asia. However, an identical analysis in people of East Asian ancestry dwelling in the UK or USA provided no conclusive evidence of an association. Further, within China, different effect estimates were observed between people dwelling in urban versus rural areas, with strongly consistent inverse associations between BMI and depressive symptoms in rural regions, but an overall trend in the opposite direction in urban regions, driven by females.

This study contrasts to findings within European populations where higher adiposity is associated with higher odds of depression and depressive symptoms [6–8]. The findings of this study fit with our previous hypothesis in Europeans that the relationship between adiposity and depression could be explained by both biological (e.g. metabolic or inflammatory) and/or social factors (e.g. weight related stigma). We previously demonstrated within European populations, using genetic variants that increase adiposity but are also associated with a more favourable metabolic profile, that it was not solely the adverse metabolic consequences of higher adiposity that were driving the relationship with depression [33]. The perceptions of obesity in different cultures may therefore contribute to the relationship between obesity and depression. Our results in this study would fit with previous literature in East Asian populations [15, 34] where a ‘Jolly Fat’ hypothesis is considered [35]. Historically, Chinese tradition dictates that obesity is perceived positively, with the idiom ‘happy mind and fat body’, because only the wealthy can afford the food to gain weight [36, 37]. This is very different to the perception of obesity in European populations, where there is evidence that weight-related stigma has a role in driving psychological distress [38–41]. These different perceptions of obesity across different cultures may explain the differential effects observed here.

There is further evidence for the importance of sociocultural factors within our study. Firstly, within the CKB there was evidence of differential effect estimates between urban and rural dwellers, with strong inverse relationships in rural dwellers, but within urban regions the direction of effect was in the opposite direction. The urban estimates were driven by females, where higher BMI was trending towards higher risk of depressive symptoms. Furthermore, urban females provided the strongest evidence of a non-linear relationship between BMI and depressive symptoms, with both high and low BMI associating with higher odds of depressive symptoms. This fits with (a) our understanding of the social patterning of obesity as populations develop economically and (b) the transformation in perceptions within the Chinese populations, as idealisation of a thin body type is becoming more common, particularly in Chinese females [10, 42].

Secondly, people of East Asian ancestry living in Western countries (UK and USA) demonstrated no clear association between BMI and depression, falling somewhere between the findings observed in people of European ancestry, where higher BMI is associated with higher risk of depression, and the inverse findings observed here in people of East Asian ancestry living in China. There is evidence of changes in cultural identity over time in individuals who have migrated, with a transition towards the views of the country of migration [43]. This may help to explain the differences observed here, with the perception of obesity varying between cultures the UK or USA compared to those in China. However, we acknowledge that our UK/USA dwelling analyses had lower power and potential heterogeneity in the definitions of depression in these studies may have contributed to the null findings. Therefore, future work needs to analyse these differences in larger numbers of individuals with consistent depression definitions.

We note that one-sample MR estimates for urban dwelling females were in the opposite direction to the observational estimates obtained within CKB. This may be a result of (a) unmeasured or residual confounding within our observational analyses [44] or (b) reverse causation [45]. Given that it is the estimates for urban females which switch direction and this fits with the social patterning of obesity, it is feasible that the wealth of the area or an individual’s wealth may confound the association. Accurately accounting for this in our observational analyses was not possible, although adjustment for socioeconomic factors did attenuate the observational estimates towards the null, tending to support the above hypothesis. It is also feasible that, as one of the symptoms of depression is weight change, we are seeing reverse causation in our observational models which cannot be accurately accounted for. Here, we cannot exclude reverse causal pathways as we were unable to test the role of depression on adiposity using genetic approaches.

This study benefits from two complementary datasets, including a two-sample MR analysis in 15,771 cases and 178,777 controls of people of East Asian ancestry and a one-sample MR analysis in individual level data in up to 100,377 individuals in the CKB. The individual level data in the CKB enabled sex-specific analyses, non-linear analyses, and further regional analyses, which allowed us to comprehensively test the influence of higher adiposity on depressive symptoms. We acknowledge some limitations with our approaches, especially in the one-sample analyses. In both the one- and two-sample approaches the definition of depression in the largest sample sizes captures depressive symptoms, however, our findings were directionally consistent when using the more clinically representative measure in both analyses. The CKB has a low prevalence of reported depressive symptoms (3.5%), especially when analysing certain regions, and therefore the power of our sex-specific regional analyses is limited. Further, after considering Bonferroni corrected significance (*P* < 0.01), our one-sample results of BMI to depressive symptoms in some of our sub-analyses remained only nominally significant (*P* < 0.05). This is likely due to low numbers of depression cases in some regions. In the CKB analyses, we were unable to account for potential pleiotropy in our models; however, the more pleiotropy-robust methods used in the two-sample analyses were directionally consistent. Whilst we considered all four key assumptions of MR, we were only able to use available data to test the exclusion restriction assumption and it is possible that the BMI GRS may associate with unmeasured confounders. Additionally, genetic associations can be confounded by demographic and family-level processes [46], for example indirect effects of parents’ genotype on offspring phenotype via environmental pathways which may violate the independence assumption specifically. Circumventing these sources of bias requires genotype data on multiple members of the same family, which was not available here. Furthermore, we lacked robust BMI variants in people of East Asian ancestry and therefore utilised variants identified in European populations. However, these were weighted by and aligned to the trait raising allele in the CKB, meaning that the BMI GRS was strongly associated with BMI in the CKB (*F*-stat > 100). Future work should repeat these analyses using specific variants identified in people of East Asian ancestry. Finally, we did not have full summary statistics available for the East Asian major depression GWAS and therefore we were unable to rule out reverse causation (depression influencing BMI) which should be tested in future work.

## Conclusions

In conclusion, we have used two complementary Mendelian randomisation methods to test the causal role of adiposity on depression and depressive symptoms in people of East Asian ancestry, demonstrating that higher adiposity is associated with lower odds of depression and depressive symptoms. Our analyses suggest that sociocultural contexts are important in this relationship, with no association noted in people of East Asian ancestry living in the UK and USA. This study highlights the importance of considering setting-specific causal relationships especially when the relationship tested may have non-biological pathways.

## Supplementary Information

**Additional file 1:** Supplementary methods.

**Additional file 2:** **Supplementary tables S1-S14. Table S1. **SNPs used in the 2-sample MR. **Tables S2-S4.** 2-sampleMendelian Randomisation sensitivity analyses. **Table S5.** The association betweenthe BMI/WHR GRS and BMI/WHR. **Table S6.** The observational analyses furtheradjusted for measures of socioeconomic status. **Table S7.** The observationalassociations between higher BMI and depression classifying individuals ashaving a normal, overweight or obese BMI. **Table S8.** 2-sample MR results usingthe IVW method. **Table S9.** 2-sample MR results using MR-Egger, WM and PWM methods.**Table S10.** The meta-analysed estimates with heterogeneity statistics for1-sample MR analyses. **Table S11.** Results of the 2-sample MR analysis using theSNPS from the BioBank Japan. **Table S12.** The meta-analysed non-linear estimatesusing a control function method. **Table S13.** The observational and 1-sample MRresults of adiposity and major depression. **Table S14.** The 1-sample MR results removingQingdao with a <1% prevalence of depressive symptoms.

**Additional file 3:** **Fig. S1.** Observational and genetic associationsbetween WHR and depressive symptoms. **Fig. S2.** The genetic 1-sample MR estimatesof BMI to depressive symptoms stratified by sex and region. **Fig. S3.** Thegenetic 1-sample MR estimates of BMI to major depression stratified by sex andregion. **Figs. S4-S8.** Scatter plots of SNP-depression outcome vs. SNP-BMI2-sample MR analyses. **Figs. S9-S13.** Scatter plots of SNP-depression outcome vs.SNP-WHR 2-sample MR analyses.

## Data Availability

The data that support the findings of this study are available from the China Kadoorie Biobank Collaborative Group, but restrictions apply to the availability of these data, which were used under licence for the current study, and so are not publicly available. Summary data are however available from the authors upon reasonable request and with permission of the China Kadoorie Biobank Collaborative Group.

## Abbreviations

BFP: Body fat percentage
BMI: Body mass index
CF: Control function
CI: Confidence interval
CIDI: Composite International Diagnostic Interview
CKB: China Kadoorie Biobank
DNA: Deoxyribonucleic acid
DSM: Diagnostic and Statistical Manual of Mental Disorders
GIANT: Genetic Investigation of Anthropometric Traits
GRS: Genetic risk score
GWAS: Genome-wide association study
HC: Hip circumference
IVW: Inverse-variance weighted
MAF: Minor allele frequency
MR: Mendelian randomisation
NLMR: Non-linear Mendelian randomisation
NOME: Negligible measurement error
OR: Odds ratio
PC: Principal component
PWM: Penalised weighted median
SD: Standard deviation
SE: Standard error
SES: Socioeconomic status
SNP: Single-nucleotide polymorphism
WC: Waist circumference
WHR: Waist-hip ratio
WM: Weighted median

## References

[1] Tremmel M, Gerdtham UG, Nilsson PM, Saha S: Economic burden of obesity: a systematic literature review. *Int J Environ Res Public Health* 2017, 14(4). 10.3390/ijerph14040435 PMC5409636 28422077

[2] Chisholm D, Sweeny K, Sheehan P, Rasmussen B, Smit F, Cuijpers P, Saxena S (2016). Scaling-up treatment of depression and anxiety: a global return on investment analysis. Lancet Psychiatry.

[3] Onyike CU, Crum RM, Lee HB, Lyketsos CG, Eaton WW (2003). Is obesity associated with major depression? Results from the Third National Health and Nutrition Examination Survey. Am J Epidemiol.

[4] Kivimaki M, Jokela M, Hamer M, Geddes J, Ebmeier K, Kumari M, Singh-Manoux A, Hingorani A, Batty GD (2011). Examining overweight and obesity as risk factors for common mental disorders using fat mass and obesity-associated (FTO) genotype-instrumented analysis: the Whitehall II Study, 1985–2004. Am J Epidemiol.

[5] Hung CF, Rivera M, Craddock N, Owen MJ, Gill M, Korszun A, Maier W, Mors O, Preisig M, Rice JP (2014). Relationship between obesity and the risk of clinically significant depression: Mendelian randomisation study. Br J Psychiatry.

[6] Hartwig FP, Bowden J (2016). Loret de Mola C, Tovo-Rodrigues L, Davey Smith G, Horta BL: Body mass index and psychiatric disorders: a Mendelian randomization study. Sci Rep.

[7] Wray NR, Ripke S, Mattheisen M, Trzaskowski M, Byrne EM, Abdellaoui A, Adams MJ, Agerbo E, Air TM, Andlauer TMF (2018). Genome-wide association analyses identify 44 risk variants and refine the genetic architecture of major depression. Nat Genet.

[8] Tyrrell J, Mulugeta A, Wood AR, Zhou A, Beaumont RN, Tuke MA, Jones SE, Ruth KS, Yaghootkar H, Sharp S (2019). Using genetics to understand the causal influence of higher BMI on depression. Int J Epidemiol.

[9] Speed MS, Jefsen OH, Borglum AD, Speed D, Ostergaard SD (2019). Investigating the association between body fat and depression via Mendelian randomization. Transl Psychiatry.

[10] Dinsa GD, Goryakin Y, Fumagalli E, Suhrcke M (2012). Obesity and socioeconomic status in developing countries: a systematic review. Obes Rev.

[11] Fernald LC (2009). Perception of body weight: a critical factor in understanding obesity in middle-income countries. J Womens Health (Larchmt).

[12] Rguibi M, Belahsen R (2006). Body size preferences and sociocultural influences on attitudes towards obesity among Moroccan Sahraoui women. Body Image.

[13] Holdsworth M, Gartner A, Landais E, Maire B, Delpeuch F (2004). Perceptions of healthy and desirable body size in urban Senegalese women. Int J Obes Relat Metab Disord.

[14] Zeng Q, Yu X (2019). Overweight and obesity standards and subjective well-being: Evidence from China. Econ Hum Biol.

[15] Zhang L, Liu K, Li H, Li D, Chen Z, Zhang LL, Guo LL. Relationship between body mass index and depressive symptoms: the “fat and jolly” hypothesis for the middle-aged and elderly in China. BMC Public Health. 2016;16(1):1201. 10.1186/s12889-016-3864-5 PMC5126817 27894296

[16] Zhi T, Wang Q, Liu Z, Zhu Y, Wang Y, Shi R, Wang Z, Chu X, Wang X, Jiang X (2017). Body mass index, waist circumference and waist-hip ratio are associated with depressive symptoms in older Chinese women: results from the Rugao Longevity and Ageing Study (RuLAS). Aging Ment Health.

[17] Davies NM, Holmes MV, Davey Smith G (2018). Reading Mendelian randomisation studies: a guide, glossary, and checklist for clinicians. BMJ.

[18] Giannakopoulou O, Lin K, Meng X, Su MH, Kuo PH, Peterson RE, Awasthi S, Moscati A, Coleman JRI, Bass N *et al*: The genetic architecture of depression in individuals of East Asian ancestry: a genome-wide association Study. JAMA Psychiatry 2021. 10.1001/jamapsychiatry.2021.2099 PMC8482304 34586374

[19] Bowden J, Davey Smith G, Burgess S (2015). Mendelian randomization with invalid instruments: effect estimation and bias detection through Egger regression. Int J Epidemiol.

[20] Bowden J, Davey Smith G, Haycock PC, Burgess S (2016). Consistent estimation in Mendelian randomization with some invalid instruments using a weighted median estimator. Genet Epidemiol.

[21] de Wit LM, van Straten A, van Herten M, Penninx BW, Cuijpers P (2009). Depression and body mass index, a u-shaped association. BMC Public Health.

[22] Chen Z, Chen J, Collins R, Guo Y, Peto R, Wu F, Li L (2011). China Kadoorie Biobank collaborative g: China Kadoorie Biobank of 0.5 million people: survey methods, baseline characteristics and long-term follow-up. Int J Epidemiol.

[23] Zhou BF (2002). Cooperative Meta-Analysis Group of the Working Group on Obesity in C: Predictive values of body mass index and waist circumference for risk factors of certain related diseases in Chinese adults–study on optimal cut-off points of body mass index and waist circumference in Chinese adults. Biomed Environ Sci.

[24] Zhou BF (2002). Effect of body mass index on all-cause mortality and incidence of cardiovascular diseases–report for meta-analysis of prospective studies open optimal cut-off points of body mass index in Chinese adults. Biomed Environ Sci.

[25] Wu Y (2006). Overweight and obesity in China. BMJ.

[26] Lu J, Huang Y-Q, Liu Z-R, Cao X-L (2015). Validity of Chinese version of the Composite International Diagnostic Interview-3.0 in psychiatric settings. Chin Med J.

[27] Yengo L, Sidorenko J, Kemper KE, Zheng Z, Wood AR, Weedon MN, Frayling TM, Hirschhorn J, Yang J, Visscher PM (2018). Meta-analysis of genome-wide association studies for height and body mass index in ∼700000 individuals of European ancestry. Hum Mol Genet.

[28] Pulit SL, Stoneman C, Morris AP, Wood AR, Glastonbury CA, Tyrrell J, Yengo L, Ferreira T, Marouli E, Ji Y (2019). Meta-analysis of genome-wide association studies for body fat distribution in 694 649 individuals of European ancestry. Hum Mol Genet.

[29] Akiyama M, Okada Y, Kanai M, Takahashi A, Momozawa Y, Ikeda M, Iwata N, Ikegawa S, Hirata M, Matsuda K (2017). Genome-wide association study identifies 112 new loci for body mass index in the Japanese population. Nat Genet.

[30] Milaneschi Y, Simmons WK, van Rossum EFC, Penninx BW (2019). Depression and obesity: evidence of shared biological mechanisms. Mol Psychiatry.

[31] Burgess S, Small DS, Thompson SG (2017). A review of instrumental variable estimators for Mendelian randomization. Stat Methods Med Res.

[32] Guo Z, Small DS (2016). Control function instrumental variable estimation of nonlinear causal effect models. J Mach Learn Res.

[33] Casanova F, O’Loughlin J, Martin S, Beaumont RN, Wood AR, Watkins ER, Freathy RM, Hagenaars SP, Frayling TM, Yaghootkar H (2021). Higher adiposity and mental health: causal inference using Mendelian randomization. Hum Mol Genet.

[34] Yu NW, Chen CY, Liu CY, Chau YL, Chang CM (2011). Association of body mass index and depressive symptoms in a Chinese community population: results from the Health Promotion Knowledge, Attitudes, and Performance Survey in Taiwan. Chang Gung Med J.

[35] Crisp AH, McGuiness B (1976). Jolly fat: relation between obesity and psychoneurosis in general population. Br Med J.

[36] Noh JW, Kim J, Yang Y, Park J, Cheon J, Kwon YD (2017). Body mass index and self-rated health in East Asian countries: comparison among South Korea, China, Japan, and Taiwan. PLoS ONE.

[37] Sato K (2021). Unhappy and happy obesity: a comparative study on the United States and China. J Happiness Stud.

[38] Alimoradi Z, Golboni F, Griffiths MD, Broström A, Lin CY, Pakpour AH (2020). Weight-related stigma and psychological distress: a systematic review and meta-analysis. Clin Nutr.

[39] Brewis A, SturtzSreetharan C, Wutich A (2018). Obesity stigma as a globalizing health challenge. Global Health.

[40] Eisenberg ME, Neumark-Sztainer D, Haines J, Wall M (2006). Weight-teasing and emotional well-being in adolescents: longitudinal findings from Project EAT. J Adolesc Health.

[41] Hunger JM, Major B (2015). Weight stigma mediates the association between BMI and self-reported health. Health Psychol.

[42] Tanenbaum HC, Felicitas JQ, Li Y, Tobias M, Chou CP, Palmer PH, Spruijt-Metz D, Reynolds KD, Anderson Johnson C, Xie B (2016). Overweight perception: associations with weight control goals, attempts, and practices among Chinese female college students. J Acad Nutr Diet.

[43] Mesoudi A (2018). Migration, acculturation, and the maintenance of between-group cultural variation. PLoS ONE.

[44] Smith GD, Ebrahim S (2003). ‘Mendelian randomization’: can genetic epidemiology contribute to understanding environmental determinants of disease?. Int J Epidemiol.

[45] Fewell Z, Davey Smith G, Sterne JA (2007). The impact of residual and unmeasured confounding in epidemiologic studies: a simulation study. Am J Epidemiol.

[46] Morris TT, Davies NM, Hemani G, Smith GD (2020). Population phenomena inflate genetic associations of complex social traits. Sci Adv.

