# Supplementary material for: Mendelian randomisation study of body composition and depression in people of East Asian ancestry highlights potential setting-specific causality
Source: BMC Med. 2023 Feb 1;21:37. doi: 10.1186/s12916-023-02735-8 (PMC9893684; doi:10.1186/s12916-023-02735-8)
Supplement: Supplementary file 1 — Additional file 1: Supplementary methods. [file 12916_2023_2735_MOESM1_ESM.docx]

Mendelian Randomisation study of Body Composition and Depression in people of East Asian Ancestry highlights potential setting-specific causality

Jessica O’Loughlin,^1^* Francesco Casanova,^1^* Zammy Fairhurst-Hunter,^2^ Amanda Hughes,^3^ Jack Bowden,^1^ Edward Watkins,^4^ Rachel M. Freathy,^1^ Iona Y Millwood,^2^ Kuang Lin,^2^ Zhengming Chen,^2^ Liming Li,^5^ Jun Lv,^5^ China Kadoorie Biobank Collaborative Group, Robin Walters,^2^* Laura D Howe,^3^* Karoline Kuchenbaecker,^6^* and Jessica Tyrrell,^1^*

*These authors have contributed equally

^1^Genetics of Complex Traits, The College of Medicine and Health, University of Exeter, United Kingdom

^2^Clinical Trial Service Unit and Epidemiological Studies Unit (CTSU), Nuffield Department of Population Health, University of Oxford, United Kingdom

^3^MRC Integrative Epidemiology Unit (IEU), Population Health Sciences, Bristol Medical School, University of Bristol, United Kingdom

^4^Sir Henry Wellcome Building for Mood Disorders Research, University of Exeter, United Kingdom

^5^Department of Epidemiology and Biostatistics, School of Public Health, Peking University, Beijing, China

^6^Division of Psychiatry, UCL Genetics Institute, University College London, United Kingdom

Corresponding Author: Dr Jessica Tyrrell ([j.tyrrell@exeter.ac.uk](mailto:j.tyrrell@exeter.ac.uk))

Supplementary methods

*Socioeconomic status (SES)*

Several measures of SES were considered in CKB. Firstly, we used a principal component factor analysis to condense data on six related variables, where participants were asked “Do you have a) health cover, b) your own home, c) a private toilet, d) your own phone, e) your own motor vehicle and f) have you had a recent holiday?”, with the option to respond yes (1) or no (0). Two eigenvalues were greater than 1. Here, we use factor one as a measure of SES with higher values representing higher SES. 2. Highest education where participants were asked “What is the highest level of school education you ever received?” with the options of “No formal school”, “Primary school”, “Middle school”, “High school”, “Technical school/college”, or “University”. We created a categorical variable 0 to 5 with 5 representing a higher level of education (University). 3. A categorical household income variable (questionnaire based) representing annual household income of <2,500 yuan, 2,500-4,999 yuan, 5,000-9,999 yuan, 10,000-19,999 yuan, 20,000-34,999 yuan and >=35,000 yuan.

For all three socioeconomic measures, data was available for 100,377 individuals with valid
