# Supplementary material for: Mendelian randomisation study of body composition and depression in people of East Asian ancestry highlights potential setting-specific causality
Source: BMC Med. 2023 Feb 1;21:37. doi: 10.1186/s12916-023-02735-8 (PMC9893684; doi:10.1186/s12916-023-02735-8)
Supplement: Supplementary file 2 — Additional file 2: Supplementary tables S1-S14. Table S1. SNPs used in the 2-sample MR. Tables S2-S4. 2-sampleMendelian Randomisation sensitivity analyses. Table S5. The association betweenthe BMI/WHR GRS and BMI/WHR. Table S6. The observational analyses furtheradjusted for measures of socioeconomic status. Table S7. The observationalassociations between higher BMI and depression classifying individuals ashaving a normal, overweight or obese BMI. Table S8. 2-sample MR results usingthe IVW method. Table S9. 2-sample MR results using MR-Egger, WM and PWM methods.Table S10. The meta-analysed estimates with heterogeneity statistics for1-sample MR analyses. Table S11. Results of the 2-sample MR analysis using theSNPS from the BioBank Japan. Table S12. The meta-analysed non-linear estimatesusing a control function method. Table S13. The observational and 1-sample MRresults of adiposity and major depression. Table S14. The 1-sample MR results removingQingdao with a <1% prevalence of depressive symptoms. [file 12916_2023_2735_MOESM2_ESM.docx]

Table S1. SNPs used in the 2-sample MR.

| **Test_trait** | **chr** | **pos** | **SNP** | **Trait-raising allele** | **Other allele** | **Source** |
| --- | --- | --- | --- | --- | --- | --- |
| BMI | 1 | 1708801 | rs12044597 | A | G | CKB GRS |
| BMI | 1 | 2444414 | rs7535528 | A | G | CKB GRS |
| BMI | 1 | 6715390 | rs6577584 | T | G | CKB GRS |
| BMI | 1 | 8741401 | rs7556169 | A | G | CKB GRS |
| BMI | 1 | 11284336 | rs10779751 | A | G | CKB GRS |
| BMI | 1 | 15966713 | rs17448682 | T | C | CKB GRS |
| BMI | 1 | 16828640 | rs696606 | A | G | CKB GRS |
| BMI | 1 | 17301672 | rs761423 | T | C | CKB GRS |
| BMI | 1 | 19510394 | rs3762396 | A | G | CKB GRS |
| BMI | 1 | 23399932 | rs967605 | T | C | CKB GRS |
| BMI | 1 | 33307987 | rs785278 | A | T | CKB GRS |
| BMI | 1 | 33776728 | rs4653017 | T | C | CKB GRS |
| BMI | 1 | 34602870 | rs9426003 | A | G | CKB GRS |
| BMI | 1 | 39569571 | rs2282231 | T | C | CKB GRS |
| BMI | 1 | 45620134 | rs346722 | T | C | CKB GRS |
| BMI | 1 | 46487552 | rs2275426 | A | G | CKB GRS |
| BMI | 1 | 47700027 | rs6700838 | T | C | CKB GRS |
| BMI | 1 | 49828663 | rs7531656 | A | G | CKB GRS |
| BMI | 1 | 62594677 | rs2481665 | T | C | CKB GRS |
| BMI | 1 | 65987164 | rs11208662 | C | G | CKB GRS |
| BMI | 1 | 72837239 | rs7531118 | T | C | CKB GRS |
| BMI | 1 | 72885281 | rs2590942 | T | G | CKB GRS |
| BMI | 1 | 74997762 | rs12042908 | A | G | CKB GRS |
| BMI | 1 | 77557339 | rs12035349 | A | G | CKB GRS |
| BMI | 1 | 80791708 | rs2154297 | T | C | CKB GRS |
| BMI | 1 | 82379446 | rs284227 | T | C | CKB GRS |
| BMI | 1 | 92976590 | rs6690764 | A | G | CKB GRS |
| BMI | 1 | 96943994 | rs1973993 | T | C | CKB GRS |
| BMI | 1 | 97388226 | rs2030342 | T | C | CKB GRS |
| BMI | 1 | 98320492 | rs4372296 | A | C | CKB GRS |
| BMI | 1 | 101024370 | rs1158103 | A | G | CKB GRS |
| BMI | 1 | 107617707 | rs1730859 | A | G | CKB GRS |
| BMI | 1 | 107885018 | rs12035149 | C | G | CKB GRS |
| BMI | 1 | 107977075 | rs17531363 | A | C | CKB GRS |
| BMI | 1 | 110123971 | rs3768486 | A | G | CKB GRS |
| BMI | 1 | 112318484 | rs12033257 | A | G | CKB GRS |
| BMI | 1 | 118852975 | rs12731372 | T | C | CKB GRS |
| BMI | 1 | 119546842 | rs10923724 | T | C | CKB GRS |
| BMI | 1 | 151018861 | rs6587552 | A | G | CKB GRS |
| BMI | 1 | 154991389 | rs905938 | T | C | CKB GRS |
| BMI | 1 | 155983710 | rs11577179 | A | G | CKB GRS |
| BMI | 1 | 167280354 | rs10733051 | A | G | CKB GRS |
| BMI | 1 | 173473713 | rs912768 | C | G | CKB GRS |
| BMI | 1 | 173549827 | rs12039524 | A | G | CKB GRS |
| BMI | 1 | 174478100 | rs12564992 | A | G | CKB GRS |
| BMI | 1 | 177889480 | rs543874 | A | G | CKB GRS |
| BMI | 1 | 190239907 | rs10920678 | A | G | CKB GRS |
| BMI | 1 | 195047936 | rs12041258 | T | C | CKB GRS |
| BMI | 1 | 197012111 | rs10754210 | A | G | CKB GRS |
| BMI | 1 | 201841476 | rs2820311 | A | G | CKB GRS |
| BMI | 1 | 202116238 | rs9077 | A | G | CKB GRS |
| BMI | 1 | 209543560 | rs17014375 | T | G | CKB GRS |
| BMI | 1 | 219633869 | rs11118308 | A | G | CKB GRS |
| BMI | 1 | 225668524 | rs10915840 | A | G | CKB GRS |
| BMI | 1 | 242986063 | rs2491864 | A | G | CKB GRS |
| BMI | 1 | 243533273 | rs12042959 | A | G | CKB GRS |
| BMI | 1 | 243722892 | rs3753549 | T | C | CKB GRS |
| BMI | 2 | 295255 | rs6548221 | A | G | CKB GRS |
| BMI | 2 | 416815 | rs4639527 | A | G | CKB GRS |
| BMI | 2 | 471514 | rs2724861 | A | G | CKB GRS |
| BMI | 2 | 512490 | rs7425440 | T | C | CKB GRS |
| BMI | 2 | 603088 | rs4854326 | A | G | CKB GRS |
| BMI | 2 | 632348 | rs13021737 | A | G | CKB GRS |
| BMI | 2 | 5830599 | rs3922853 | A | C | CKB GRS |
| BMI | 2 | 6155557 | rs10929925 | A | C | CKB GRS |
| BMI | 2 | 25150296 | rs10182181 | A | G | CKB GRS |
| BMI | 2 | 28973883 | rs4372836 | T | C | CKB GRS |
| BMI | 2 | 35512183 | rs17327461 | T | C | CKB GRS |
| BMI | 2 | 37046657 | rs4670626 | T | C | CKB GRS |
| BMI | 2 | 40291940 | rs6713781 | C | G | CKB GRS |
| BMI | 2 | 46878616 | rs17035438 | A | G | CKB GRS |
| BMI | 2 | 48954905 | rs7561278 | T | C | CKB GRS |
| BMI | 2 | 50233352 | rs930295 | A | C | CKB GRS |
| BMI | 2 | 50735943 | rs7598402 | C | G | CKB GRS |
| BMI | 2 | 50865334 | rs2194385 | A | C | CKB GRS |
| BMI | 2 | 51171962 | rs968972 | A | G | CKB GRS |
| BMI | 2 | 51834839 | rs10168197 | C | G | CKB GRS |
| BMI | 2 | 55281901 | rs7601895 | C | G | CKB GRS |
| BMI | 2 | 56603985 | rs13432055 | T | C | CKB GRS |
| BMI | 2 | 58481863 | rs6753170 | T | C | CKB GRS |
| BMI | 2 | 58792377 | rs929641 | A | G | CKB GRS |
| BMI | 2 | 58935282 | rs4671328 | T | G | CKB GRS |
| BMI | 2 | 59307725 | rs6545714 | A | G | CKB GRS |
| BMI | 2 | 60164634 | rs4671358 | A | T | CKB GRS |
| BMI | 2 | 60285100 | rs980329 | T | C | CKB GRS |
| BMI | 2 | 61307982 | rs12713433 | T | C | CKB GRS |
| BMI | 2 | 61619267 | rs10190332 | T | G | CKB GRS |
| BMI | 2 | 62848319 | rs13417156 | T | C | CKB GRS |
| BMI | 2 | 67825685 | rs2902021 | T | C | CKB GRS |
| BMI | 2 | 69562127 | rs7607351 | T | C | CKB GRS |
| BMI | 2 | 79482643 | rs934515 | A | G | CKB GRS |
| BMI | 2 | 81816251 | rs1371108 | A | C | CKB GRS |
| BMI | 2 | 86766153 | rs7557796 | T | C | CKB GRS |
| BMI | 2 | 100830040 | rs4303732 | T | C | CKB GRS |
| BMI | 2 | 102436738 | rs12615778 | A | G | CKB GRS |
| BMI | 2 | 103496700 | rs10203277 | A | G | CKB GRS |
| BMI | 2 | 105466005 | rs1451533 | A | G | CKB GRS |
| BMI | 2 | 110010962 | rs4676084 | A | G | CKB GRS |
| BMI | 2 | 113955074 | rs902695 | A | G | CKB GRS |
| BMI | 2 | 142293146 | rs17551974 | A | C | CKB GRS |
| BMI | 2 | 143960593 | rs6710871 | A | G | CKB GRS |
| BMI | 2 | 147907202 | rs453520 | T | C | CKB GRS |
| BMI | 2 | 151198990 | rs16828086 | C | G | CKB GRS |
| BMI | 2 | 156018263 | rs7600699 | C | G | CKB GRS |
| BMI | 2 | 172599615 | rs6738445 | T | C | CKB GRS |
| BMI | 2 | 175000711 | rs10930641 | A | G | CKB GRS |
| BMI | 2 | 181607676 | rs9630985 | A | C | CKB GRS |
| BMI | 2 | 188325474 | rs993954 | T | G | CKB GRS |
| BMI | 2 | 198166565 | rs919433 | A | G | CKB GRS |
| BMI | 2 | 198585087 | rs10497807 | C | G | CKB GRS |
| BMI | 2 | 199169278 | rs12991989 | C | G | CKB GRS |
| BMI | 2 | 204012790 | rs7564679 | A | G | CKB GRS |
| BMI | 2 | 206084372 | rs12694021 | A | C | CKB GRS |
| BMI | 2 | 207244783 | rs972540 | A | G | CKB GRS |
| BMI | 2 | 208255518 | rs17203016 | A | G | CKB GRS |
| BMI | 2 | 211608379 | rs4673553 | T | G | CKB GRS |
| BMI | 2 | 211983316 | rs1437929 | A | G | CKB GRS |
| BMI | 2 | 213413231 | rs7599312 | A | G | CKB GRS |
| BMI | 2 | 219279097 | rs7607369 | A | G | CKB GRS |
| BMI | 2 | 220163543 | rs11889536 | A | G | CKB GRS |
| BMI | 2 | 228891702 | rs12479233 | A | T | CKB GRS |
| BMI | 2 | 229016917 | rs10211055 | T | C | CKB GRS |
| BMI | 2 | 230663576 | rs6720868 | T | C | CKB GRS |
| BMI | 3 | 6009092 | rs10510321 | T | C | CKB GRS |
| BMI | 3 | 8138801 | rs1554193 | A | T | CKB GRS |
| BMI | 3 | 9514856 | rs3915844 | A | G | CKB GRS |
| BMI | 3 | 12394840 | rs1899951 | T | C | CKB GRS |
| BMI | 3 | 13345450 | rs580438 | T | C | CKB GRS |
| BMI | 3 | 15873407 | rs11128760 | A | G | CKB GRS |
| BMI | 3 | 20441050 | rs4858193 | T | C | CKB GRS |
| BMI | 3 | 21794904 | rs11711337 | A | T | CKB GRS |
| BMI | 3 | 25106437 | rs6804842 | A | G | CKB GRS |
| BMI | 3 | 28727523 | rs6786125 | C | G | CKB GRS |
| BMI | 3 | 34700713 | rs10865858 | T | C | CKB GRS |
| BMI | 3 | 41310470 | rs9814633 | A | G | CKB GRS |
| BMI | 3 | 42308735 | rs10460960 | A | G | CKB GRS |
| BMI | 3 | 42418446 | rs28350 | A | G | CKB GRS |
| BMI | 3 | 46742019 | rs6442021 | T | C | CKB GRS |
| BMI | 3 | 48130893 | rs6442101 | T | C | CKB GRS |
| BMI | 3 | 48732480 | rs17080319 | T | C | CKB GRS |
| BMI | 3 | 49088112 | rs9846123 | T | C | CKB GRS |
| BMI | 3 | 49237334 | rs12637576 | T | C | CKB GRS |
| BMI | 3 | 49343175 | rs2230929 | A | G | CKB GRS |
| BMI | 3 | 49936102 | rs2230590 | T | C | CKB GRS |
| BMI | 3 | 50080174 | rs12631248 | C | G | CKB GRS |
| BMI | 3 | 50420554 | rs2236950 | A | C | CKB GRS |
| BMI | 3 | 50820486 | rs9838283 | A | G | CKB GRS |
| BMI | 3 | 51184893 | rs9827072 | A | G | CKB GRS |
| BMI | 3 | 51243837 | rs13095652 | T | C | CKB GRS |
| BMI | 3 | 52084040 | rs3821841 | T | C | CKB GRS |
| BMI | 3 | 52815905 | rs2710323 | T | C | CKB GRS |
| BMI | 3 | 53777176 | rs2680648 | T | C | CKB GRS |
| BMI | 3 | 56114861 | rs12488237 | T | C | CKB GRS |
| BMI | 3 | 61236462 | rs2365389 | T | C | CKB GRS |
| BMI | 3 | 62481063 | rs1452075 | T | C | CKB GRS |
| BMI | 3 | 62713143 | rs925018 | C | G | CKB GRS |
| BMI | 3 | 70539559 | rs11915371 | A | C | CKB GRS |
| BMI | 3 | 77624784 | rs775731 | T | C | CKB GRS |
| BMI | 3 | 81792112 | rs3849570 | A | C | CKB GRS |
| BMI | 3 | 85658230 | rs1375561 | T | C | CKB GRS |
| BMI | 3 | 89331055 | rs12638746 | A | G | CKB GRS |
| BMI | 3 | 89392778 | rs9832305 | T | C | CKB GRS |
| BMI | 3 | 89442305 | rs6551410 | A | T | CKB GRS |
| BMI | 3 | 89443784 | rs6803870 | T | C | CKB GRS |
| BMI | 3 | 90428286 | rs9714342 | T | C | CKB GRS |
| BMI | 3 | 94038085 | rs1454687 | C | G | CKB GRS |
| BMI | 3 | 104606130 | rs1436343 | A | G | CKB GRS |
| BMI | 3 | 108119071 | rs4273371 | T | C | CKB GRS |
| BMI | 3 | 115071668 | rs16823670 | A | G | CKB GRS |
| BMI | 3 | 115417865 | rs3772934 | T | C | CKB GRS |
| BMI | 3 | 116937546 | rs6804181 | A | T | CKB GRS |
| BMI | 3 | 119571541 | rs4624596 | T | C | CKB GRS |
| BMI | 3 | 123093541 | rs2124499 | C | G | CKB GRS |
| BMI | 3 | 124687767 | rs1909586 | T | G | CKB GRS |
| BMI | 3 | 131758077 | rs1320903 | A | G | CKB GRS |
| BMI | 3 | 134665159 | rs10935143 | A | G | CKB GRS |
| BMI | 3 | 135621417 | rs1014312 | C | G | CKB GRS |
| BMI | 3 | 136744386 | rs181732 | T | G | CKB GRS |
| BMI | 3 | 136845666 | rs9881036 | A | G | CKB GRS |
| BMI | 3 | 136907111 | rs4678297 | T | C | CKB GRS |
| BMI | 3 | 136979939 | rs1600136 | A | C | CKB GRS |
| BMI | 3 | 137215820 | rs4342060 | T | C | CKB GRS |
| BMI | 3 | 141275436 | rs16851483 | T | G | CKB GRS |
| BMI | 3 | 154034950 | rs355777 | C | G | CKB GRS |
| BMI | 3 | 156299313 | rs7615297 | C | G | CKB GRS |
| BMI | 3 | 156893782 | rs6767619 | C | G | CKB GRS |
| BMI | 3 | 157985182 | rs827092 | T | C | CKB GRS |
| BMI | 3 | 170744815 | rs5396 | T | C | CKB GRS |
| BMI | 3 | 171129859 | rs13085472 | T | C | CKB GRS |
| BMI | 3 | 173095123 | rs39654 | A | G | CKB GRS |
| BMI | 3 | 176341188 | rs1454148 | T | C | CKB GRS |
| BMI | 3 | 183486117 | rs262956 | T | G | CKB GRS |
| BMI | 3 | 184044433 | rs2293605 | T | C | CKB GRS |
| BMI | 3 | 185834499 | rs9816226 | A | T | CKB GRS |
| BMI | 3 | 193639623 | rs13072095 | T | C | CKB GRS |
| BMI | 3 | 196088464 | rs6764533 | A | G | CKB GRS |
| BMI | 4 | 6492739 | rs4524456 | A | G | CKB GRS |
| BMI | 4 | 10491040 | rs7692088 | C | G | CKB GRS |
| BMI | 4 | 16600664 | rs6818414 | T | C | CKB GRS |
| BMI | 4 | 18514827 | rs1477887 | A | G | CKB GRS |
| BMI | 4 | 20263058 | rs1323068 | A | G | CKB GRS |
| BMI | 4 | 25428296 | rs9291467 | T | C | CKB GRS |
| BMI | 4 | 28561990 | rs6448587 | A | C | CKB GRS |
| BMI | 4 | 30843533 | rs1345148 | T | C | CKB GRS |
| BMI | 4 | 38692835 | rs1000096 | T | C | CKB GRS |
| BMI | 4 | 44514468 | rs1866510 | T | C | CKB GRS |
| BMI | 4 | 45182527 | rs10938397 | A | G | CKB GRS |
| BMI | 4 | 49064487 | rs2768950 | A | G | CKB GRS |
| BMI | 4 | 52926216 | rs711347 | A | T | CKB GRS |
| BMI | 4 | 55221467 | rs1492767 | T | C | CKB GRS |
| BMI | 4 | 55505360 | rs2192158 | A | G | CKB GRS |
| BMI | 4 | 60253877 | rs925421 | A | G | CKB GRS |
| BMI | 4 | 65700865 | rs11945861 | A | G | CKB GRS |
| BMI | 4 | 80794681 | rs7674623 | T | C | CKB GRS |
| BMI | 4 | 89054667 | rs4148155 | A | G | CKB GRS |
| BMI | 4 | 91253956 | rs1903579 | C | G | CKB GRS |
| BMI | 4 | 102183594 | rs2850969 | T | C | CKB GRS |
| BMI | 4 | 102708997 | rs7377083 | A | C | CKB GRS |
| BMI | 4 | 103936001 | rs6843738 | A | G | CKB GRS |
| BMI | 4 | 112713436 | rs326889 | T | C | CKB GRS |
| BMI | 4 | 115124089 | rs7694732 | A | G | CKB GRS |
| BMI | 4 | 119101723 | rs1403846 | T | C | CKB GRS |
| BMI | 4 | 130731284 | rs4864201 | T | C | CKB GRS |
| BMI | 4 | 137083193 | rs1296328 | A | C | CKB GRS |
| BMI | 4 | 140881964 | rs769674 | A | T | CKB GRS |
| BMI | 4 | 143663206 | rs331949 | T | C | CKB GRS |
| BMI | 4 | 145986668 | rs1455137 | A | C | CKB GRS |
| BMI | 4 | 147376805 | rs11736228 | A | T | CKB GRS |
| BMI | 4 | 153075491 | rs6827083 | A | G | CKB GRS |
| BMI | 4 | 162129844 | rs13110266 | A | G | CKB GRS |
| BMI | 4 | 163038241 | rs17538472 | T | C | CKB GRS |
| BMI | 4 | 180167906 | rs7683836 | A | G | CKB GRS |
| BMI | 5 | 27185904 | rs4518345 | A | G | CKB GRS |
| BMI | 5 | 43191033 | rs7730004 | T | C | CKB GRS |
| BMI | 5 | 59208302 | rs6879326 | T | C | CKB GRS |
| BMI | 5 | 60712212 | rs6449531 | A | G | CKB GRS |
| BMI | 5 | 63932234 | rs6888159 | C | G | CKB GRS |
| BMI | 5 | 64168193 | rs2367112 | T | G | CKB GRS |
| BMI | 5 | 66175682 | rs25832 | A | G | CKB GRS |
| BMI | 5 | 75003678 | rs2307111 | T | C | CKB GRS |
| BMI | 5 | 80818639 | rs12514473 | T | C | CKB GRS |
| BMI | 5 | 86420392 | rs17591778 | A | G | CKB GRS |
| BMI | 5 | 86727027 | rs7702514 | T | C | CKB GRS |
| BMI | 5 | 86754835 | rs186543 | C | G | CKB GRS |
| BMI | 5 | 86908920 | rs17285919 | T | C | CKB GRS |
| BMI | 5 | 87125578 | rs1423627 | T | C | CKB GRS |
| BMI | 5 | 87932809 | rs4916661 | T | G | CKB GRS |
| BMI | 5 | 87988733 | rs2304607 | A | G | CKB GRS |
| BMI | 5 | 88858208 | rs12655756 | A | T | CKB GRS |
| BMI | 5 | 94206202 | rs159032 | T | C | CKB GRS |
| BMI | 5 | 95728898 | rs6235 | C | G | CKB GRS |
| BMI | 5 | 95856501 | rs2611742 | T | C | CKB GRS |
| BMI | 5 | 95933162 | rs174415 | A | T | CKB GRS |
| BMI | 5 | 96080883 | rs3822683 | A | G | CKB GRS |
| BMI | 5 | 106506697 | rs7710595 | A | C | CKB GRS |
| BMI | 5 | 107439012 | rs40067 | A | G | CKB GRS |
| BMI | 5 | 119372533 | rs6595205 | C | G | CKB GRS |
| BMI | 5 | 122733317 | rs7711753 | A | G | CKB GRS |
| BMI | 5 | 130356413 | rs6877851 | C | G | CKB GRS |
| BMI | 5 | 133865452 | rs329124 | A | G | CKB GRS |
| BMI | 5 | 136571959 | rs13163306 | A | G | CKB GRS |
| BMI | 5 | 137631073 | rs7716275 | T | G | CKB GRS |
| BMI | 5 | 138061341 | rs160401 | T | C | CKB GRS |
| BMI | 5 | 138372345 | rs4643949 | T | C | CKB GRS |
| BMI | 5 | 139080745 | rs13174863 | A | G | CKB GRS |
| BMI | 5 | 140992235 | rs3844598 | A | G | CKB GRS |
| BMI | 5 | 141777439 | rs254428 | T | G | CKB GRS |
| BMI | 5 | 144484261 | rs2190788 | T | G | CKB GRS |
| BMI | 5 | 151254297 | rs10066835 | T | C | CKB GRS |
| BMI | 5 | 152274478 | rs12659802 | A | G | CKB GRS |
| BMI | 5 | 153095918 | rs10035289 | A | G | CKB GRS |
| BMI | 5 | 153537893 | rs7715256 | T | G | CKB GRS |
| BMI | 5 | 157516393 | rs1650586 | T | G | CKB GRS |
| BMI | 5 | 158271680 | rs17056301 | T | C | CKB GRS |
| BMI | 5 | 165185571 | rs7727781 | T | C | CKB GRS |
| BMI | 5 | 167293652 | rs17525725 | A | G | CKB GRS |
| BMI | 5 | 167352783 | rs248139 | A | G | CKB GRS |
| BMI | 5 | 168192944 | rs1014194 | A | C | CKB GRS |
| BMI | 5 | 170459675 | rs7730898 | A | G | CKB GRS |
| BMI | 6 | 9510030 | rs9463175 | T | C | CKB GRS |
| BMI | 6 | 9952059 | rs9396763 | A | C | CKB GRS |
| BMI | 6 | 12124855 | rs2228213 | A | G | CKB GRS |
| BMI | 6 | 13189275 | rs9367368 | T | C | CKB GRS |
| BMI | 6 | 19078274 | rs2842385 | A | G | CKB GRS |
| BMI | 6 | 19211776 | rs9460306 | T | C | CKB GRS |
| BMI | 6 | 20705590 | rs11753081 | T | G | CKB GRS |
| BMI | 6 | 21919387 | rs7760082 | A | G | CKB GRS |
| BMI | 6 | 23876240 | rs6900723 | T | C | CKB GRS |
| BMI | 6 | 27293049 | rs17739298 | C | G | CKB GRS |
| BMI | 6 | 27408181 | rs760880 | T | G | CKB GRS |
| BMI | 6 | 27527205 | rs6908295 | A | C | CKB GRS |
| BMI | 6 | 27859568 | rs200968 | T | C | CKB GRS |
| BMI | 6 | 28059458 | rs3734572 | T | C | CKB GRS |
| BMI | 6 | 28296863 | rs853679 | A | C | CKB GRS |
| BMI | 6 | 31322367 | rs3819299 | T | G | CKB GRS |
| BMI | 6 | 33554147 | rs419261 | T | C | CKB GRS |
| BMI | 6 | 34617144 | rs2814992 | A | G | CKB GRS |
| BMI | 6 | 34644749 | rs12215331 | T | C | CKB GRS |
| BMI | 6 | 34683635 | rs6938239 | A | G | CKB GRS |
| BMI | 6 | 34975415 | rs2140418 | T | C | CKB GRS |
| BMI | 6 | 35033854 | rs820077 | A | G | CKB GRS |
| BMI | 6 | 35512955 | rs3807049 | T | C | CKB GRS |
| BMI | 6 | 35619554 | rs1475774 | A | G | CKB GRS |
| BMI | 6 | 35672330 | rs9394312 | C | G | CKB GRS |
| BMI | 6 | 40080069 | rs847747 | T | G | CKB GRS |
| BMI | 6 | 40348653 | rs2033529 | A | G | CKB GRS |
| BMI | 6 | 41133806 | rs7748777 | A | G | CKB GRS |
| BMI | 6 | 42676480 | rs9349239 | A | G | CKB GRS |
| BMI | 6 | 43757896 | rs998584 | A | C | CKB GRS |
| BMI | 6 | 43757896 | rs998584 | A | C | CKB GRS |
| BMI | 6 | 50566383 | rs2295896 | A | G | CKB GRS |
| BMI | 6 | 50803050 | rs987237 | A | G | CKB GRS |
| BMI | 6 | 50820940 | rs2635727 | T | C | CKB GRS |
| BMI | 6 | 51160682 | rs2504674 | C | G | CKB GRS |
| BMI | 6 | 51546123 | rs9370042 | C | G | CKB GRS |
| BMI | 6 | 51638877 | rs2397061 | T | C | CKB GRS |
| BMI | 6 | 51822297 | rs1266922 | A | G | CKB GRS |
| BMI | 6 | 51825285 | rs1358808 | C | G | CKB GRS |
| BMI | 6 | 51843542 | rs6922855 | A | G | CKB GRS |
| BMI | 6 | 51874745 | rs765332 | T | G | CKB GRS |
| BMI | 6 | 53693410 | rs4278019 | A | T | CKB GRS |
| BMI | 6 | 53990465 | rs816364 | A | G | CKB GRS |
| BMI | 6 | 54724405 | rs1503139 | A | G | CKB GRS |
| BMI | 6 | 55013291 | rs9475173 | A | G | CKB GRS |
| BMI | 6 | 55169801 | rs2653365 | T | C | CKB GRS |
| BMI | 6 | 57642076 | rs12207241 | A | G | CKB GRS |
| BMI | 6 | 57964315 | rs4339513 | T | C | CKB GRS |
| BMI | 6 | 58060143 | rs6459326 | C | G | CKB GRS |
| BMI | 6 | 58298846 | rs926279 | A | G | CKB GRS |
| BMI | 6 | 58705746 | rs6904676 | A | C | CKB GRS |
| BMI | 6 | 69968484 | rs6922214 | A | G | CKB GRS |
| BMI | 6 | 73742334 | rs6921533 | T | C | CKB GRS |
| BMI | 6 | 73922654 | rs9688431 | T | C | CKB GRS |
| BMI | 6 | 83433228 | rs9294260 | A | G | CKB GRS |
| BMI | 6 | 87606842 | rs1853639 | A | G | CKB GRS |
| BMI | 6 | 90296588 | rs9362662 | A | G | CKB GRS |
| BMI | 6 | 93913200 | rs1324110 | C | G | CKB GRS |
| BMI | 6 | 97374850 | rs13203153 | A | G | CKB GRS |
| BMI | 6 | 97753223 | rs13209872 | C | G | CKB GRS |
| BMI | 6 | 98539519 | rs901630 | T | C | CKB GRS |
| BMI | 6 | 104493098 | rs6919443 | A | G | CKB GRS |
| BMI | 6 | 104799007 | rs156151 | C | G | CKB GRS |
| BMI | 6 | 108996963 | rs3800229 | T | G | CKB GRS |
| BMI | 6 | 120213880 | rs2357760 | A | G | CKB GRS |
| BMI | 6 | 124925032 | rs2875762 | C | G | CKB GRS |
| BMI | 6 | 126089285 | rs13209968 | C | G | CKB GRS |
| BMI | 6 | 130349119 | rs6569648 | T | C | CKB GRS |
| BMI | 6 | 131897278 | rs2781668 | T | C | CKB GRS |
| BMI | 6 | 137675541 | rs13201877 | A | G | CKB GRS |
| BMI | 6 | 153381622 | rs2185027 | A | C | CKB GRS |
| BMI | 6 | 154309808 | rs10499276 | T | C | CKB GRS |
| BMI | 6 | 160774459 | rs487060 | T | C | CKB GRS |
| BMI | 7 | 897847 | rs7796608 | A | G | CKB GRS |
| BMI | 7 | 1273845 | rs10275044 | A | T | CKB GRS |
| BMI | 7 | 2103668 | rs6461115 | A | G | CKB GRS |
| BMI | 7 | 3125220 | rs4722398 | T | C | CKB GRS |
| BMI | 7 | 5542513 | rs6463489 | T | C | CKB GRS |
| BMI | 7 | 6418275 | rs7784465 | T | C | CKB GRS |
| BMI | 7 | 17287106 | rs6968554 | A | G | CKB GRS |
| BMI | 7 | 21470536 | rs40245 | A | T | CKB GRS |
| BMI | 7 | 24354300 | rs4307239 | A | G | CKB GRS |
| BMI | 7 | 26526960 | rs12666574 | A | G | CKB GRS |
| BMI | 7 | 27231762 | rs4722672 | T | C | CKB GRS |
| BMI | 7 | 28196413 | rs849135 | A | G | CKB GRS |
| BMI | 7 | 32368524 | rs215632 | A | G | CKB GRS |
| BMI | 7 | 35080931 | rs329277 | T | G | CKB GRS |
| BMI | 7 | 44784697 | rs799449 | T | C | CKB GRS |
| BMI | 7 | 49616203 | rs10269783 | A | G | CKB GRS |
| BMI | 7 | 50564204 | rs3807566 | T | G | CKB GRS |
| BMI | 7 | 69598328 | rs1035010 | T | C | CKB GRS |
| BMI | 7 | 70040558 | rs4718966 | T | C | CKB GRS |
| BMI | 7 | 71888157 | rs993931 | A | G | CKB GRS |
| BMI | 7 | 73058017 | rs7777102 | A | G | CKB GRS |
| BMI | 7 | 74094721 | rs13227433 | T | G | CKB GRS |
| BMI | 7 | 75101065 | rs17207196 | T | C | CKB GRS |
| BMI | 7 | 77055885 | rs740157 | A | G | CKB GRS |
| BMI | 7 | 77417584 | rs1544459 | T | C | CKB GRS |
| BMI | 7 | 77829768 | rs1852006 | A | G | CKB GRS |
| BMI | 7 | 78121458 | rs7805441 | T | C | CKB GRS |
| BMI | 7 | 78144371 | rs6963840 | T | C | CKB GRS |
| BMI | 7 | 93232057 | rs13247665 | T | C | CKB GRS |
| BMI | 7 | 94033031 | rs411717 | T | C | CKB GRS |
| BMI | 7 | 99064466 | rs13240600 | A | G | CKB GRS |
| BMI | 7 | 100804430 | rs1048365 | T | C | CKB GRS |
| BMI | 7 | 103417557 | rs11496125 | T | C | CKB GRS |
| BMI | 7 | 109173373 | rs10953620 | A | C | CKB GRS |
| BMI | 7 | 114349212 | rs12705987 | A | T | CKB GRS |
| BMI | 7 | 121964349 | rs1899689 | T | C | CKB GRS |
| BMI | 7 | 126721231 | rs2283093 | T | C | CKB GRS |
| BMI | 7 | 130466854 | rs972283 | A | G | CKB GRS |
| BMI | 7 | 131619847 | rs1593304 | A | G | CKB GRS |
| BMI | 7 | 131896813 | rs874454 | A | G | CKB GRS |
| BMI | 7 | 137424509 | rs3800649 | A | G | CKB GRS |
| BMI | 7 | 138794149 | rs1814170 | A | T | CKB GRS |
| BMI | 7 | 147668180 | rs11773362 | T | C | CKB GRS |
| BMI | 7 | 150668514 | rs4725984 | T | C | CKB GRS |
| BMI | 8 | 4137396 | rs17069831 | T | C | CKB GRS |
| BMI | 8 | 4288577 | rs1658820 | T | G | CKB GRS |
| BMI | 8 | 8380471 | rs7827182 | C | G | CKB GRS |
| BMI | 8 | 9511654 | rs1394 | A | G | CKB GRS |
| BMI | 8 | 9785503 | rs1399054 | A | G | CKB GRS |
| BMI | 8 | 9790041 | rs497417 | A | T | CKB GRS |
| BMI | 8 | 10626280 | rs12682565 | A | G | CKB GRS |
| BMI | 8 | 10647823 | rs11250076 | A | G | CKB GRS |
| BMI | 8 | 10788875 | rs11783247 | T | C | CKB GRS |
| BMI | 8 | 10864363 | rs9657542 | C | G | CKB GRS |
| BMI | 8 | 11060638 | rs2409730 | A | C | CKB GRS |
| BMI | 8 | 11617240 | rs12458 | A | T | CKB GRS |
| BMI | 8 | 11828200 | rs4841659 | T | C | CKB GRS |
| BMI | 8 | 14095900 | rs13263601 | A | C | CKB GRS |
| BMI | 8 | 14324437 | rs10110727 | A | G | CKB GRS |
| BMI | 8 | 15197115 | rs11987383 | A | C | CKB GRS |
| BMI | 8 | 15536311 | rs2543132 | C | G | CKB GRS |
| BMI | 8 | 20639811 | rs4366093 | T | C | CKB GRS |
| BMI | 8 | 23389571 | rs11781222 | T | C | CKB GRS |
| BMI | 8 | 26334167 | rs1594830 | C | G | CKB GRS |
| BMI | 8 | 27167942 | rs17446091 | T | C | CKB GRS |
| BMI | 8 | 28118130 | rs2100814 | A | G | CKB GRS |
| BMI | 8 | 30856464 | rs1362910 | A | G | CKB GRS |
| BMI | 8 | 34503776 | rs7844647 | T | C | CKB GRS |
| BMI | 8 | 38332318 | rs881301 | T | C | CKB GRS |
| BMI | 8 | 64720693 | rs4737183 | A | G | CKB GRS |
| BMI | 8 | 67202787 | rs16932761 | A | G | CKB GRS |
| BMI | 8 | 73439070 | rs1431659 | A | G | CKB GRS |
| BMI | 8 | 77228222 | rs1405348 | A | G | CKB GRS |
| BMI | 8 | 81375457 | rs16907751 | T | C | CKB GRS |
| BMI | 8 | 85077686 | rs733594 | T | C | CKB GRS |
| BMI | 8 | 85696337 | rs2634047 | C | G | CKB GRS |
| BMI | 8 | 87519542 | rs7006629 | T | C | CKB GRS |
| BMI | 8 | 89461609 | rs1700137 | T | C | CKB GRS |
| BMI | 8 | 95582606 | rs12680842 | A | G | CKB GRS |
| BMI | 8 | 101947453 | rs3134353 | A | T | CKB GRS |
| BMI | 8 | 116559435 | rs3808434 | A | G | CKB GRS |
| BMI | 8 | 116662038 | rs2721965 | A | C | CKB GRS |
| BMI | 8 | 118863061 | rs11781699 | T | C | CKB GRS |
| BMI | 8 | 118946541 | rs10955841 | A | G | CKB GRS |
| BMI | 8 | 132879047 | rs12675063 | A | T | CKB GRS |
| BMI | 8 | 138215228 | rs16906845 | A | G | CKB GRS |
| BMI | 8 | 142630782 | rs903959 | A | T | CKB GRS |
| BMI | 8 | 144910239 | rs4076358 | A | G | CKB GRS |
| BMI | 9 | 6959840 | rs7042372 | A | G | CKB GRS |
| BMI | 9 | 8845911 | rs1865341 | T | C | CKB GRS |
| BMI | 9 | 14651283 | rs11790280 | T | C | CKB GRS |
| BMI | 9 | 15670492 | rs6474945 | T | G | CKB GRS |
| BMI | 9 | 16719445 | rs10962549 | T | C | CKB GRS |
| BMI | 9 | 27612918 | rs483752 | T | C | CKB GRS |
| BMI | 9 | 27777012 | rs7874154 | T | C | CKB GRS |
| BMI | 9 | 28410996 | rs1412235 | C | G | CKB GRS |
| BMI | 9 | 37183628 | rs13290794 | A | G | CKB GRS |
| BMI | 9 | 73791849 | rs2174307 | C | G | CKB GRS |
| BMI | 9 | 81367391 | rs10867256 | T | C | CKB GRS |
| BMI | 9 | 83263089 | rs17351791 | A | C | CKB GRS |
| BMI | 9 | 88897891 | rs3739733 | A | G | CKB GRS |
| BMI | 9 | 92191256 | rs10797115 | T | C | CKB GRS |
| BMI | 9 | 94180627 | rs7869771 | A | C | CKB GRS |
| BMI | 9 | 96482633 | rs4744275 | A | G | CKB GRS |
| BMI | 9 | 101481205 | rs450231 | A | G | CKB GRS |
| BMI | 9 | 103061366 | rs10118701 | A | G | CKB GRS |
| BMI | 9 | 104396304 | rs10989568 | A | G | CKB GRS |
| BMI | 9 | 109072075 | rs7024334 | T | G | CKB GRS |
| BMI | 9 | 111932342 | rs6477694 | T | C | CKB GRS |
| BMI | 9 | 120378483 | rs1928295 | T | C | CKB GRS |
| BMI | 9 | 122631560 | rs7865157 | T | C | CKB GRS |
| BMI | 9 | 126096522 | rs10818810 | A | G | CKB GRS |
| BMI | 9 | 127049237 | rs10818938 | A | G | CKB GRS |
| BMI | 9 | 128616073 | rs4515655 | T | C | CKB GRS |
| BMI | 9 | 129390800 | rs3829849 | T | C | CKB GRS |
| BMI | 9 | 129419025 | rs3902840 | A | G | CKB GRS |
| BMI | 9 | 129467340 | rs13292976 | T | C | CKB GRS |
| BMI | 9 | 129940416 | rs3739555 | T | G | CKB GRS |
| BMI | 9 | 131027982 | rs7871866 | C | G | CKB GRS |
| BMI | 9 | 133783566 | rs4740383 | A | G | CKB GRS |
| BMI | 9 | 140646121 | rs11792069 | A | G | CKB GRS |
| BMI | 10 | 2585792 | rs11251352 | A | G | CKB GRS |
| BMI | 10 | 16750129 | rs7893571 | T | G | CKB GRS |
| BMI | 10 | 19776828 | rs12776880 | A | T | CKB GRS |
| BMI | 10 | 21821274 | rs7084454 | A | G | CKB GRS |
| BMI | 10 | 27318776 | rs3781099 | T | C | CKB GRS |
| BMI | 10 | 33862727 | rs3851083 | A | G | CKB GRS |
| BMI | 10 | 53680085 | rs1937684 | A | T | CKB GRS |
| BMI | 10 | 65314711 | rs2163188 | C | G | CKB GRS |
| BMI | 10 | 76047464 | rs12098284 | T | C | CKB GRS |
| BMI | 10 | 88096047 | rs10887578 | C | G | CKB GRS |
| BMI | 10 | 93032943 | rs2631681 | T | C | CKB GRS |
| BMI | 10 | 99032375 | rs793520 | A | G | CKB GRS |
| BMI | 10 | 99769388 | rs577525 | T | C | CKB GRS |
| BMI | 10 | 100017453 | rs1983864 | T | G | CKB GRS |
| BMI | 10 | 102395440 | rs17094222 | T | C | CKB GRS |
| BMI | 10 | 102452136 | rs11190661 | T | C | CKB GRS |
| BMI | 10 | 102635475 | rs10883553 | A | C | CKB GRS |
| BMI | 10 | 103206115 | rs9787495 | A | G | CKB GRS |
| BMI | 10 | 103984060 | rs7083450 | T | C | CKB GRS |
| BMI | 10 | 104412049 | rs10883759 | A | G | CKB GRS |
| BMI | 10 | 104685299 | rs12411886 | A | C | CKB GRS |
| BMI | 10 | 105033015 | rs1712517 | T | C | CKB GRS |
| BMI | 10 | 105675946 | rs9419958 | T | C | CKB GRS |
| BMI | 10 | 114758349 | rs7903146 | T | C | CKB GRS |
| BMI | 10 | 118672531 | rs10886017 | A | C | CKB GRS |
| BMI | 10 | 125220036 | rs845084 | A | G | CKB GRS |
| BMI | 10 | 126594078 | rs17636031 | T | C | CKB GRS |
| BMI | 11 | 2234690 | rs10840606 | A | G | CKB GRS |
| BMI | 11 | 8694073 | rs12575252 | C | G | CKB GRS |
| BMI | 11 | 11796727 | rs1037587 | T | C | CKB GRS |
| BMI | 11 | 13294268 | rs900144 | T | C | CKB GRS |
| BMI | 11 | 17394073 | rs10832778 | C | G | CKB GRS |
| BMI | 11 | 27677041 | rs7124442 | T | C | CKB GRS |
| BMI | 11 | 27679916 | rs6265 | T | C | CKB GRS |
| BMI | 11 | 28629115 | rs11030385 | A | G | CKB GRS |
| BMI | 11 | 28763321 | rs7948120 | T | C | CKB GRS |
| BMI | 11 | 29158495 | rs1552717 | A | T | CKB GRS |
| BMI | 11 | 30243868 | rs1782507 | T | G | CKB GRS |
| BMI | 11 | 32131303 | rs223051 | T | C | CKB GRS |
| BMI | 11 | 43551416 | rs10838122 | T | C | CKB GRS |
| BMI | 11 | 43728534 | rs12577642 | A | T | CKB GRS |
| BMI | 11 | 45706453 | rs10769165 | T | C | CKB GRS |
| BMI | 11 | 46422686 | rs12574668 | A | C | CKB GRS |
| BMI | 11 | 46520302 | rs17197116 | T | C | CKB GRS |
| BMI | 11 | 46895378 | rs11039014 | A | G | CKB GRS |
| BMI | 11 | 47529947 | rs7124681 | A | C | CKB GRS |
| BMI | 11 | 47836302 | rs7131262 | A | T | CKB GRS |
| BMI | 11 | 48286256 | rs10838852 | T | C | CKB GRS |
| BMI | 11 | 48630323 | rs7478904 | T | C | CKB GRS |
| BMI | 11 | 48901553 | rs1473579 | A | G | CKB GRS |
| BMI | 11 | 49459474 | rs7120873 | T | C | CKB GRS |
| BMI | 11 | 49620595 | rs7924371 | T | C | CKB GRS |
| BMI | 11 | 49994823 | rs10839472 | T | C | CKB GRS |
| BMI | 11 | 55091574 | rs10459012 | A | C | CKB GRS |
| BMI | 11 | 55323307 | rs551137 | T | C | CKB GRS |
| BMI | 11 | 56206141 | rs4939051 | C | G | CKB GRS |
| BMI | 11 | 56446833 | rs4542429 | T | C | CKB GRS |
| BMI | 11 | 56973793 | rs1943477 | T | C | CKB GRS |
| BMI | 11 | 64082807 | rs11600990 | T | C | CKB GRS |
| BMI | 11 | 65594820 | rs7102454 | T | C | CKB GRS |
| BMI | 11 | 65886662 | rs524281 | A | C | CKB GRS |
| BMI | 11 | 66662731 | rs7122539 | A | G | CKB GRS |
| BMI | 11 | 69299771 | rs587230 | A | G | CKB GRS |
| BMI | 11 | 69481969 | rs1789165 | A | G | CKB GRS |
| BMI | 11 | 70563286 | rs2440885 | A | G | CKB GRS |
| BMI | 11 | 72444583 | rs7123876 | T | C | CKB GRS |
| BMI | 11 | 78040259 | rs7117238 | A | G | CKB GRS |
| BMI | 11 | 86133416 | rs1452134 | T | C | CKB GRS |
| BMI | 11 | 89966202 | rs10830452 | A | G | CKB GRS |
| BMI | 11 | 93221105 | rs2605603 | A | G | CKB GRS |
| BMI | 11 | 113234679 | rs719802 | T | C | CKB GRS |
| BMI | 11 | 115044850 | rs1048932 | A | C | CKB GRS |
| BMI | 11 | 115623272 | rs12417072 | A | G | CKB GRS |
| BMI | 11 | 117017530 | rs12420725 | A | G | CKB GRS |
| BMI | 11 | 117267884 | rs573455 | A | G | CKB GRS |
| BMI | 11 | 118913993 | rs1003081 | T | C | CKB GRS |
| BMI | 11 | 121942512 | rs4936671 | C | G | CKB GRS |
| BMI | 11 | 122522375 | rs7941030 | T | C | CKB GRS |
| BMI | 11 | 122765667 | rs3134438 | A | C | CKB GRS |
| BMI | 11 | 131957293 | rs1625427 | T | C | CKB GRS |
| BMI | 11 | 132641959 | rs4936175 | T | C | CKB GRS |
| BMI | 11 | 133827733 | rs3802924 | A | C | CKB GRS |
| BMI | 11 | 134601012 | rs12364470 | T | G | CKB GRS |
| BMI | 12 | 939480 | rs11611246 | T | G | CKB GRS |
| BMI | 12 | 2152655 | rs2429150 | A | C | CKB GRS |
| BMI | 12 | 14413931 | rs12422552 | C | G | CKB GRS |
| BMI | 12 | 17212881 | rs10744146 | A | G | CKB GRS |
| BMI | 12 | 17360254 | rs10840674 | A | G | CKB GRS |
| BMI | 12 | 18789007 | rs621042 | A | C | CKB GRS |
| BMI | 12 | 20473758 | rs7134375 | A | C | CKB GRS |
| BMI | 12 | 23067302 | rs2467110 | T | C | CKB GRS |
| BMI | 12 | 24060075 | rs10842240 | C | G | CKB GRS |
| BMI | 12 | 33379440 | rs10772055 | C | G | CKB GRS |
| BMI | 12 | 38242029 | rs12306932 | T | C | CKB GRS |
| BMI | 12 | 38529333 | rs17096549 | A | G | CKB GRS |
| BMI | 12 | 39159190 | rs6580755 | T | C | CKB GRS |
| BMI | 12 | 39329294 | rs7958206 | A | G | CKB GRS |
| BMI | 12 | 39428802 | rs10876418 | T | C | CKB GRS |
| BMI | 12 | 39982413 | rs11172702 | A | G | CKB GRS |
| BMI | 12 | 41880909 | rs2733287 | A | C | CKB GRS |
| BMI | 12 | 48180508 | rs2240108 | T | C | CKB GRS |
| BMI | 12 | 49987929 | rs7965658 | A | G | CKB GRS |
| BMI | 12 | 50247468 | rs7138803 | A | G | CKB GRS |
| BMI | 12 | 51593616 | rs4077093 | T | G | CKB GRS |
| BMI | 12 | 54653258 | rs4759073 | A | G | CKB GRS |
| BMI | 12 | 56508409 | rs4759228 | C | G | CKB GRS |
| BMI | 12 | 60964108 | rs7975187 | A | G | CKB GRS |
| BMI | 12 | 68205604 | rs1819844 | A | G | CKB GRS |
| BMI | 12 | 69642315 | rs10878946 | T | C | CKB GRS |
| BMI | 12 | 82465797 | rs11115176 | T | C | CKB GRS |
| BMI | 12 | 89899912 | rs7313924 | C | G | CKB GRS |
| BMI | 12 | 90595383 | rs2731222 | A | C | CKB GRS |
| BMI | 12 | 97586257 | rs10745785 | T | C | CKB GRS |
| BMI | 12 | 99560183 | rs651548 | A | G | CKB GRS |
| BMI | 12 | 103658096 | rs4764949 | A | G | CKB GRS |
| BMI | 12 | 110046698 | rs17608150 | T | C | CKB GRS |
| BMI | 12 | 111768973 | rs6490055 | A | G | CKB GRS |
| BMI | 12 | 111780998 | rs1558236 | C | G | CKB GRS |
| BMI | 12 | 111800258 | rs3809272 | A | G | CKB GRS |
| BMI | 12 | 112019799 | rs12369009 | T | G | CKB GRS |
| BMI | 12 | 112771063 | rs10850031 | T | G | CKB GRS |
| BMI | 12 | 114437708 | rs4766710 | A | G | CKB GRS |
| BMI | 12 | 117579274 | rs884282 | T | C | CKB GRS |
| BMI | 12 | 118409640 | rs7973955 | A | G | CKB GRS |
| BMI | 12 | 124409502 | rs7133378 | A | G | CKB GRS |
| BMI | 12 | 124506631 | rs10773049 | T | C | CKB GRS |
| BMI | 12 | 133481917 | rs7968230 | A | G | CKB GRS |
| BMI | 13 | 28011963 | rs1218822 | A | G | CKB GRS |
| BMI | 13 | 28047269 | rs1006353 | A | G | CKB GRS |
| BMI | 13 | 28620036 | rs9507983 | T | C | CKB GRS |
| BMI | 13 | 28681228 | rs9554263 | C | G | CKB GRS |
| BMI | 13 | 31033232 | rs1045411 | T | C | CKB GRS |
| BMI | 13 | 33184288 | rs9595908 | T | C | CKB GRS |
| BMI | 13 | 36230485 | rs9544915 | T | C | CKB GRS |
| BMI | 13 | 40783323 | rs9603697 | T | C | CKB GRS |
| BMI | 13 | 54102206 | rs12429545 | A | G | CKB GRS |
| BMI | 13 | 54694130 | rs10467530 | C | G | CKB GRS |
| BMI | 13 | 58402479 | rs9527706 | A | G | CKB GRS |
| BMI | 13 | 59178258 | rs9538141 | A | G | CKB GRS |
| BMI | 13 | 59425111 | rs1333423 | A | T | CKB GRS |
| BMI | 13 | 65884191 | rs892261 | T | C | CKB GRS |
| BMI | 13 | 66205704 | rs9540493 | A | G | CKB GRS |
| BMI | 13 | 67472713 | rs9571687 | A | C | CKB GRS |
| BMI | 13 | 76386075 | rs629443 | T | G | CKB GRS |
| BMI | 13 | 78371890 | rs1668633 | T | C | CKB GRS |
| BMI | 13 | 79563749 | rs9530843 | A | C | CKB GRS |
| BMI | 13 | 96922191 | rs1927790 | T | C | CKB GRS |
| BMI | 13 | 99120484 | rs7334078 | T | C | CKB GRS |
| BMI | 13 | 111984244 | rs2479958 | A | G | CKB GRS |
| BMI | 14 | 25928179 | rs10132280 | A | C | CKB GRS |
| BMI | 14 | 29680331 | rs4981693 | A | G | CKB GRS |
| BMI | 14 | 29736838 | rs12885454 | A | C | CKB GRS |
| BMI | 14 | 33302882 | rs17522122 | T | G | CKB GRS |
| BMI | 14 | 40101060 | rs1956151 | A | G | CKB GRS |
| BMI | 14 | 47272423 | rs12587412 | T | G | CKB GRS |
| BMI | 14 | 62360464 | rs217671 | A | G | CKB GRS |
| BMI | 14 | 65426216 | rs2412107 | T | G | CKB GRS |
| BMI | 14 | 65910844 | rs11844682 | C | G | CKB GRS |
| BMI | 14 | 69789755 | rs3902951 | T | G | CKB GRS |
| BMI | 14 | 72269668 | rs1205106 | A | G | CKB GRS |
| BMI | 14 | 77529783 | rs17105272 | T | C | CKB GRS |
| BMI | 14 | 79499850 | rs10146527 | T | C | CKB GRS |
| BMI | 14 | 79903993 | rs2003616 | T | G | CKB GRS |
| BMI | 14 | 82684748 | rs799132 | A | T | CKB GRS |
| BMI | 14 | 91512339 | rs1951455 | T | C | CKB GRS |
| BMI | 14 | 92428410 | rs2160077 | A | G | CKB GRS |
| BMI | 14 | 97258752 | rs10131890 | A | C | CKB GRS |
| BMI | 14 | 99671788 | rs3850422 | A | G | CKB GRS |
| BMI | 14 | 101539384 | rs7147503 | T | C | CKB GRS |
| BMI | 14 | 103246470 | rs3803286 | A | G | CKB GRS |
| BMI | 14 | 103862322 | rs2010281 | A | G | CKB GRS |
| BMI | 15 | 27040082 | rs4906908 | T | G | CKB GRS |
| BMI | 15 | 31843528 | rs4284600 | T | C | CKB GRS |
| BMI | 15 | 36402716 | rs8036040 | A | C | CKB GRS |
| BMI | 15 | 41751678 | rs316611 | T | C | CKB GRS |
| BMI | 15 | 46584787 | rs12439798 | T | G | CKB GRS |
| BMI | 15 | 51748610 | rs3736485 | A | G | CKB GRS |
| BMI | 15 | 57120989 | rs1657930 | A | G | CKB GRS |
| BMI | 15 | 60908307 | rs340025 | T | C | CKB GRS |
| BMI | 15 | 61445514 | rs8033510 | T | C | CKB GRS |
| BMI | 15 | 62150364 | rs17238110 | A | G | CKB GRS |
| BMI | 15 | 62316035 | rs12595158 | T | C | CKB GRS |
| BMI | 15 | 64849904 | rs6494481 | T | G | CKB GRS |
| BMI | 15 | 66741387 | rs11629783 | C | G | CKB GRS |
| BMI | 15 | 68103632 | rs16951319 | T | C | CKB GRS |
| BMI | 15 | 68104367 | rs13329567 | T | C | CKB GRS |
| BMI | 15 | 73093991 | rs7164727 | T | C | CKB GRS |
| BMI | 15 | 76150965 | rs2593280 | A | G | CKB GRS |
| BMI | 15 | 76755506 | rs403656 | A | G | CKB GRS |
| BMI | 15 | 76863838 | rs2459359 | C | G | CKB GRS |
| BMI | 15 | 77156899 | rs10519151 | A | T | CKB GRS |
| BMI | 15 | 77207277 | rs4886506 | T | G | CKB GRS |
| BMI | 15 | 77254544 | rs12148386 | T | C | CKB GRS |
| BMI | 15 | 77799657 | rs4886869 | A | G | CKB GRS |
| BMI | 15 | 77915282 | rs8024932 | T | G | CKB GRS |
| BMI | 15 | 78012618 | rs11855853 | T | C | CKB GRS |
| BMI | 15 | 78117685 | rs6495252 | T | C | CKB GRS |
| BMI | 15 | 79432359 | rs12595749 | A | G | CKB GRS |
| BMI | 15 | 81058652 | rs12593036 | A | G | CKB GRS |
| BMI | 15 | 84580156 | rs11259933 | A | G | CKB GRS |
| BMI | 15 | 89928189 | rs150353 | T | G | CKB GRS |
| BMI | 15 | 92570921 | rs12101393 | C | G | CKB GRS |
| BMI | 15 | 95271404 | rs7181498 | T | C | CKB GRS |
| BMI | 16 | 387867 | rs11866815 | T | C | CKB GRS |
| BMI | 16 | 2097158 | rs2516739 | A | G | CKB GRS |
| BMI | 16 | 3599655 | rs12448257 | A | G | CKB GRS |
| BMI | 16 | 3730613 | rs3794702 | A | T | CKB GRS |
| BMI | 16 | 4015729 | rs879620 | T | C | CKB GRS |
| BMI | 16 | 6509009 | rs2534760 | A | T | CKB GRS |
| BMI | 16 | 6701400 | rs10083803 | T | C | CKB GRS |
| BMI | 16 | 9724750 | rs977540 | A | G | CKB GRS |
| BMI | 16 | 15129459 | rs4985155 | A | G | CKB GRS |
| BMI | 16 | 20050466 | rs868554 | C | G | CKB GRS |
| BMI | 16 | 20255123 | rs11074446 | T | C | CKB GRS |
| BMI | 16 | 20375351 | rs9931967 | T | G | CKB GRS |
| BMI | 16 | 23833071 | rs9927848 | A | C | CKB GRS |
| BMI | 16 | 24578458 | rs7195386 | T | C | CKB GRS |
| BMI | 16 | 24803620 | rs1862451 | A | G | CKB GRS |
| BMI | 16 | 28857645 | rs7187776 | A | G | CKB GRS |
| BMI | 16 | 29833714 | rs1057452 | A | G | CKB GRS |
| BMI | 16 | 29994922 | rs3814883 | T | C | CKB GRS |
| BMI | 16 | 31141993 | rs1549293 | T | C | CKB GRS |
| BMI | 16 | 49062590 | rs2080454 | A | C | CKB GRS |
| BMI | 16 | 50986308 | rs1564981 | A | G | CKB GRS |
| BMI | 16 | 51926509 | rs17795934 | T | C | CKB GRS |
| BMI | 16 | 52548037 | rs12443621 | A | G | CKB GRS |
| BMI | 16 | 53770578 | rs16952479 | A | T | CKB GRS |
| BMI | 16 | 53798523 | rs8047395 | A | G | CKB GRS |
| BMI | 16 | 53805344 | rs11075986 | C | G | CKB GRS |
| BMI | 16 | 53818708 | rs3751813 | T | G | CKB GRS |
| BMI | 16 | 53825238 | rs9931164 | A | G | CKB GRS |
| BMI | 16 | 53831146 | rs9922708 | T | C | CKB GRS |
| BMI | 16 | 54153099 | rs2075205 | A | T | CKB GRS |
| BMI | 16 | 54234492 | rs907011 | T | G | CKB GRS |
| BMI | 16 | 56489343 | rs12448738 | A | C | CKB GRS |
| BMI | 16 | 62803841 | rs11075489 | T | C | CKB GRS |
| BMI | 16 | 67316600 | rs7200919 | A | G | CKB GRS |
| BMI | 16 | 67420603 | rs12920590 | T | C | CKB GRS |
| BMI | 16 | 68295598 | rs2863981 | A | G | CKB GRS |
| BMI | 16 | 68381978 | rs2307022 | A | G | CKB GRS |
| BMI | 16 | 69174141 | rs10500548 | T | C | CKB GRS |
| BMI | 16 | 69556715 | rs889398 | T | C | CKB GRS |
| BMI | 16 | 70514828 | rs7919 | A | C | CKB GRS |
| BMI | 16 | 71899586 | rs11642001 | A | G | CKB GRS |
| BMI | 16 | 71965915 | rs952159 | A | G | CKB GRS |
| BMI | 16 | 72996162 | rs756717 | A | G | CKB GRS |
| BMI | 16 | 73606563 | rs825680 | A | T | CKB GRS |
| BMI | 16 | 76779612 | rs6564360 | A | G | CKB GRS |
| BMI | 16 | 80752293 | rs8046061 | T | C | CKB GRS |
| BMI | 16 | 82438337 | rs12922346 | C | G | CKB GRS |
| BMI | 16 | 82650384 | rs4783241 | C | G | CKB GRS |
| BMI | 16 | 82872628 | rs7206608 | C | G | CKB GRS |
| BMI | 17 | 1846831 | rs4516268 | A | C | CKB GRS |
| BMI | 17 | 2136065 | rs7217226 | T | G | CKB GRS |
| BMI | 17 | 5283252 | rs1000940 | A | G | CKB GRS |
| BMI | 17 | 5412361 | rs8079034 | T | C | CKB GRS |
| BMI | 17 | 15943910 | rs1075901 | T | C | CKB GRS |
| BMI | 17 | 21261560 | rs4986044 | T | C | CKB GRS |
| BMI | 17 | 28074563 | rs1038088 | T | G | CKB GRS |
| BMI | 17 | 29349688 | rs8067737 | T | C | CKB GRS |
| BMI | 17 | 31460899 | rs7211567 | T | C | CKB GRS |
| BMI | 17 | 31747629 | rs12453418 | A | G | CKB GRS |
| BMI | 17 | 34942595 | rs1106908 | A | G | CKB GRS |
| BMI | 17 | 35057883 | rs4796243 | A | G | CKB GRS |
| BMI | 17 | 38160754 | rs8070454 | T | C | CKB GRS |
| BMI | 17 | 39573713 | rs16966801 | A | G | CKB GRS |
| BMI | 17 | 42935059 | rs8069296 | T | C | CKB GRS |
| BMI | 17 | 46051911 | rs886444 | A | G | CKB GRS |
| BMI | 17 | 46252346 | rs208015 | T | C | CKB GRS |
| BMI | 17 | 46669430 | rs9299 | T | C | CKB GRS |
| BMI | 17 | 47090785 | rs11079849 | T | C | CKB GRS |
| BMI | 17 | 51923847 | rs10515050 | T | C | CKB GRS |
| BMI | 17 | 55336155 | rs8071182 | A | G | CKB GRS |
| BMI | 17 | 59497277 | rs757608 | A | G | CKB GRS |
| BMI | 17 | 61728881 | rs8075273 | A | C | CKB GRS |
| BMI | 17 | 65694355 | rs2537847 | A | G | CKB GRS |
| BMI | 17 | 65870073 | rs12602912 | T | C | CKB GRS |
| BMI | 17 | 71754545 | rs2619976 | T | C | CKB GRS |
| BMI | 17 | 73759552 | rs7209235 | A | G | CKB GRS |
| BMI | 17 | 77796889 | rs1285245 | C | G | CKB GRS |
| BMI | 17 | 78611724 | rs12939549 | A | G | CKB GRS |
| BMI | 17 | 78640510 | rs4889782 | T | C | CKB GRS |
| BMI | 17 | 79081724 | rs4969387 | C | G | CKB GRS |
| BMI | 17 | 79202329 | rs1048775 | C | G | CKB GRS |
| BMI | 17 | 80052073 | rs9905991 | A | G | CKB GRS |
| BMI | 18 | 947954 | rs1608445 | A | G | CKB GRS |
| BMI | 18 | 6873954 | rs1241986 | A | G | CKB GRS |
| BMI | 18 | 21116998 | rs12964689 | A | G | CKB GRS |
| BMI | 18 | 23178748 | rs273697 | A | G | CKB GRS |
| BMI | 18 | 31251276 | rs1941697 | A | G | CKB GRS |
| BMI | 18 | 31581247 | rs16965062 | T | C | CKB GRS |
| BMI | 18 | 36182440 | rs1365466 | T | C | CKB GRS |
| BMI | 18 | 37103550 | rs1791253 | T | G | CKB GRS |
| BMI | 18 | 40992698 | rs555267 | T | G | CKB GRS |
| BMI | 18 | 42598463 | rs954018 | A | G | CKB GRS |
| BMI | 18 | 42950629 | rs10438964 | T | C | CKB GRS |
| BMI | 18 | 45921214 | rs7239114 | A | G | CKB GRS |
| BMI | 18 | 51478026 | rs1498139 | A | C | CKB GRS |
| BMI | 18 | 52479487 | rs8092503 | A | G | CKB GRS |
| BMI | 18 | 56883319 | rs7243357 | T | G | CKB GRS |
| BMI | 18 | 57677294 | rs2000746 | A | G | CKB GRS |
| BMI | 18 | 57726627 | rs12327272 | A | G | CKB GRS |
| BMI | 18 | 57741783 | rs1942866 | C | G | CKB GRS |
| BMI | 18 | 57804346 | rs8095404 | A | T | CKB GRS |
| BMI | 18 | 57838401 | rs663129 | A | G | CKB GRS |
| BMI | 18 | 57853056 | rs9961813 | A | C | CKB GRS |
| BMI | 18 | 57969244 | rs9675376 | A | G | CKB GRS |
| BMI | 18 | 58039276 | rs2229616 | T | C | CKB GRS |
| BMI | 18 | 58371566 | rs8087550 | A | C | CKB GRS |
| BMI | 18 | 60739250 | rs9951893 | T | C | CKB GRS |
| BMI | 18 | 63297672 | rs2012927 | A | G | CKB GRS |
| BMI | 18 | 69224478 | rs8089514 | A | T | CKB GRS |
| BMI | 18 | 73498528 | rs11150911 | A | C | CKB GRS |
| BMI | 18 | 76742544 | rs1787267 | C | G | CKB GRS |
| BMI | 19 | 12994140 | rs12609744 | T | C | CKB GRS |
| BMI | 19 | 18215247 | rs273504 | A | G | CKB GRS |
| BMI | 19 | 18454825 | rs17724992 | A | G | CKB GRS |
| BMI | 19 | 19789528 | rs2304130 | A | G | CKB GRS |
| BMI | 19 | 30296853 | rs8102137 | T | C | CKB GRS |
| BMI | 19 | 30683879 | rs2866816 | T | C | CKB GRS |
| BMI | 19 | 33963766 | rs10408013 | T | C | CKB GRS |
| BMI | 19 | 34311481 | rs29938 | T | C | CKB GRS |
| BMI | 19 | 42637232 | rs3826705 | T | C | CKB GRS |
| BMI | 19 | 45395619 | rs2075650 | A | G | CKB GRS |
| BMI | 19 | 46180184 | rs11672660 | T | C | CKB GRS |
| BMI | 19 | 47569003 | rs3810291 | A | G | CKB GRS |
| BMI | 20 | 1410582 | rs1884389 | T | C | CKB GRS |
| BMI | 20 | 3026069 | rs676749 | A | T | CKB GRS |
| BMI | 20 | 6612832 | rs1884897 | A | G | CKB GRS |
| BMI | 20 | 15801600 | rs12480713 | T | C | CKB GRS |
| BMI | 20 | 15819495 | rs8123881 | A | G | CKB GRS |
| BMI | 20 | 16564210 | rs4814512 | A | C | CKB GRS |
| BMI | 20 | 25059442 | rs6138482 | T | C | CKB GRS |
| BMI | 20 | 25971327 | rs2386802 | A | C | CKB GRS |
| BMI | 20 | 26073030 | rs6132918 | T | C | CKB GRS |
| BMI | 20 | 30504530 | rs8121840 | A | G | CKB GRS |
| BMI | 20 | 30785593 | rs6121381 | A | T | CKB GRS |
| BMI | 20 | 31097877 | rs293566 | T | C | CKB GRS |
| BMI | 20 | 32542814 | rs13041173 | A | G | CKB GRS |
| BMI | 20 | 32606299 | rs17091470 | T | G | CKB GRS |
| BMI | 20 | 32686658 | rs6142096 | A | G | CKB GRS |
| BMI | 20 | 32738335 | rs1015363 | A | G | CKB GRS |
| BMI | 20 | 32738612 | rs1015362 | T | C | CKB GRS |
| BMI | 20 | 33170752 | rs6088529 | A | C | CKB GRS |
| BMI | 20 | 33594226 | rs6060151 | T | G | CKB GRS |
| BMI | 20 | 33703607 | rs3746429 | T | C | CKB GRS |
| BMI | 20 | 34025756 | rs143384 | A | G | CKB GRS |
| BMI | 20 | 41987392 | rs2143253 | A | G | CKB GRS |
| BMI | 20 | 44914134 | rs2425857 | A | G | CKB GRS |
| BMI | 20 | 47495560 | rs6019482 | T | C | CKB GRS |
| BMI | 20 | 51107290 | rs17806379 | T | C | CKB GRS |
| BMI | 20 | 54157497 | rs559267 | A | G | CKB GRS |
| BMI | 20 | 61530915 | rs6011457 | A | T | CKB GRS |
| BMI | 20 | 62127121 | rs310618 | T | C | CKB GRS |
| BMI | 20 | 62380542 | rs12625413 | T | C | CKB GRS |
| BMI | 20 | 62522315 | rs8567 | A | G | CKB GRS |
| BMI | 21 | 34153330 | rs9979651 | C | G | CKB GRS |
| BMI | 21 | 39238610 | rs762147 | A | G | CKB GRS |
| BMI | 21 | 40309436 | rs13047416 | C | G | CKB GRS |
| BMI | 21 | 40627020 | rs2836961 | A | C | CKB GRS |
| BMI | 21 | 42653567 | rs2838006 | T | C | CKB GRS |
| BMI | 21 | 46570896 | rs427943 | A | C | CKB GRS |
| BMI | 22 | 40604945 | rs4820408 | T | G | CKB GRS |
| BMI | 22 | 40640285 | rs5750913 | A | G | CKB GRS |
| BMI | 22 | 48875699 | rs9615905 | T | C | CKB GRS |
| BMI | 1 | 27971092 | rs2076463 | A | G | BBJ |
| BMI | 1 | 155767708 | rs860295 | A | G | BBJ |
| BMI | 1 | 190308834 | rs491055 | A | G | BBJ |
| BMI | 2 | 37559355 | rs6734118 | C | A | BBJ |
| BMI | 2 | 38750287 | rs77489951 | C | T | BBJ |
| BMI | 2 | 51195601 | rs10174398 | T | C | BBJ |
| BMI | 2 | 54161363 | rs10208649 | T | C | BBJ |
| BMI | 2 | 169091942 | rs2390669 | A | C | BBJ |
| BMI | 3 | 11655381 | rs2574704 | T | C | BBJ |
| BMI | 3 | 42299399 | rs8192473 | C | T | BBJ |
| BMI | 5 | 63962177 | rs1035491 | A | G | BBJ |
| BMI | 5 | 87969927 | rs1846974 | G | A | BBJ |
| BMI | 5 | 122652106 | rs4308481 | C | T | BBJ |
| BMI | 5 | 133861756 | rs329120 | C | T | BBJ |
| BMI | 6 | 32437160 | rs183975233 | T | A | BBJ |
| BMI | 6 | 64705610 | rs148546399 | G | A | BBJ |
| BMI | 6 | 153396875 | rs9397585 | T | C | BBJ |
| BMI | 7 | 69406661 | rs6947395 | A | T | BBJ |
| BMI | 7 | 115368366 | rs143665886 | T | C | BBJ |
| BMI | 8 | 64552779 | rs77636220 | G | A | BBJ |
| BMI | 8 | 95507328 | rs4366055 | A | C | BBJ |
| BMI | 9 | 22129579 | rs7020996 | C | T | BBJ |
| BMI | 9 | 87234111 | rs10868215 | T | C | BBJ |
| BMI | 9 | 97073588 | rs3932549 | A | C | BBJ |
| BMI | 9 | 128137418 | rs5015933 | T | C | BBJ |
| BMI | 10 | 12302607 | rs10795945 | T | C | BBJ |
| BMI | 10 | 18584792 | rs7912454 | A | G | BBJ |
| BMI | 10 | 69834828 | rs80117551 | C | T | BBJ |
| BMI | 10 | 94477539 | rs1832886 | G | A | BBJ |
| BMI | 10 | 99096676 | rs12569457 | C | T | BBJ |
| BMI | 10 | 122897959 | rs1907240 | G | A | BBJ |
| BMI | 10 | 125251751 | rs1568079 | T | A | BBJ |
| BMI | 12 | 31441179 | rs80234489 | A | C | BBJ |
| BMI | 14 | 52511911 | rs75766425 | G | C | BBJ |
| BMI | 14 | 94109502 | rs729050 | G | T | BBJ |
| BMI | 15 | 57541201 | rs2593235 | G | A | BBJ |
| BMI | 15 | 62319432 | rs72749754 | G | C | BBJ |
| BMI | 16 | 73070083 | rs4788694 | C | G | BBJ |
| BMI | 17 | 29036425 | rs180950758 | A | T | BBJ |
| BMI | 17 | 65921834 | rs4790981 | A | G | BBJ |
| BMI | 20 | 18288165 | rs16978956 | A | G | BBJ |
| BMI | 20 | 54145086 | rs2247627 | G | A | BBJ |
| BMI | 20 | 60564086 | rs6089584 | G | C | BBJ |
| BMI | 22 | 40713861 | rs139913 | T | A | BBJ |
| BMI | 1 | 177852580 | rs633715 | T | C | BBJ |
| BMI | 2 | 621558 | rs939584 | C | T | BBJ |
| BMI | 2 | 25158008 | rs713586 | T | C | BBJ |
| BMI | 2 | 58791420 | rs10197655 | G | A | BBJ |
| BMI | 2 | 142615136 | rs12617004 | G | C | BBJ |
| BMI | 2 | 181517996 | rs6433857 | C | T | BBJ |
| BMI | 3 | 52755592 | rs11130319 | A | T | BBJ |
| BMI | 3 | 185524081 | rs4686392 | A | G | BBJ |
| BMI | 4 | 45164637 | rs1996023 | T | G | BBJ |
| BMI | 5 | 74991849 | rs6881648 | A | C | BBJ |
| BMI | 5 | 95867908 | rs10062657 | C | A | BBJ |
| BMI | 5 | 124316031 | rs4357030 | C | T | BBJ |
| BMI | 6 | 20675792 | rs35261542 | C | A | BBJ |
| BMI | 6 | 34179390 | rs6913361 | A | G | BBJ |
| BMI | 6 | 50786008 | rs2206271 | T | A | BBJ |
| BMI | 8 | 76697034 | rs28857569 | T | C | BBJ |
| BMI | 10 | 102425949 | rs2495707 | A | G | BBJ |
| BMI | 10 | 104616663 | rs4409766 | T | C | BBJ |
| BMI | 10 | 114758349 | rs7903146 | C | T | BBJ |
| BMI | 11 | 2857233 | rs60808706 | G | A | BBJ |
| BMI | 11 | 8404501 | rs16937956 | A | G | BBJ |
| BMI | 11 | 27677586 | rs11030100 | G | T | BBJ |
| BMI | 11 | 47761471 | rs11602339 | C | T | BBJ |
| BMI | 12 | 50261809 | rs3205718 | C | T | BBJ |
| BMI | 12 | 112256762 | rs7305242 | T | C | BBJ |
| BMI | 13 | 54107352 | rs9568867 | G | A | BBJ |
| BMI | 16 | 4022694 | rs2540034 | C | T | BBJ |
| BMI | 16 | 20258432 | rs12597682 | C | A | BBJ |
| BMI | 16 | 28538640 | rs62034325 | A | G | BBJ |
| BMI | 16 | 53802494 | rs11642015 | C | T | BBJ |
| BMI | 18 | 40708905 | rs1518170 | T | C | BBJ |
| BMI | 18 | 57829135 | rs6567160 | T | C | BBJ |
| BMI | 19 | 46175046 | rs35560038 | A | T | BBJ |
| BMI | 21 | 40315316 | rs9983113 | G | T | BBJ |
| WHR | 1 | 9335745 | rs6688233 | T | C | CKB |
| WHR | 1 | 23271504 | rs2903995 | A | T | CKB |
| WHR | 1 | 26201164 | rs213624 | A | G | CKB |
| WHR | 1 | 40018509 | rs4660808 | T | C | CKB |
| WHR | 1 | 49928489 | rs946106 | T | C | CKB |
| WHR | 1 | 72835410 | rs2613505 | T | C | CKB |
| WHR | 1 | 86273451 | rs313741 | A | T | CKB |
| WHR | 1 | 91212216 | rs6699397 | A | G | CKB |
| WHR | 1 | 98423149 | rs9659380 | A | G | CKB |
| WHR | 1 | 107573565 | rs2335077 | A | G | CKB |
| WHR | 1 | 114953420 | rs6694768 | T | C | CKB |
| WHR | 1 | 119546842 | rs10923724 | T | C | CKB |
| WHR | 1 | 154814992 | rs6704449 | A | T | CKB |
| WHR | 1 | 154991389 | rs905938 | T | C | CKB |
| WHR | 1 | 170372503 | rs10919388 | A | C | CKB |
| WHR | 1 | 172172467 | rs11589142 | A | C | CKB |
| WHR | 1 | 172331059 | rs1894633 | A | G | CKB |
| WHR | 1 | 177889480 | rs543874 | A | G | CKB |
| WHR | 1 | 200049302 | rs6658424 | A | T | CKB |
| WHR | 1 | 205041542 | rs3903399 | T | C | CKB |
| WHR | 1 | 214173840 | rs3767848 | A | G | CKB |
| WHR | 1 | 219653101 | rs1563355 | T | C | CKB |
| WHR | 1 | 219749261 | rs2494192 | A | G | CKB |
| WHR | 1 | 224051439 | rs6604731 | T | C | CKB |
| WHR | 1 | 243533273 | rs12042959 | A | G | CKB |
| WHR | 2 | 629510 | rs6743060 | A | C | CKB |
| WHR | 2 | 13073967 | rs711869 | A | G | CKB |
| WHR | 2 | 25193998 | rs6749646 | A | T | CKB |
| WHR | 2 | 48962291 | rs17326656 | T | G | CKB |
| WHR | 2 | 58792377 | rs929641 | A | G | CKB |
| WHR | 2 | 59307725 | rs6545714 | A | G | CKB |
| WHR | 2 | 59951465 | rs13028903 | T | C | CKB |
| WHR | 2 | 60170875 | rs2419407 | T | C | CKB |
| WHR | 2 | 66200648 | rs1385167 | A | G | CKB |
| WHR | 2 | 66772000 | rs11897119 | T | C | CKB |
| WHR | 2 | 67478949 | rs4671789 | T | C | CKB |
| WHR | 2 | 67846288 | rs4671193 | T | C | CKB |
| WHR | 2 | 100478648 | rs7558575 | T | C | CKB |
| WHR | 2 | 114517748 | rs4372913 | A | G | CKB |
| WHR | 2 | 119444229 | rs332105 | A | G | CKB |
| WHR | 2 | 161144055 | rs1020731 | A | G | CKB |
| WHR | 2 | 162859436 | rs12621633 | A | G | CKB |
| WHR | 2 | 164877930 | rs399984 | C | G | CKB |
| WHR | 2 | 165667643 | rs409125 | A | T | CKB |
| WHR | 2 | 166162705 | rs12469667 | A | G | CKB |
| WHR | 2 | 181607751 | rs9630986 | C | G | CKB |
| WHR | 2 | 188115398 | rs1569135 | A | G | CKB |
| WHR | 2 | 200775744 | rs1124639 | T | C | CKB |
| WHR | 2 | 213413231 | rs7599312 | A | G | CKB |
| WHR | 2 | 219170525 | rs1017698 | A | G | CKB |
| WHR | 2 | 230739209 | rs17324331 | C | G | CKB |
| WHR | 3 | 9345218 | rs4686340 | A | C | CKB |
| WHR | 3 | 12302462 | rs9878908 | T | C | CKB |
| WHR | 3 | 12690855 | rs6766666 | T | G | CKB |
| WHR | 3 | 15771372 | rs2455848 | T | C | CKB |
| WHR | 3 | 18738940 | rs6550597 | A | G | CKB |
| WHR | 3 | 33872787 | rs12631066 | C | G | CKB |
| WHR | 3 | 35635145 | rs10490869 | A | T | CKB |
| WHR | 3 | 37562141 | rs155524 | A | G | CKB |
| WHR | 3 | 49751585 | rs2291542 | T | C | CKB |
| WHR | 3 | 52516293 | rs6800707 | C | G | CKB |
| WHR | 3 | 53234994 | rs11919522 | A | G | CKB |
| WHR | 3 | 62481063 | rs1452075 | T | C | CKB |
| WHR | 3 | 82704753 | rs6548834 | A | G | CKB |
| WHR | 3 | 85886077 | rs12495178 | T | C | CKB |
| WHR | 3 | 89121921 | rs9942009 | T | C | CKB |
| WHR | 3 | 99525631 | rs793456 | A | G | CKB |
| WHR | 3 | 129341403 | rs6795831 | A | C | CKB |
| WHR | 3 | 131564741 | rs13063979 | T | G | CKB |
| WHR | 3 | 135926622 | rs645040 | T | G | CKB |
| WHR | 3 | 156797648 | rs10049088 | T | C | CKB |
| WHR | 3 | 168949384 | rs12494190 | A | G | CKB |
| WHR | 3 | 171800256 | rs4894803 | A | G | CKB |
| WHR | 3 | 185834290 | rs7647305 | T | C | CKB |
| WHR | 3 | 187632584 | rs12629247 | C | G | CKB |
| WHR | 4 | 965779 | rs11724804 | A | G | CKB |
| WHR | 4 | 3232257 | rs3121419 | T | C | CKB |
| WHR | 4 | 26308792 | rs17644283 | A | G | CKB |
| WHR | 4 | 45175691 | rs13130484 | T | C | CKB |
| WHR | 4 | 56245637 | rs861029 | T | C | CKB |
| WHR | 4 | 89730074 | rs2167750 | T | C | CKB |
| WHR | 4 | 100235053 | rs1789882 | A | G | CKB |
| WHR | 4 | 145868370 | rs789351 | T | C | CKB |
| WHR | 5 | 53302354 | rs2448 | T | C | CKB |
| WHR | 5 | 55816888 | rs455660 | T | C | CKB |
| WHR | 5 | 55860866 | rs3936510 | T | G | CKB |
| WHR | 5 | 75015242 | rs2112347 | T | G | CKB |
| WHR | 5 | 76599022 | rs1382894 | A | T | CKB |
| WHR | 5 | 88001798 | rs2161228 | T | C | CKB |
| WHR | 5 | 103945178 | rs2161097 | T | C | CKB |
| WHR | 5 | 106328326 | rs4395620 | T | C | CKB |
| WHR | 5 | 112542527 | rs1822489 | A | G | CKB |
| WHR | 5 | 118729286 | rs1045241 | T | C | CKB |
| WHR | 5 | 132412299 | rs11747001 | A | G | CKB |
| WHR | 5 | 155824774 | rs4454042 | T | C | CKB |
| WHR | 5 | 158015903 | rs1122080 | A | G | CKB |
| WHR | 5 | 172997978 | rs17738166 | A | G | CKB |
| WHR | 6 | 6765447 | rs4960238 | A | G | CKB |
| WHR | 6 | 7211818 | rs1334576 | A | G | CKB |
| WHR | 6 | 14573063 | rs9296938 | A | G | CKB |
| WHR | 6 | 20581828 | rs7744833 | A | G | CKB |
| WHR | 6 | 31838713 | rs494620 | A | G | CKB |
| WHR | 6 | 40348653 | rs2033529 | A | G | CKB |
| WHR | 6 | 43758873 | rs6905288 | A | G | CKB |
| WHR | 6 | 43810974 | rs9369425 | A | G | CKB |
| WHR | 6 | 50803050 | rs987237 | A | G | CKB |
| WHR | 6 | 50934086 | rs9395645 | A | T | CKB |
| WHR | 6 | 53789830 | rs9370243 | T | G | CKB |
| WHR | 6 | 81346033 | rs1902066 | T | C | CKB |
| WHR | 6 | 85396119 | rs9362083 | A | T | CKB |
| WHR | 6 | 97385975 | rs17448885 | C | G | CKB |
| WHR | 6 | 97946396 | rs10499013 | A | G | CKB |
| WHR | 6 | 98539519 | rs901630 | T | C | CKB |
| WHR | 6 | 100610101 | rs2503099 | A | G | CKB |
| WHR | 6 | 108906200 | rs12206094 | T | C | CKB |
| WHR | 6 | 120213880 | rs2357760 | A | G | CKB |
| WHR | 6 | 127107582 | rs2143679 | A | G | CKB |
| WHR | 6 | 127448249 | rs1936807 | C | G | CKB |
| WHR | 6 | 139829666 | rs605066 | T | C | CKB |
| WHR | 7 | 25862790 | rs2391168 | A | C | CKB |
| WHR | 7 | 25939723 | rs12672425 | T | G | CKB |
| WHR | 7 | 26397239 | rs1534696 | A | C | CKB |
| WHR | 7 | 50750128 | rs2715135 | T | G | CKB |
| WHR | 7 | 68686127 | rs7797307 | C | G | CKB |
| WHR | 7 | 77333267 | rs11764879 | A | G | CKB |
| WHR | 7 | 104756326 | rs1142 | T | C | CKB |
| WHR | 7 | 107614003 | rs4727695 | A | G | CKB |
| WHR | 7 | 112987650 | rs4476935 | T | C | CKB |
| WHR | 7 | 116954785 | rs39312 | A | C | CKB |
| WHR | 7 | 120889272 | rs6942652 | C | G | CKB |
| WHR | 8 | 19824667 | rs15285 | T | C | CKB |
| WHR | 8 | 23610639 | rs9644033 | A | T | CKB |
| WHR | 8 | 25641764 | rs7823561 | A | C | CKB |
| WHR | 8 | 73439070 | rs1431659 | A | G | CKB |
| WHR | 8 | 128334900 | rs13256367 | A | C | CKB |
| WHR | 9 | 15670492 | rs6474945 | T | G | CKB |
| WHR | 9 | 28412375 | rs2183825 | T | C | CKB |
| WHR | 9 | 95568230 | rs1974004 | T | C | CKB |
| WHR | 9 | 96758342 | rs2398893 | A | G | CKB |
| WHR | 9 | 107665978 | rs1800978 | C | G | CKB |
| WHR | 9 | 107887738 | rs9792666 | A | G | CKB |
| WHR | 9 | 111972671 | rs12684047 | A | T | CKB |
| WHR | 9 | 134881443 | rs7025089 | A | C | CKB |
| WHR | 10 | 4963327 | rs12774134 | T | C | CKB |
| WHR | 10 | 5648787 | rs7907173 | A | G | CKB |
| WHR | 10 | 21908803 | rs1243188 | T | C | CKB |
| WHR | 10 | 27904321 | rs1494204 | T | C | CKB |
| WHR | 10 | 32391109 | rs2907794 | A | G | CKB |
| WHR | 10 | 33672884 | rs10827252 | A | G | CKB |
| WHR | 10 | 36227656 | rs708437 | A | G | CKB |
| WHR | 10 | 80907147 | rs780159 | A | G | CKB |
| WHR | 10 | 89604732 | rs10788569 | T | C | CKB |
| WHR | 10 | 94114633 | rs950732 | T | C | CKB |
| WHR | 10 | 95346805 | rs11187537 | C | G | CKB |
| WHR | 10 | 115860058 | rs12777288 | T | C | CKB |
| WHR | 10 | 122875040 | rs17101456 | A | G | CKB |
| WHR | 11 | 8694073 | rs12575252 | C | G | CKB |
| WHR | 11 | 13274553 | rs747601 | A | T | CKB |
| WHR | 11 | 27650524 | rs10742179 | A | G | CKB |
| WHR | 11 | 27695464 | rs11030108 | A | G | CKB |
| WHR | 11 | 30432220 | rs13642 | A | T | CKB |
| WHR | 11 | 43628749 | rs4755720 | T | C | CKB |
| WHR | 11 | 65278461 | rs10896012 | T | C | CKB |
| WHR | 11 | 65894424 | rs3862386 | C | G | CKB |
| WHR | 11 | 111895254 | rs2276390 | T | G | CKB |
| WHR | 11 | 113234679 | rs719802 | T | C | CKB |
| WHR | 11 | 118944675 | rs3825061 | T | C | CKB |
| WHR | 11 | 122014110 | rs579682 | T | C | CKB |
| WHR | 11 | 130271647 | rs747249 | A | G | CKB |
| WHR | 12 | 998365 | rs12828016 | T | G | CKB |
| WHR | 12 | 2055266 | rs7222 | T | C | CKB |
| WHR | 12 | 9075014 | rs1805740 | T | G | CKB |
| WHR | 12 | 12879570 | rs34322 | T | C | CKB |
| WHR | 12 | 14417179 | rs11055887 | A | G | CKB |
| WHR | 12 | 26471364 | rs10842707 | T | C | CKB |
| WHR | 12 | 30865510 | rs10843817 | A | C | CKB |
| WHR | 12 | 33734935 | rs2200155 | A | G | CKB |
| WHR | 12 | 41887940 | rs1458156 | T | C | CKB |
| WHR | 12 | 50247468 | rs7138803 | A | G | CKB |
| WHR | 12 | 54400228 | rs12822416 | A | C | CKB |
| WHR | 12 | 54421476 | rs10876528 | A | C | CKB |
| WHR | 12 | 66441684 | rs11176015 | T | C | CKB |
| WHR | 12 | 89771903 | rs704061 | T | C | CKB |
| WHR | 12 | 94092690 | rs10745659 | C | G | CKB |
| WHR | 12 | 98772975 | rs7311622 | T | C | CKB |
| WHR | 12 | 108594069 | rs4964656 | C | G | CKB |
| WHR | 12 | 124446728 | rs7312404 | A | G | CKB |
| WHR | 12 | 124505444 | rs863750 | T | C | CKB |
| WHR | 13 | 31033232 | rs1045411 | T | C | CKB |
| WHR | 13 | 51200451 | rs637310 | A | G | CKB |
| WHR | 13 | 51221618 | rs797486 | A | C | CKB |
| WHR | 13 | 93896935 | rs12430764 | A | G | CKB |
| WHR | 13 | 111040798 | rs9515201 | A | C | CKB |
| WHR | 13 | 112225701 | rs1163627 | A | C | CKB |
| WHR | 14 | 25928179 | rs10132280 | A | C | CKB |
| WHR | 14 | 58815839 | rs1190982 | T | C | CKB |
| WHR | 14 | 65421274 | rs2898885 | T | C | CKB |
| WHR | 14 | 91547136 | rs7492628 | C | G | CKB |
| WHR | 14 | 103350197 | rs12590238 | C | G | CKB |
| WHR | 15 | 31705683 | rs4779526 | A | T | CKB |
| WHR | 15 | 40990353 | rs2928140 | C | G | CKB |
| WHR | 15 | 42102285 | rs12440605 | A | G | CKB |
| WHR | 15 | 51748610 | rs3736485 | A | G | CKB |
| WHR | 15 | 53044002 | rs10851523 | C | G | CKB |
| WHR | 15 | 56781255 | rs16976932 | A | G | CKB |
| WHR | 15 | 62435156 | rs12440695 | T | C | CKB |
| WHR | 15 | 67033151 | rs1440372 | T | C | CKB |
| WHR | 15 | 67661784 | rs8043060 | A | G | CKB |
| WHR | 15 | 73618309 | rs2660824 | T | C | CKB |
| WHR | 15 | 75069282 | rs936226 | T | C | CKB |
| WHR | 15 | 81058640 | rs12593088 | A | G | CKB |
| WHR | 15 | 92570921 | rs12101393 | C | G | CKB |
| WHR | 15 | 94023132 | rs8024294 | A | G | CKB |
| WHR | 16 | 4908956 | rs8060576 | T | C | CKB |
| WHR | 16 | 24806420 | rs7186893 | T | G | CKB |
| WHR | 16 | 28883241 | rs7498665 | A | G | CKB |
| WHR | 16 | 29995218 | rs4788204 | A | G | CKB |
| WHR | 16 | 49864791 | rs2047937 | T | C | CKB |
| WHR | 16 | 53801985 | rs9923544 | T | C | CKB |
| WHR | 16 | 53865975 | rs9302652 | T | C | CKB |
| WHR | 16 | 69556715 | rs889398 | T | C | CKB |
| WHR | 16 | 81534790 | rs2925979 | T | C | CKB |
| WHR | 16 | 82872628 | rs7206608 | C | G | CKB |
| WHR | 16 | 85258191 | rs7198287 | T | C | CKB |
| WHR | 17 | 2136065 | rs7217226 | T | G | CKB |
| WHR | 17 | 3981066 | rs8070737 | T | G | CKB |
| WHR | 17 | 17493272 | rs4646342 | A | G | CKB |
| WHR | 17 | 21279289 | rs7213608 | T | C | CKB |
| WHR | 17 | 34848874 | rs2306589 | T | C | CKB |
| WHR | 17 | 46080233 | rs8071778 | C | G | CKB |
| WHR | 17 | 59492714 | rs758598 | A | G | CKB |
| WHR | 17 | 61728881 | rs8075273 | A | C | CKB |
| WHR | 17 | 65947640 | rs12449442 | A | G | CKB |
| WHR | 17 | 68446861 | rs11654387 | C | G | CKB |
| WHR | 17 | 73230856 | rs9988 | T | C | CKB |
| WHR | 17 | 79923718 | rs4239275 | T | C | CKB |
| WHR | 18 | 13072979 | rs1787013 | T | C | CKB |
| WHR | 18 | 34690744 | rs10164099 | T | C | CKB |
| WHR | 18 | 40736590 | rs1158805 | A | C | CKB |
| WHR | 18 | 46853270 | rs494752 | C | G | CKB |
| WHR | 18 | 57829135 | rs6567160 | T | C | CKB |
| WHR | 18 | 57960769 | rs17773412 | T | C | CKB |
| WHR | 18 | 58039276 | rs2229616 | T | C | CKB |
| WHR | 19 | 2176586 | rs12459350 | A | G | CKB |
| WHR | 19 | 18244690 | rs885683 | A | G | CKB |
| WHR | 19 | 18389135 | rs12608504 | A | G | CKB |
| WHR | 19 | 30286822 | rs17513613 | T | C | CKB |
| WHR | 19 | 33880000 | rs3786893 | C | G | CKB |
| WHR | 19 | 33893008 | rs3786897 | A | G | CKB |
| WHR | 19 | 46181392 | rs1800437 | C | G | CKB |
| WHR | 20 | 6623374 | rs979012 | T | C | CKB |
| WHR | 20 | 33905619 | rs1406948 | A | G | CKB |
| WHR | 20 | 39690342 | rs1997833 | T | C | CKB |
| WHR | 20 | 42010996 | rs6130360 | A | G | CKB |
| WHR | 20 | 45529571 | rs2236519 | A | G | CKB |
| WHR | 20 | 45789953 | rs3092781 | T | C | CKB |
| WHR | 20 | 50982870 | rs6021889 | A | G | CKB |
| WHR | 20 | 56135199 | rs1328757 | T | C | CKB |
| WHR | 21 | 39484323 | rs9976841 | A | G | CKB |
| WHR | 22 | 29318299 | rs9625645 | A | G | CKB |
| WHR | 22 | 29449477 | rs2294239 | A | G | CKB |
| WHR | 22 | 40669648 | rs733381 | A | G | CKB |
| WHR | 22 | 47214749 | rs8141715 | T | G | CKB |

BBJ = BioBank Japan

Table S2. Results of the 2 sample Mendelian Randomisation analysis for the five meta-analyses from the recent EAS GWAS removing SNPs with a minor allele frequency (MAF) <0.45 and >0.55.

|  |  |  |  |  |  | **BMI** |  |  |  | |  |
| --- | --- | --- | --- | --- | --- | --- | --- | --- | --- | --- | --- |
| **Trait** | **OR IVW (95%CI)** | **P_IVW_** | **OR Egger (95%CI)** | **P_Egger_** | **P_EggerIntercept_** | **OR WM (95%CI)** | **P_WM_** | **OR PWM (95%CI)** | **P _PWM_** | **N SNPS** | |
| Broad discovery analysis | 0.974 (0.955, 0.994) | ***0.010*** | 0.985 (0.942, 1.030) | 0.512 | 0.576 | 0.981 (0.950, 1.013) | 0.239 | 0.981 (0.950, 1.013) | 0.244 | 721 | |
| Clinical depression analysis | 0.955 (0.928, 0.983) | ***2.11E-03*** | 0.985 (0.923, 1.050) | 0.635 | 0.298 | 0.971 (0.926, 1.019) | 0.232 | 0.979 (0.934, 1.026) | 0.367 | 690 | |
| Symptom based analysis | 1.003 (0.970, 1.036) | 0.878 | 0.983 (0.912, 1.060) | 0.658 | 0.570 | 0.989 (0.938, 1.043) | 0.683 | 0.985 (0.935, 1.038) | 0.573 | 643 | |
| Resident in East Asia | 0.964 (0.937, 0.992) | ***0.013*** | 0.955 (0.896, 1.018) | 0.160 | 0.755 | 0.994 (0.950, 1.041) | 0.806 | 0.996 (0.951, 1.044) | 0.869 | 704 | |
| Resident in UK or USA | 0.997 (0.969, 1.026) | 0.831 | 1.055 (0.990, 1.125) | 0.101 | 0.053 | 1.007 (0.961, 1.055) | 0.766 | 1.006 (0.961, 1.053) | 0.796 | 716 | |
|  |  |  |  |  |  | **WHR** |  |  |  |  | |
| **Trait** | **OR IVW (95%CI)** | **P_IVW_** | **OR Egger (95%CI)** | **P_Egger_** | **P_EggerIntercept_** | **OR WM (95%CI)** | **P_WM_** | **OR PWM (95%CI)** | **P _PWM_** | **N SNPS** | |
| Broad discovery analysis | 0.970 (0.935, 1.007) | 0.117 | 0.962 (0.911, 1.014) | 0.152 | 0.639 | 0.983 (0.928, 1.041) | 0.559 | 0.984 (0.934, 1.037) | 0.551 | 236 | |
| Clinical depression analysis | 0.947 (0.899, 0.997) | **0.039** | 0.937 (0.871, 1.008) | 0.082 | 0.687 | 0.984 (0.906, 1.070) | 0.708 | 0.985 (0.909, 1.067) | 0.707 | 231 | |
| Symptom based analysis | 0.978 (0.922, 1.037) | 0.459 | 0.962 (0.882, 1.049) | 0.377 | 0.604 | 1.006 (0.914, 1.108) | 0.901 | 1.007 (0.918, 1.105) | 0.882 | 219 | |
| Resident in East Asia | 0.946 (0.897, 0.997) | **0.041** | 0.933 (0.864, 1.006) | 0.073 | 0.613 | 0.995 (0.919, 1.079) | 0.911 | 0.996 (0.918, 1.081) | 0.927 | 232 | |
| Resident in UK or USA | 1.010 (0.958, 1.066) | 0.709 | 1.008 (0.933, 1.089) | 0.837 | 0.941 | 1.011 (0.931, 1.098) | 0.793 | 0.999 (0.919, 1.087) | 0.986 | 236 | |

The IVW estimates assumes that there is no horizontal pleiotropy. Egger uses a weighted regression with an unconstrained intercept to remove the assumption that all genetic variants are valid instrumental variables and is therefore less susceptible to confounding from potentially pleiotropic variants. The Median-MR method takes the median instrumental variable from all variants included and is robust when up to 50% of the genetic variants are invalid. If all methods are broadly consistent this strengthens our causal inference. Bold p values reach P<0.05 significance and bold italicised p values reach the multiple testing threshold P<0.025 (see methods).

Table S3. Results of the 2 sample Mendelian Randomisation analysis for the five meta-analyses from the recent EAS GWAS removing SNPs with a different trait raising allele between Europeans and East Asians.

|  |  |  |  |  |  | **BMI** |  |  |  |  |
| --- | --- | --- | --- | --- | --- | --- | --- | --- | --- | --- |
| **Trait** | **OR IVW (95%CI)** | **P_IVW_** | **OR Egger (95%CI)** | **P_Egger_** | **P_EggerIntercept_** | **OR WM (95%CI)** | **P_WM_** | **OR PWM (95%CI)** | **P _PWM_** | **N SNPS** |
| Broad discovery analysis | 0.983 (0.964, 1.002) | 0.080 | 0.967 (0.925, 1.011) | 0.137 | 0.425 | 0.981 (0.949, 1.014) | 0.262 | 0.981 (0.950, 1.013) | 0.248 | 647 |
| Clinical depression analysis | 0.962 (0.934, 0.992) | ***0.012*** | 0.973 (0.910, 1.041) | 0.429 | 0.711 | 0.974 (0.930, 1.021) | 0.275 | 0.978 (0.933, 1.026) | 0.362 | 622 |
| Symptom based analysis | 0.997 (0.965, 1.030) | 0.861 | 0.964 (0.895, 1.038) | 0.336 | 0.325 | 0.984 (0.931, 1.040) | 0.563 | 0.984 (0.932, 1.038) | 0.550 | 590 |
| Resident in East Asia | 0.969 (0.942, 0.998) | **0.035** | 0.942 (0.883, 1.005) | 0.072 | 0.334 | 0.973 (0.928, 1.021) | 0.271 | 0.974 (0.928, 1.022) | 0.288 | 634 |
| Resident in UK or USA | 1.010 (0.981, 1.039) | 0.499 | 1.023 (0.958, 1.091) | 0.498 | 0.671 | 1.008 (0.962, 1.056) | 0.748 | 1.007 (0.961, 1.055) | 0.767 | 643 |
|  |  |  |  |  |  | **WHR** |  |  |  |  |
| **Trait** | **OR IVW (95%CI)** | **P_IVW_** | **OR Egger (95%CI)** | **P_Egger_** | **P_EggerIntercept_** | **OR WM (95%CI)** | **P_WM_** | **OR PWM (95%CI)** | **P _PWM_** | **N SNPS** |
| Broad discovery analysis | 0.971 (0.932, 1.012) | 0.172 | 0.974 (0.917, 1.035) | 0.403 | 0.889 | 0.988 (0.933, 1.046) | 0.670 | 1.002 (0.945, 1.061) | 0.957 | 175 |
| Clinical depression analysis | 0.959 (0.908, 1.013) | 0.140 | 0.980 (0.905, 1.062) | 0.621 | 0.472 | 0.989 (0.909, 1.076) | 0.799 | 0.989 (0.907, 1.079) | 0.807 | 171 |
| Symptom based analysis | 0.965 (0.908, 1.025) | 0.247 | 0.973 (0.889, 1.066) | 0.562 | 0.790 | 1.006 (0.915, 1.106) | 0.896 | 1.007 (0.913, 1.110) | 0.891 | 160 |
| Resident in East Asia | 0.934 (0.881, 0.990) | ***0.023*** | 0.962 (0.884, 1.048) | 0.375 | 0.350 | 0.996 (0.915, 1.084) | 0.927 | 0.997 (0.915, 1.086) | 0.942 | 172 |
| Resident in UK or USA | 1.034 (0.978, 1.094) | 0.240 | 0.989 (0.911, 1.073) | 0.789 | 0.144 | 1.033 (0.945, 1.128) | 0.477 | 1.022 (0.943, 1.109) | 0.595 | 175 |

The IVW estimates assumes that there is no horizontal pleiotropy. Egger uses a weighted regression with an unconstrained intercept to remove the assumption that all genetic variants are valid instrumental variables and is therefore less susceptible to confounding from potentially pleiotropic variants. The Median-MR method takes the median instrumental variable from all variants included and is robust when up to 50% of the genetic variants are invalid. If all methods are broadly consistent this strengthens our causal inference. Bold p values reach P<0.05 significance and bold italicised p values reach the multiple testing threshold P<0.025 (see methods).

Table S4. Results of the 2 sample Mendelian Randomisation analysis for the five meta-analyses from the recent EAS GWAS removing the SNP in MC4R.

| **BMI** | | | | | | | | | | |
| --- | --- | --- | --- | --- | --- | --- | --- | --- | --- | --- |
| **Strata** | **Trait** | **OR IVW (95%CI)** | **P_IVW_** | **OR Egger (95%CI)** | **P_Egger_** | **P_EggerIntercept_** | **OR WM (95%CI)** | **P_WM_** | **OR PWM (95%CI)** | **P _PWM_** |
| Without MC4R | Broad discovery analysis | 0.978 (0.960, 0.996) | ***0.018*** | 0.984 (0.946, 1.024) | 0.432 | 0.730 | 0.983 (0.952, 1.014) | 0.281 | 0.983 (0.953, 1.014) | 0.273 |
| Without MC4R | Clinical depression analysis | 0.958 (0.933, 0.984) | ***2.04E-03*** | 0.985 (0.929, 1.044) | 0.605 | 0.295 | 0.977 (0.934, 1.022) | 0.309 | 0.979 (0.938, 1.023) | 0.353 |
| Without MC4R | Symptom based analysis | 1.006 (0.976, 1.032) | 0.696 | 0.981 (0.920, 1.046) | 0.564 | 0.393 | 0.988 (0.940, 1.038) | 0.619 | 0.985 (0.937, 1.035) | 0.550 |
| Without MC4R | Resident in East Asia | 0.968 (0.943, 0.998) | ***0.018*** | 0.966 (0.912, 1.023) | 0.239 | 0.932 | 0.993 (0.951, 1.038) | 0.762 | 0.994 (0.952, 1.038) | 0.795 |
| Without MC4R | Resident in UK or USA | 1.001 (0.975, 1.027) | 0.964 | 1.024 (0.968, 1.083) | 0.412 | 0.367 | 0.991 (0.948, 1.037) | 0.708 | 0.990 (0.947, 1.036) | 0.671 |
| **WHR** | | | | | | | | | | |
| **Strata** | **Trait** | **OR IVW (95%CI)** | **P_IVW_** | **OR Egger (95%CI)** | **P_Egger_** | **P_EggerIntercept_** | **OR WM (95%CI)** | **P_WM_** | **OR PWM (95%CI)** | **P _PWM_** |
| Without MC4R | Broad discovery analysis | 0.977 (0.941, 1.015) | 0.242 | 0.977 (0.924, 1.032) | 0.400 | 0.963 | 1.009 (0.955, 1.066) | 0.742 | 1.014 (0.957, 1.074) | 0.643 |
| Without MC4R | Clinical depression analysis | 0.957 (0.909, 1.008) | 0.095 | 0.960 (0.891, 1.034) | 0.284 | 0.905 | 0.989 (0.910, 1.075) | 0.796 | 0.989 (0.904, 1.082) | 0.812 |
| Without MC4R | Symptom based analysis | 0.981 (0.926, 1.040) | 0.525 | 0.969 (0.890, 1.055) | 0.466 | 0.686 | 1.010 (0.920, 1.109) | 0.838 | 1.011 (0.922, 1.109) | 0.819 |
| Without MC4R | Resident in East Asia | 0.950 (0.900, 1.003) | 0.064 | 0.960 (0.888, 1.038) | 0.305 | 0.724 | 0.999 (0.922, 1.082) | 0.973 | 0.999 (0.925, 1.080) | 0.990 |
| Without MC4R | Resident in UK or USA | 1.023 (0.970, 1.078) | 0.398 | 1.002 (0.927, 1.082) | 0.967 | 0.459 | 1.014 (0.937, 1.098) | 0.723 | 0.999 (0.920, 1.085) | 0.985 |

The IVW estimates assumes that there is no horizontal pleiotropy. Egger uses a weighted regression with an unconstrained intercept to remove the assumption that all genetic variants are valid instrumental variables and is therefore less susceptible to confounding from potentially pleiotropic variants. The Median-MR method takes the median instrumental variable from all variants included and is robust when up to 50% of the genetic variants are invalid. If all methods are broadly consistent this strengthens our causal inference. Bold p values reach P<0.05 significance and bold italicised p values reach the multiple testing threshold P<0.025 (see methods)

Table S5: The association between BMI/WHR GRS and BMI/WHR by sex and by region in the China Kadoorie Biobank. (Bold P values reached GWS.)

| Region | Urban_rural | Outcome | Exposure | Sex | beta | se | P |
| --- | --- | --- | --- | --- | --- | --- | --- |
| Qingdao | Urban | BMI | BMI GRS | Male | 0.573 | 0.063 | **8.40E-20** |
| Qingdao | Urban | BMI | BMI GRS | Female | 0.524 | 0.051 | **8.20E-25** |
| Harbin | Urban | BMI | BMI GRS | Male | 0.527 | 0.052 | **6.70E-24** |
| Harbin | Urban | BMI | BMI GRS | Female | 0.611 | 0.042 | **7.60E-48** |
| Haikou | Urban | BMI | BMI GRS | Male | 0.476 | 0.089 | 1.10E-07 |
| Haikou | Urban | BMI | BMI GRS | Female | 0.645 | 0.067 | **1.70E-21** |
| Suzhou | Urban | BMI | BMI GRS | Male | 0.394 | 0.064 | **8.00E-10** |
| Suzhou | Urban | BMI | BMI GRS | Female | 0.730 | 0.055 | **2.10E-39** |
| Liuzhou | Urban | BMI | BMI GRS | Male | 0.346 | 0.065 | 1.00E-07 |
| Liuzhou | Urban | BMI | BMI GRS | Female | 0.510 | 0.055 | **2.40E-20** |
| Sichuan | Rural | BMI | BMI GRS | Male | 0.399 | 0.053 | **3.80E-14** |
| Sichuan | Rural | BMI | BMI GRS | Female | 0.629 | 0.047 | **4.30E-41** |
| Gansu | Rural | BMI | BMI GRS | Male | 0.445 | 0.055 | **1.30E-15** |
| Gansu | Rural | BMI | BMI GRS | Female | 0.627 | 0.046 | **2.50E-42** |
| Henan | Rural | BMI | BMI GRS | Male | 0.485 | 0.050 | **2.20E-22** |
| Henan | Rural | BMI | BMI GRS | Female | 0.625 | 0.046 | **2.00E-41** |
| Zhejiang | Rural | BMI | BMI GRS | Male | 0.590 | 0.051 | **3.20E-30** |
| Zhejiang | Rural | BMI | BMI GRS | Female | 0.687 | 0.044 | **1.50E-54** |
| Hunan | Rural | BMI | BMI GRS | Male | 0.520 | 0.049 | **8.70E-26** |
| Hunan | Rural | BMI | BMI GRS | Female | 0.658 | 0.044 | **4.40E-49** |
| Qingdao | Urban | WHR | WHR GRS | Male | 0.247 | 0.111 | 0.03 |
| Qingdao | Urban | WHR | WHR GRS | Female | 0.597 | 0.091 | **6.40E-11** |
| Harbin | Urban | WHR | WHR GRS | Male | 0.589 | 0.095 | **5.80E-10** |
| Harbin | Urban | WHR | WHR GRS | Female | 0.547 | 0.068 | **1.30E-15** |
| Haikou | Urban | WHR | WHR GRS | Male | 0.398 | 0.139 | 4.30E-03 |
| Haikou | Urban | WHR | WHR GRS | Female | 0.517 | 0.103 | 6.20E-07 |
| Suzhou | Urban | WHR | WHR GRS | Male | 0.424 | 0.126 | 7.40E-04 |
| Suzhou | Urban | WHR | WHR GRS | Female | 0.402 | 0.093 | 1.70E-05 |
| Liuzhou | Urban | WHR | WHR GRS | Male | 0.437 | 0.124 | 4.30E-04 |
| Liuzhou | Urban | WHR | WHR GRS | Female | 0.529 | 0.107 | 8.60E-07 |
| Sichuan | Rural | WHR | WHR GRS | Male | 0.483 | 0.098 | 8.20E-07 |
| Sichuan | Rural | WHR | WHR GRS | Female | 0.579 | 0.075 | **1.30E-14** |
| Gansu | Rural | WHR | WHR GRS | Male | 0.417 | 0.100 | 3.30E-05 |
| Gansu | Rural | WHR | WHR GRS | Female | 0.700 | 0.081 | **1.10E-17** |
| Henan | Rural | WHR | WHR GRS | Male | 0.422 | 0.091 | 3.30E-06 |
| Henan | Rural | WHR | WHR GRS | Female | 0.568 | 0.070 | **7.40E-16** |
| Zhejiang | Rural | WHR | WHR GRS | Male | 0.538 | 0.107 | 4.90E-07 |
| Zhejiang | Rural | WHR | WHR GRS | Female | 0.668 | 0.077 | **7.20E-18** |
| Hunan | Rural | WHR | WHR GRS | Male | 0.356 | 0.090 | 7.40E-05 |
| Hunan | Rural | WHR | WHR GRS | Female | 0.593 | 0.070 | **2.80E-17** |

Table S6:The observational associations between body composition and depression in individuals with valid genetic data in the China Kadoorie Biobank further adjusted for measures of socioeconomic status .

| **BMI** | | | | | | | | | | | | |
| --- | --- | --- | --- | --- | --- | --- | --- | --- | --- | --- | --- | --- |
|  |  | **All** | | |  | **Urban** | | |  | **Rural** | | |
| **Mental health outcome** | **Strata** | **N cases (controls)** | **OR (95% CI) per SD higher BMI** | **P^a^** |  | **N cases (controls)** | **OR (95% CI) per SD higher BMI** | **P^a^** |  | **N cases (controls)** | **OR (95% CI) per SD higher BMI** | **P^a^** |
| Depressive symptoms | All | 3,398 (96,979) | 0.90 (0.87, 0.93) | 1.00E-08 |  | 1,080 (42,800) | 0.89 (0.84, 0.95) | 3.60E-04 |  | 2,318 (54,179) | 0.92 (0.88, 0.96) | 8.90E-05 |
|  | Male | 1,170 (41,764) | 0.88 (0.82, 0.93) | 3.80E-05 |  | 326 (17,639) | 0.90 (0.81, 1.01) | 0.084 |  | 844 (24,125) | 0.87 (0.81, 0.94) | 3.90E-04 |
|  | Female | 2,228 (55,215) | 0.91 (0.87, 0.95) | 2.80E-05 |  | 754 (25,161) | 0.88 (0.82, 0.95) | 1.30E-03 |  | 1,474 (30,054) | 0.94 (0.89, 0.99) | 0.017 |
| Major depression | All | 760 (96,979) | 0.86 (0.80, 0.93) | 8.50E-05 |  | 281 (42,800) | 0.92 (0.81, 1.03) | 0.150 |  | 479 (54,179) | 0.84 (0.77, 0.93) | 3.70E-04 |
|  | Male | 246 (41,764) | 0.80 (0.70, 0.92) | 1.40E-03 |  | 79 (17,639) | 1.07 (0.85, 1.34) | 0.570 |  | 167 (24,125) | 0.69 (0.59, 0.82) | 2.20E-05 |
|  | Female | 514 (55,215) | 0.89 (0.81, 0.97) | 0.008 |  | 202 (25,161) | 0.87 (0.75, 1.00) | 0.053 |  | 312 (30,054) | 0.91 (0.82, 1.02) | 0.110 |
| **WHR** | | | | | | | | | | | | |
| Depressive symptoms | All | 3,398 (96,979) | 0.90 (0.86, 0.93) | 2.00E-09 |  | 1,080 (42,800) | 0.87 (0.82, 0.93) | 5.60E-05 |  | 2,318 (54,179) | 0.93 (0.89, 0.97) | 1.30E-03 |
|  | Male | 1,170 (41,764) | 0.89 (0.84, 0.94) | 7.60E-05 |  | 326 (17,639) | 0.88 (0.79, 0.99) | 0.029 |  | 844 (24,125) | 0.90 (0.84, 0.97) | 3.10E-03 |
|  | Female | 2,228 (55,215) | 0.89 (0.85, 0.93) | 6.50E-07 |  | 754 (25,161) | 0.86 (0.80, 0.93) | 2.40E-04 |  | 1,474 (30,054) | 0.94 (0.89, 0.99) | 0.029 |
| Major depression | All | 760 (96,979) | 0.88 (0.81, 0.95) | 6.00E-04 |  | 281 (42,800) | 0.91 (0.80, 1.03) | 0.140 |  | 479 (54,179) | 0.91 (0.82, 1.00) | 0.045 |
|  | Male | 246 (41,764) | 0.83 (0.73, 0.94) | 3.40E-03 |  | 79 (17,639) | 1.03 (0.82, 1.30) | 0.770 |  | 167 (24,125) | 0.77 (0.66, 0.91) | 1.30E-03 |
|  | Female | 514 (55,215) | 0.90 (0.82, 0.98) | 0.021 |  | 202 (25,161) | 0.87 (0.75, 1.01) | 0.073 |  | 312 (30,054) | 0.97 (0.87, 1.10) | 0.660 |

*Pa adjusted for age, sex, region, smoking status, household income, education and SES determined by a principal component analysis of six related SES measures (see methods)*

Table S7: The observational associations between higher BMI and depression when individuals were classified as having a normal BMI (18.5≤BMI≤23.9 kg/m2), overweight (23.9<BMI<28) or obese (BMI≥28kg/m2).

| **Mental health outcome** | **Strata** | **Region** | **N cases (controls)** | **OR (95% CI) of depressive symptoms in overweight versus normal weight individuals** | **P^a^** | **OR (95% CI) of depressive symptoms in obese versus normal weight individuals** | **P^a^** |
| --- | --- | --- | --- | --- | --- | --- | --- |
| Depressive symptoms | All | Both | 2,157 (59,979) | 0.87 (0.80, 0.94) | 5.70E-04 | 0.84 (0.74, 0.95) | 6.90E-03 |
|  | Male | Both | 741 (26,061) | 0.87 (0.76, 1.00) | 0.04 | 0.74 (0.58, 0.95) | 0.02 |
|  | Female | Both | 1,416 (33,918) | 0.86 (0.78, 0.95) | 2.40E-03 | 0.86 (0.75, 1.00) | 0.05 |
|  | All | Urban only | 628 (24,747) | 0.90 (0.78, 1.03) | 0.11 | 0.80 (0.66, 0.98) | 0.03 |
|  | Male | Urban only | 178 (9,786) | 0.90 (0.71, 1.15) | 0.41 | 0.64 (0.43, 0.94) | 0.02 |
|  | Female | Urban only | 450 (14,961) | 0.88 (0.74, 1.03) | 0.12 | 0.84 (0.67, 1.07) | 0.16 |
|  | All | Rural only | 1,529 (35,232) | 0.87 (0.79, 0.96) | 6.40E-03 | 0.89 (0.75, 1.04) | 0.14 |
|  | Male | Rural only | 563 (16,275) | 0.89 (0.75, 1.06) | 0.18 | 0.87 (0.63, 1.20) | 0.40 |
|  | Female | Rural only | 966 (18,957) | 0.86 (0.76, 0.97) | 0.01 | 0.88 (0.73, 1.06) | 0.19 |
| Major depression | All | Both | 456 (59,979) | 0.94 (0.80, 1.11) | 0.47 | 0.89 (0.69, 1.15) | 0.37 |
|  | Male | Both | 148 (26,061) | 0.89 (0.66, 1.20) | 0.44 | 0.85 (0.51, 1.42) | 0.54 |
|  | Female | Both | 308 (33,918) | 0.95 (0.78, 1.16) | 0.61 | 0.87 (0.64, 1.18) | 0.36 |
|  | All | Urban only | 155 (24,747) | 1.01 (0.77, 1.32) | 0.93 | 0.97 (0.67, 1.41) | 0.88 |
|  | Male | Urban only | 44 (9,786) | 0.94 (0.57, 1.55) | 0.81 | 1.00 (0.51, 1.98) | 0.99 |
|  | Female | Urban only | 111 (14,961) | 1.02 (0.74, 1.41) | 0.89 | 0.93 (0.59, 1.47) | 0.77 |
|  | All | Rural only | 301 (35,232) | 0.91 (0.73, 1.12) | 0.37 | 0.80 (0.55, 1.16) | 0.24 |
|  | Male | Rural only | 104 (16,274) | 0.87 (0.59, 1.27) | 0.47 | 0.63 (0.28, 1.45) | 0.28 |
|  | Female | Rural only | 197 (18,957) | 0.91 (0.71, 1.18) | 0.49 | 0.83 (0.55, 1.27) | 0.39 |

*P^a^ adjusted for age, region and sex*

Table S8. Results of the 2 sample Mendelian Randomisation analysis for the five meta-analyses from the recent EAS GWAS using the inverse-variance weighted method.

|  | **BMI** |  |  | **WHR** |  |  | |
| --- | --- | --- | --- | --- | --- | --- | --- |
| **Trait** | **OR IVW (95%CI)** | **P_IVW_** | **N SNPS** | **OR IVW (95%CI)** | **P_IVW_** | **N SNPS** | |
| Broad discovery analysis | 0.977 (0.960, 0.995) | ***0.014*** | 834 | 0.975 (0.939, 1.012) | 0.176 | 263 | |
| Clinical depression analysis | 0.957 (0.932, 0.983) | ***1.44E-03*** | 799 | 0.953 (0.906, 1.002) | 0.063 | 258 | |
| Symptom based analysis | 1.006 (0.976, 1.036) | 0.696 | 751 | 0.981 (0.926, 1.040) | 0.525 | 246 | |
| Resident in East Asia | 0.967 (0.941, 0.992) | ***0.012*** | 815 | 0.945 (0.897, 0.996) | **0.036** | 259 | |
| Resident in UK or USA | 1.001 (0.975, 1.027) | 0.943 | 829 | 1.024 (0.972, 1.078) | 0.375 | 263 | |
| *Data are presented as Odds Ratio (95% Confidence Intervals).* | | | | | | |  |

We present the 2-sample MR IVW estimates which assumes that there is no horizontal pleiotropy (under a fixed effect model) or, if implemented under a random effects model after detecting heterogeneity among the causal estimates, that: the strength of the association of the genetic instruments with the risk factor is not correlated with the magnitude of the pleiotropic effects; and the pleiotropic effects have an average value of zero.

Bold p values reach P<0.05 significance and bold italicised p values reach the multiple testing threshold P<0.025 (see methods)*.*

Table S9. Results of the 2 sample Mendelian Randomisation analysis for the five meta-analyses from the recent EAS GWAS using pleiotropy resistant methods: Egger, weighted median(WM) and penalised weighted median (PWM). Data are presented as Odds Ratio (95% confidence interval).

|  |  |  |  |  | **BMI** |  |  |
| --- | --- | --- | --- | --- | --- | --- | --- |
| **Trait** | **OR Egger (95%CI)** | **P_Egger_** | **P_EggerIntercept_** | **OR WM (95%CI)** | **P_WM_** | **OR PWM (95%CI)** | **P _PWM_** |
| Broad discovery analysis | 0.981 (0.943, 1.020) | 0.329 | 0.840 | 0.982 (0.951, 1.013) | 0.241 | 0.981 (0.952, 1.012) | 0.236 |
| Clinical depression analysis | 0.979 (0.925, 1.037) | 0.472 | 0.368 | 0.971 (0.930, 1.014) | 0.181 | 0.974 (0.932, 1.019) | 0.252 |
| Symptom based analysis | 0.981 (0.920, 1.046) | 0.564 | 0.393 | 0.988 (0.942, 1.035) | 0.601 | 0.985 (0.939, 1.033) | 0.533 |
| Resident in East Asia | 0.960 (0.907, 1.015) | 0.150 | 0.774 | 0.980 (0.937, 1.025) | 0.378 | 0.985 (0.944, 1.027) | 0.475 |
| Resident in UK or USA | 1.025 (0.969, 1.083) | 0.389 | 0.349 | 0.993 (0.951, 1.037) | 0.756 | 0.992 (0.950, 1.035) | 0.704 |
|  |  |  |  |  | **WHR** |  |  |
| **Trait** | **OR Egger (95%CI)** | **P_Egger_** | **P_EggerIntercept_** | **OR WM (95%CI)** | **P_WM_** | **OR PWM (95%CI)** | **P _PWM_** |
| Broad discovery analysis | 0.971 (0.921, 1.024) | 0.283 | 0.859 | 0.985 (0.933, 1.041) | 0.602 | 1.001 (0.947, 1.057) | 0.982 |
| Clinical depression analysis | 0.953 (0.887, 1.024) | 0.189 | 0.993 | 0.989 (0.911, 1.073) | 0.790 | 0.989 (0.913, 1.071) | 0.786 |
| Symptom based analysis | 0.969 (0.890, 1.055) | 0.466 | 0.686 | 1.010 (0.917, 1.112) | 0.843 | 1.011 (0.921, 1.109) | 0.820 |
| Resident in East Asia | 0.950 (0.881, 1.024) | 0.179 | 0.857 | 0.996 (0.918, 1.080) | 0.915 | 0.996 (0.921, 1.078) | 0.930 |
| Resident in UK or USA | 1.004 (0.932, 1.082) | 0.917 | 0.477 | 1.030 (0.954, 1.112) | 0.456 | 1.018 (0.939, 1.103) | 0.670 |

The IVW estimates assumes that there is no horizontal pleiotropy. Egger uses a weighted regression with an unconstrained intercept to remove the assumption that all genetic variants are valid instrumental variables and is therefore less susceptible to confounding from potentially pleiotropic variants. The Median-MR method takes the median instrumental variable from all variants included and is robust when up to 50% of the genetic variants are invalid. If all methods are broadly consistent this strengthens our causal inference.

Bold p values reach P<0.05 significance and bold italicised p values reach the multiple testing threshold P<0.025 (see methods)

Table S10: The meta-analysed estimates with heterogeneity statistics for genetic 1-sample associations between BMI/WHR and depressive symptoms/MDE in individuals with valid genetic data from the China Kadoorie Biobank.

| **Exposure** | **Outcome** | **Sex** | **Region** | **OR (95% CI)** | **P^a^** | **Cochran's Q** | **Heterogeneity statistic** | **Degrees of freedom** | **P_Het_** | **I^2^** |
| --- | --- | --- | --- | --- | --- | --- | --- | --- | --- | --- |
| BMI | Depressive symptoms | All | Both | 0.81 (0.66, 0.99) | **0.042** | 27.82 | 1.21 | 19 | 0.087 | 31.70% |
| BMI | Depressive symptoms | Male | Both | 0.53 (0.35, 0.81) | ***0.003*** | 6.20 | 0.83 | 9 | 0.720 | 0.00% |
| BMI | Depressive symptoms | Female | Both | 0.92 (0.73, 1.17) | 0.508 | 16.55 | 1.36 | 9 | 0.056 | 45.60% |
| BMI | Depressive symptoms | All | Urban only | 1.10 (0.76, 1.59) | 0.621 | 11.39 | 1.13 | 9 | 0.250 | 20.90% |
| BMI | Depressive symptoms | Male | Urban only | 0.43 (0.19, 0.95) | **0.037** | 1.99 | 0.71 | 4 | 0.738 | 0.00% |
| BMI | Depressive symptoms | Female | Urban only | 1.42 (0.94, 2.16) | 0.100 | 2.61 | 0.81 | 4 | 0.626 | 0.00% |
| BMI | Depressive symptoms | All | Rural only | 0.70 (0.55, 0.90) | ***0.005*** | 12.54 | 1.18 | 9 | 0.184 | 28.30% |
| BMI | Depressive symptoms | Male | Rural only | 0.57 (0.35, 0.94) | **0.029** | 3.83 | 0.98 | 4 | 0.429 | 0.00% |
| BMI | Depressive symptoms | Female | Rural only | 0.75 (0.56, 1.00) | **0.051** | 7.88 | 1.40 | 4 | 0.096 | 49.30% |
| BMI | Major depression | All | Both | 0.75 (0.49, 1.16) | 0.202 | 27.21 | 1.20 | 19 | 0.100 | 30.20% |
| BMI | Major depression | Male | Both | 0.32 (0.13, 0.79) | ***0.013*** | 11.59 | 1.14 | 9 | 0.238 | 22.30% |
| BMI | Major depression | Female | Both | 0.98 (0.59, 1.60) | 0.920 | 11.09 | 1.11 | 9 | 0.270 | 18.80% |
| BMI | Major depression | All | Urban only | 1.45 (0.69, 3.04) | 0.325 | 8.46 | 0.97 | 9 | 0.488 | 0.00% |
| BMI | Major depression | Male | Urban only | 1.11 (0.22, 5.55) | 0.899 | 6.34 | 1.26 | 4 | 0.175 | 36.90% |
| BMI | Major depression | Female | Urban only | 1.56 (0.68, 3.58) | 0.297 | 1.99 | 0.71 | 4 | 0.738 | 0.00% |
| BMI | Major depression | All | Rural only | 0.53 (0.31, 0.91) | **0.022** | 14.14 | 1.25 | 9 | 0.117 | 36.40% |
| BMI | Major depression | Male | Rural only | 0.18 (0.06, 0.53) | ***0.002*** | 1.83 | 0.68 | 4 | 0.768 | 0.00% |
| BMI | Major depression | Female | Rural only | 0.75 (0.41, 1.40) | 0.369 | 7.21 | 1.34 | 4 | 0.125 | 44.60% |
| WHR | Depressive symptoms | All | Both | 0.74 (0.55, 1.00) | **0.049** | 22.05 | 1.08 | 19 | 0.282 | 13.80% |
| WHR | Depressive symptoms | Male | Both | 0.66 (0.37, 1.18) | 0.162 | 13.51 | 1.23 | 9 | 0.141 | 33.40% |
| WHR | Depressive symptoms | Female | Both | 0.77 (0.54, 1.10) | 0.148 | 8.35 | 0.96 | 9 | 0.499 | 0.00% |
| WHR | Depressive symptoms | All | Urban only | 0.82 (0.48, 1.40) | 0.460 | 8.10 | 0.95 | 9 | 0.524 | 0.00% |
| WHR | Depressive symptoms | Male | Urban only | 0.45 (0.17, 1.16) | 0.096 | 4.05 | 1.01 | 4 | 0.399 | 1.20% |
| WHR | Depressive symptoms | Female | Urban only | 1.09 (0.57, 2.08) | 0.806 | 1.78 | 0.67 | 4 | 0.776 | 0.00% |
| WHR | Depressive symptoms | All | Rural only | 0.71 (0.49, 1.02) | 0.061 | 13.75 | 1.24 | 9 | 0.132 | 34.50% |
| WHR | Depressive symptoms | Male | Rural only | 0.83 (0.40, 1.72) | 0.624 | 8.42 | 1.45 | 4 | 0.077 | 52.50% |
| WHR | Depressive symptoms | Female | Rural only | 0.67 (0.44, 1.02) | 0.059 | 5.05 | 1.12 | 4 | 0.282 | 20.80% |
| WHR | Major depression | All | Both | 0.84 (0.45, 1.57) | 0.580 | 11.51 | 0.78 | 19 | 0.905 | 0.00% |
| WHR | Major depression | Male | Both | 0.58 (0.17, 1.97) | 0.380 | 4.86 | 0.74 | 9 | 0.847 | 0.00% |
| WHR | Major depression | Female | Both | 0.96 (0.46, 1.99) | 0.904 | 6.17 | 0.83 | 9 | 0.722 | 0.00% |
| WHR | Major depression | All | Urban only | 1.40 (0.49, 4.05) | 0.530 | 3.38 | 0.61 | 9 | 0.947 | 0.00% |
| WHR | Major depression | Male | Urban only | 1.09 (0.16, 7.71) | 0.929 | 1.95 | 0.70 | 4 | 0.745 | 0.00% |
| WHR | Major depression | Female | Urban only | 1.56 (0.44, 5.51) | 0.491 | 1.34 | 0.58 | 4 | 0.854 | 0.00% |
| WHR | Major depression | All | Rural only | 0.63 (0.29, 1.38) | 0.250 | 6.72 | 0.86 | 9 | 0.666 | 0.00% |
| WHR | Major depression | Male | Rural only | 0.38 (0.08, 1.85) | 0.229 | 2.23 | 0.75 | 4 | 0.694 | 0.00% |
| WHR | Major depression | Female | Rural only | 0.75 (0.30, 1.83) | 0.522 | 3.96 | 1.00 | 4 | 0.411 | 0.00% |

*P^a^ adjusted for age, regional principal components and chip*

Bold p values reach P<0.05 significance and bold italicised p values reach the multiple testing threshold P<0.013 (see methods)

Table S11. Results of the 2 sample Mendelian Randomisation analysis using the SNPS from the BioBank Japan (Akiyama et al. 2017) for the five meta-analyses from the recent EAS GWAS.

|  | **BMI** |  |  |
| --- | --- | --- | --- |
| **Trait** | **OR IVW (95%CI)** | **P _IVW_** | **N SNPS** |
| Broad discovery analysis | 0.977 (0.947, 1.009) | 0.16 | 74 |
| Clinical depression analysis | 0.962 (0.914, 1.014) | 0.15 | 68 |
| Symptom based analysis | 0.995 (0.948, 1.044) | 0.84 | 65 |
| Resident in East Asia | 0.972 (0.930, 1.016) | 0.22 | 71 |
| Resident in UK or USA | 0.989 (0.947, 1.033) | 0.62 | 74 |

Data are presented as Odds Ratio (95% confidence interval).

IVW = inverse-variant weighted method.

We present the 2-sample MR IVW estimates which assumes that there is no horizontal pleiotropy (under a fixed effect model) or, if implemented under a random effects model after detecting heterogeneity among the causal estimates, that: the strength of the association of the genetic instruments with the risk factor is not correlated with the magnitude of the pleiotropic effects; and the pleiotropic effects have an average value of zero.

Bold p values reach P<0.05 significance and bold italicised p values reach the multiple testing threshold P<0.025 (see methods)

Table S12: The metanalysed non-linear estimates using a control function method

| **Exposure** | **Strata** | **Region** | **N cases (controls)** |  | **beta^2^ (95% CI) per SD higher BMI** | **P^a^** |
| --- | --- | --- | --- | --- | --- | --- |
| Depressive symptoms | All | Both | 3,398 (96,979) |  | 0.010 (-0.003, 0.022) | 0.121 |
|  | Male | Both | 1,170 (41,764) |  | 0.008 (-0.013, 0.028) | 0.471 |
|  | Female | Both | 2,228 (55,215) |  | 0.011 (-0.004, 0.026) | 0.164 |
|  | All | Urban only | 1,080 (42,800) |  | 0.024 (0.003, 0.044) | **0.023** |
|  | Male | Urban only | 326 (17,639) |  | 0.011 (-0.026, 0.048) | 0.569 |
|  | Female | Urban only | 754 (25,161) |  | 0.029 (0.005, 0.054) | **0.019** |
|  | All | Rural only | 2,318 (54,179) |  | 0.002 (-0.013, 0.017) | 0.798 |
|  | Male | Rural only | 844 (24,125) |  | 0.006 (-0.019, 0.031) | 0.626 |
|  | Female | Rural only | 1,474 (30,054) |  | 0.000 (-0.019, 0.018) | 0.960 |

*P^a^ adjusted for age, regional principal components and chip*

Bold p values reach P<0.05 significance and bold italicised p values reach the multiple testing threshold P<0.013 (see methods)

Table S13: The observational and genetic 1-sample associations between BMI/WHR and major depression in individuals with valid genetic data from the China Kadoorie Biobank.

|  |  |  |  |  | **Observational** | |  | **Genetic** | | | |
| --- | --- | --- | --- | --- | --- | --- | --- | --- | --- | --- | --- |
| **Instrument used** | **Strata** | **Region** | **N cases (controls)** |  | **OR (95% CI) per SD higher BMI** | **P^a^** |  | **OR (95% CI) per SD higher BMI** | **P^b^** | **P_sex_** | **P_region_** |
| BMI | All | Both | 760 (96,979) |  | 0.83 (0.78, 0.90) | 1.50E-06 |  | 0.75 (0.49, 1.16) | 0.202 |  |  |
|  | Male | Both | 246 (41,764) |  | 0.76 (0.66, 0.87) | 4.60E-05 |  | 0.32 (0.13, 0.79) | ***0.013*** | 0.033 |  |
|  | Female | Both | 514 (55,215) |  | 0.86 (0.79, 0.94) | 1.20E-03 |  | 0.98 (0.59, 1.60) | 0.920 |  |  |
|  | All | Urban only | 281 (42,800) |  | 0.87 (0.77, 0.98) | 0.027 |  | 1.45 (0.69, 3.04) | 0.325 |  | 0.032 |
|  | Male | Urban only | 79 (17,639) |  | 0.96 (0.76, 1.22) | 0.760 |  | 1.11 (0.22, 5.55) | 0.899 | 0.714 | 0.064 |
|  | Female | Urban only | 202 (25,161) |  | 0.83 (0.72, 0.96) | 0.012 |  | 1.56 (0.68, 3.58) | 0.297 |  | 0.170 |
|  | All | Rural only | 479 (54,179) |  | 0.82 (0.75, 0.90) | 4.40E-05 |  | 0.53 (0.31, 0.91) | **0.022** |  |  |
|  | Male | Rural only | 167 (24,125) |  | 0.67 (0.57, 0.80) | 3.80E-06 |  | 0.18 (0.06, 0.53) | **2.00E-03** | 0.023 |  |
|  | Female | Rural only | 312 (30,054) |  | 0.89 (0.80, 1.00) | 0.053 |  | 0.75 (0.41, 1.40) | 0.369 |  |  |
| WHR | All | Both | 760 (96,979) |  | 0.89 (0.83, 0.96) | 2.30E-03 |  | 0.84 (0.45, 1.57) | 0.580 |  |  |
|  | Male | Both | 246 (41,764) |  | 0.83 (0.73, 0.94) | 3.00E-03 |  | 0.58 (0.17, 1.97) | 0.380 | 0.488 |  |
|  | Female | Both | 514 (55,215) |  | 0.92 (0.84, 1.00) | 0.059 |  | 0.96 (0.46, 1.99) | 0.904 |  |  |
|  | All | Urban only | 281 (42,800) |  | 0.89 (0.78, 1.01) | 0.075 |  | 1.40 (0.49, 4.05) | 0.530 |  | 0.234 |
|  | Male | Urban only | 79 (17,639) |  | 0.98 (0.78, 1.24) | 0.870 |  | 1.09 (0.16, 7.71) | 0.929 | 0.765 | 0.409 |
|  | Female | Urban only | 202 (25,161) |  | 0.85 (0.73, 0.99) | 0.031 |  | 1.56 (0.44, 5.51) | 0.491 |  | 0.351 |
|  | All | Rural only | 479 (54,179) |  | 0.91 (0.83, 1.00) | 0.058 |  | 0.63 (0.29, 1.38) | 0.250 |  |  |
|  | Male | Rural only | 167 (24,125) |  | 0.77 (0.66, 0.90) | 1.10E-03 |  | 0.38 (0.08, 1.85) | 0.229 | 0.467 |  |
|  | Female | Rural only | 312 (30,054) |  | 0.99 (0.88, 1.11) | 0.820 |  | 0.75 (0.30, 1.83) | 0.522 |  |  |

Pa adjusted for age, region and sex

Pb adjusted for age, regional principal components and chip

Psex comparison between male and female estimates using Fisher’s z-score method.

Pregion comparison between urban and rural estimates using Fisher’s z-score method.

Underlined p values represent a significant (P<0.05) difference when comparing the by sex or by region estimates.

Bold p values reach P<0.05 significance and bold italicised p values reach the multiple testing threshold P<0.013 (see methods)

Table S14: The genetic 1-sample associations between BMI/WHR and depressive symptoms in individuals with valid genetic data from the China Kadoorie Biobank removing Qingdao with a <1% prevalence of depressive symptoms.

| **Instrument used** | **Strata** | **Region** | **N cases (controls)** |  | **OR (95% CI) per SD higher adiposity measure** | **P^a^** | **P_sex_** | **P_region_** |
| --- | --- | --- | --- | --- | --- | --- | --- | --- |
| BMI | All | Both | 3,340 (88,778) |  | 0.80 (0.65, 0.99) | **0.042** |  |  |
|  | Male | Both | 1,152 (38,496) |  | 0.53 (0.34, 0.81) | ***3.00E-03*** | 0.025 |  |
|  | Female | Both | 2,188 (50,282) |  | 0.92 (0.72, 1.17) | 0.498 |  |  |
|  | All | Urban only | 1,032 (34,599) |  | 1.11 (0.76, 1.62) | 0.604 |  | 0.049 |
|  | Male | Urban only | 308 (14,371) |  | 0.41 (0.18, 0.94) | **0.036** | 8.77E-03 | 0.499 |
|  | Female | Urban only | 714 (20,228) |  | 1.43 (0.94, 2.20) | 0.098 |  | 0.014 |
|  | All | Rural only | 2,318 (54,179) |  | 0.70 (0.55, 0.90) | ***0.005*** |  |  |
|  | Male | Rural only | 844 (24,125) |  | 0.57 (0.35, 0.94) | **0.029** | 0.363 |  |
|  | Female | Rural only | 1,474 (30,054) |  | 0.75 (0.56, 1.00) | **0.051** |  |  |
| WHR | All | Both | 3,340 (88,778) |  | 0.73 (0.54, 1.00) | **0.047** |  |  |
|  | Male | Both | 1,152 (38,496) |  | 0.65 (0.37, 1.16) | 0.145 | 0.634 |  |
|  | Female | Both | 2,188 (50,282) |  | 0.77 (0.54, 1.10) | 0.151 |  |  |
|  | All | Urban only | 1,032 (34,599) |  | 0.80 (0.46, 1.40) | 0.442 |  | 0.699 |
|  | Male | Urban only | 308 (14,371) |  | 0.42 (0.16, 1.10) | 0.078 | 0.105 | 0.267 |
|  | Female | Urban only | 714 (20,228) |  | 1.12 (0.56, 2.21) | 0.752 |  | 0.206 |
|  | All | Rural only | 2,318 (54,179) |  | 0.71 (0.49, 1.02) | 0.061 |  |  |
|  | Male | Rural only | 844 (24,125) |  | 0.83 (0.40, 1.72) | 0.624 | 0.597 |  |
|  | Female | Rural only | 1,474 (30,054) |  | 0.67 (0.44, 1.02) | 0.059 |  |  |

P^a^ adjusted for age, regional principal components and chip

Psex comparison between male and female estimates using Fisher’s z-score method.

Pregion comparison between urban and rural estimates using Fisher’s z-score method.

Underlined p values represent a significant (P<0.05) difference when comparing the by sex or by region estimates.

Bold p values reach P<0.05 significance and bold italicised p values reach the multiple testing threshold P<0.0
