# Supplementary material for: Mendelian randomisation study of body composition and depression in people of East Asian ancestry highlights potential setting-specific causality
Source: BMC Med. 2023 Feb 1;21:37. doi: 10.1186/s12916-023-02735-8 (PMC9893684; doi:10.1186/s12916-023-02735-8)

Fig. S1: Forest plot of the observational and genetic associations between a 1-SD higher WHR and the odds of depressive symptoms. The plot displays the observational association and the genetic association using the two-step instrumental variable analysis with the WHR genetic risk score (Genetic 1-sample).

Fig. S2. The genetic 1-sample Mendelian Randomisation estimates of BMI to depressive symptoms stratified by sex and region.

Fig. S3. The genetic 1-sample Mendelian Randomisation estimates of BMI to major depression stratified by sex and region.

Fig. S4. Scatter plots of SNP-“Broad discovery” vs. SNP-BMI 2-sample MR analyses. Left is the main analysis as presented in Table 1, centre and right are the sensitivity analyses excluding SNPs as presented in Additional file 2: Tables S3-S4, respectively.


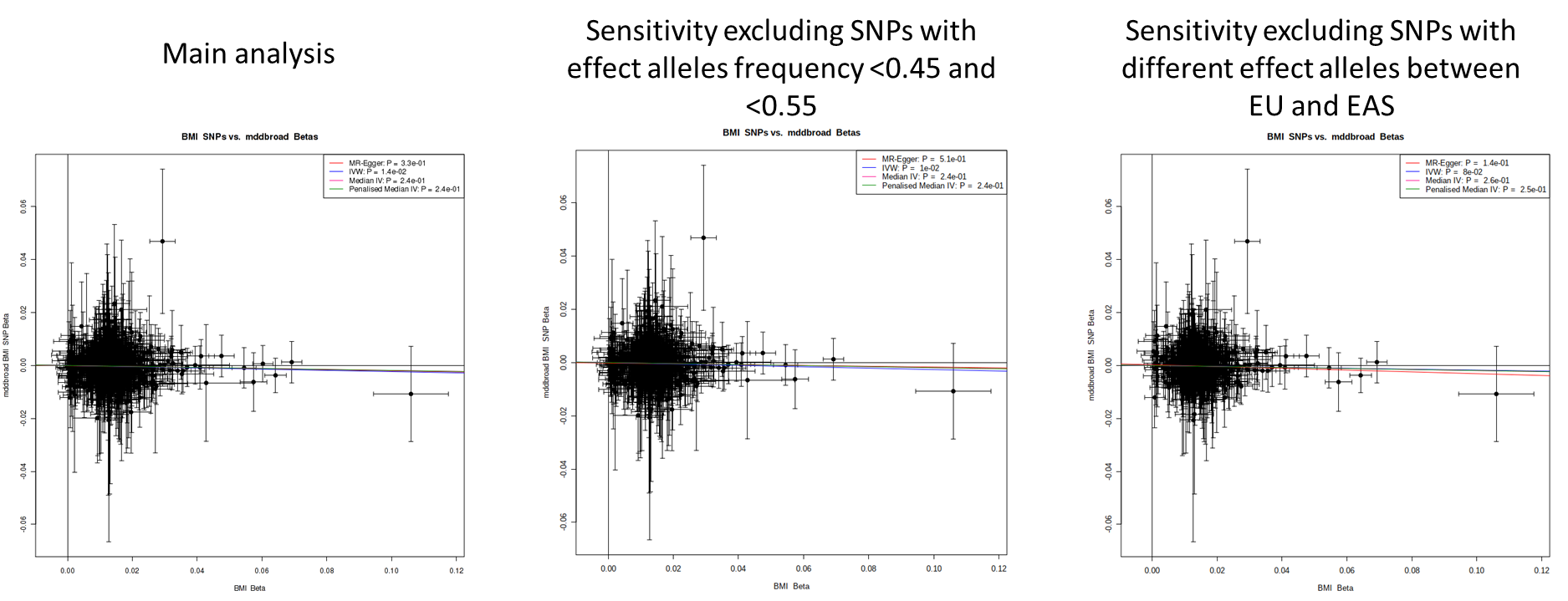


Fig. S5. Scatter plots of SNP-“Clinical depression” vs. SNP-BMI 2-sample MR analyses. Left is the main analysis as presented in Table 1, centre and right are the sensitivity analyses excluding SNPs as presented in Additional file 2: Tables S3-S4, respectively.


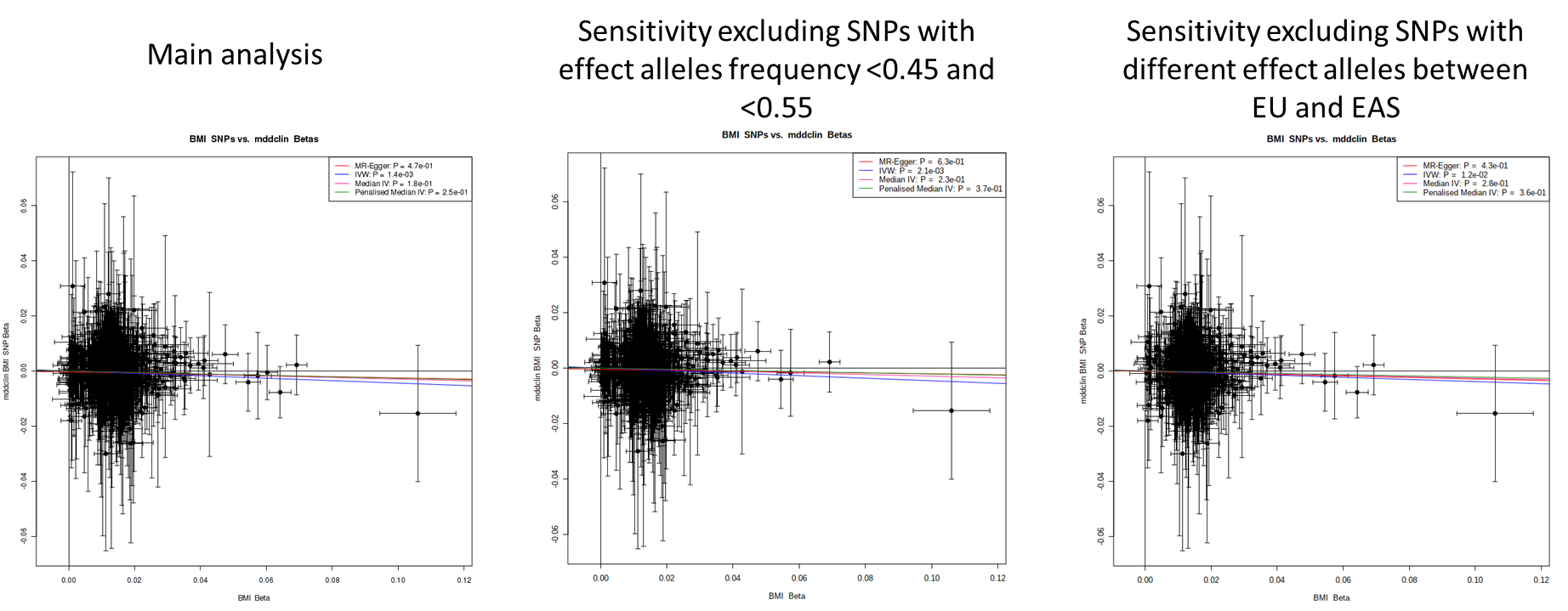


Fig. S6. Scatter plots of SNP-“Symptom-based analyses” vs. SNP-BMI 2-sample MR analyses. Left is the main analysis as presented in Table 1, centre and right are the sensitivity analyses excluding SNPs as presented in Additional file 2: Tables S3-S4, respectively.


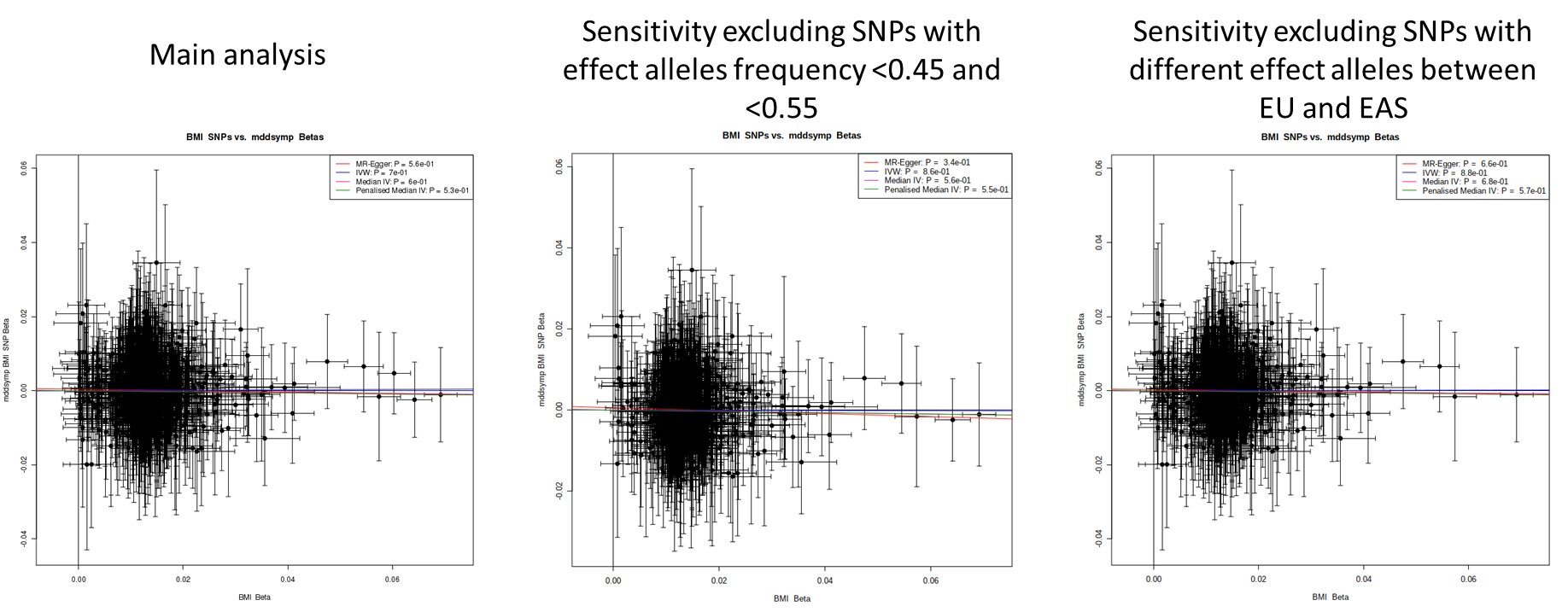


Fig. S7. Scatter plots of SNP-“EAS individuals living in East Asia” vs. SNP-BMI 2-sample MR analyses. Left is the main analysis as presented in Table 1, centre and right are the sensitivity analyses excluding SNPs as presented in Additional file 2: Tables S3-S4, respectively.


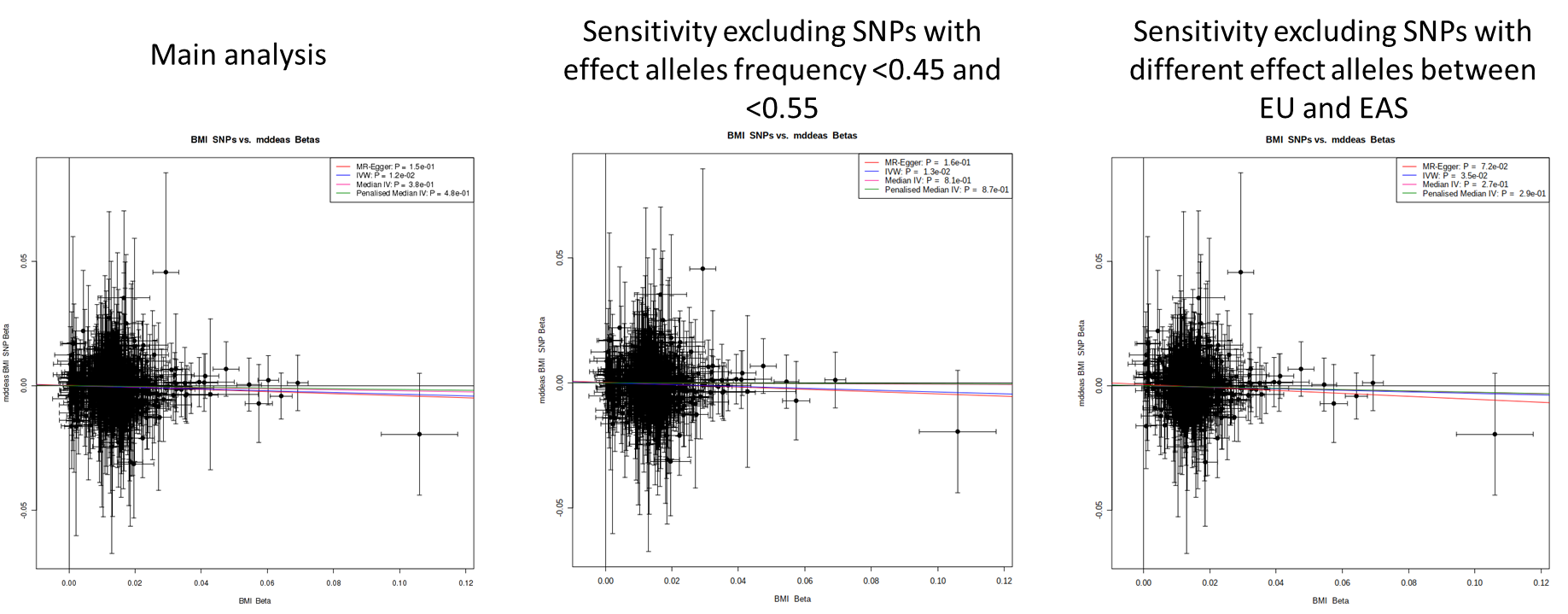


Fig. S8. Scatter plots of SNP-“EAS individuals living in Western countries (UK and USA)” vs. SNP-BMI 2-sample MR analyses. Left is the main analysis as presented in Table 1, centre and right are the sensitivity analyses excluding SNPs as presented in Additional file 2: Tables S3-S4, respectively.


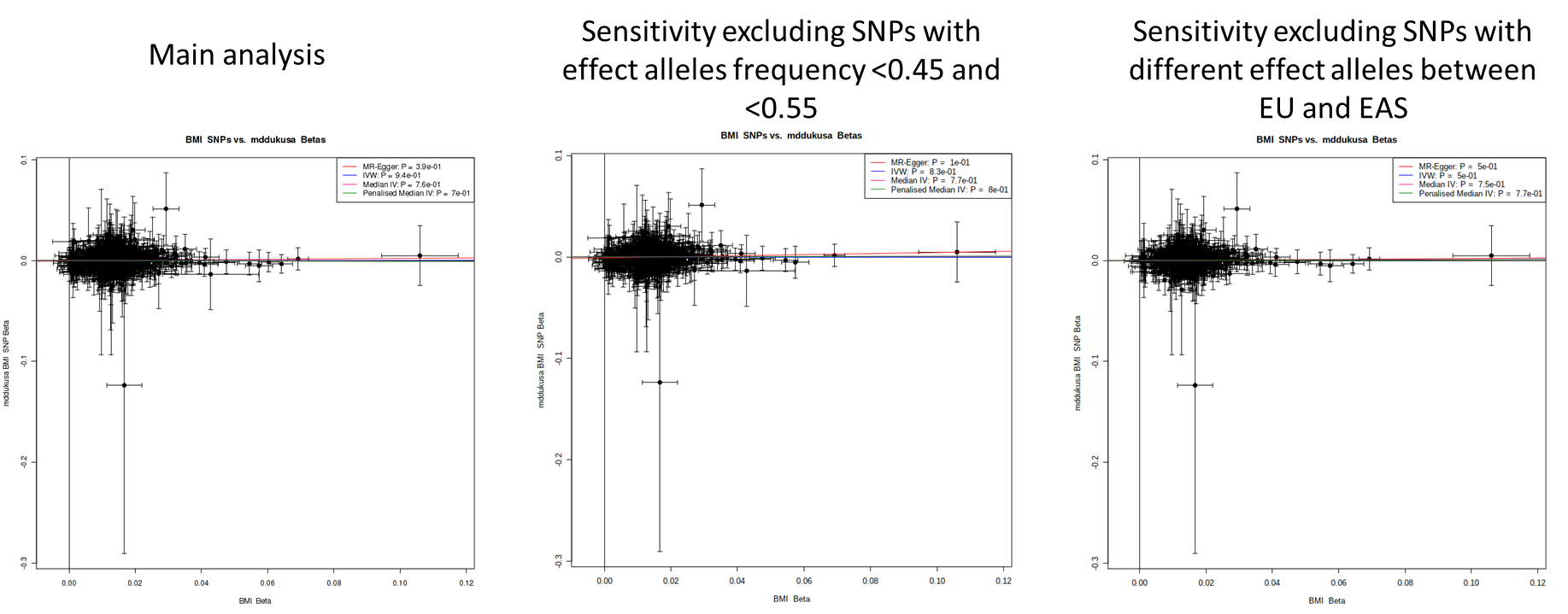


Fig. S9. Scatter plots of SNP-“Broad discovery” vs. SNP-WHR 2-sample MR analyses. Left is the main analysis as presented in Table 1, centre and right are the sensitivity analyses excluding SNPs as presented in Additional file 2: Tables S3-S4, respectively.


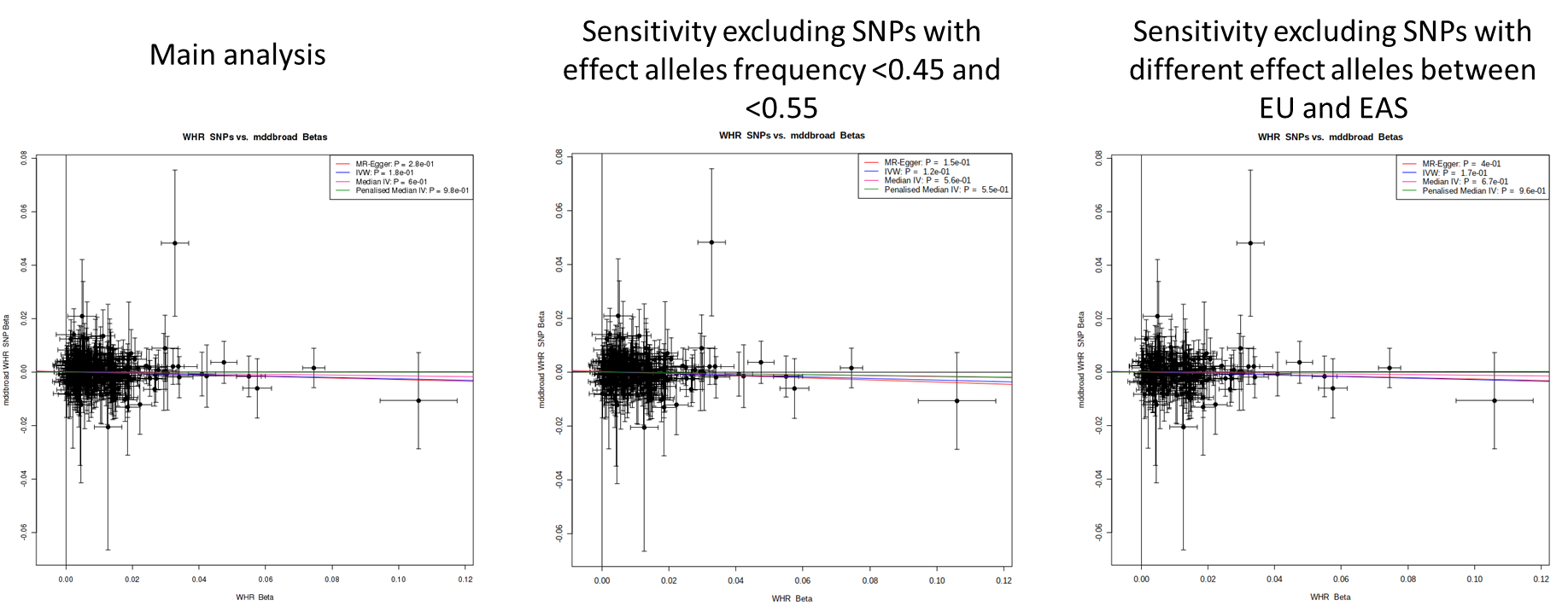


Fig. S10. Scatter plots of SNP-“Clinical depression” vs. SNP-WHR 2-sample MR analyses. Left is the main analysis as presented in Table 1, centre and right are the sensitivity analyses excluding SNPs as presented in Additional file 2: Tables S3-S4, respectively.


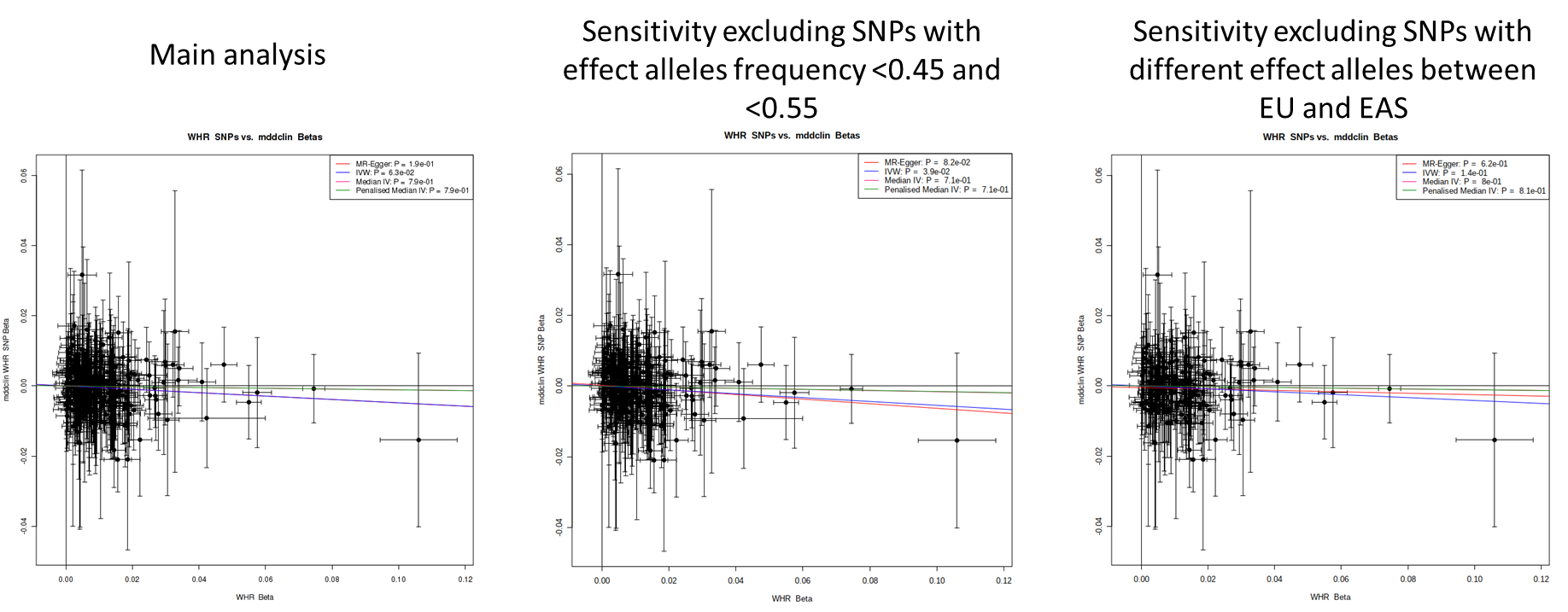


Fig. S11. Scatter plots of SNP-“Symptom-based analyses” vs. SNP-WHR 2-sample MR analyses. Left is the main analysis as presented in Table 1, centre and right are the sensitivity analyses excluding SNPs as presented in Additional file 2: Tables S3-S4, respectively.


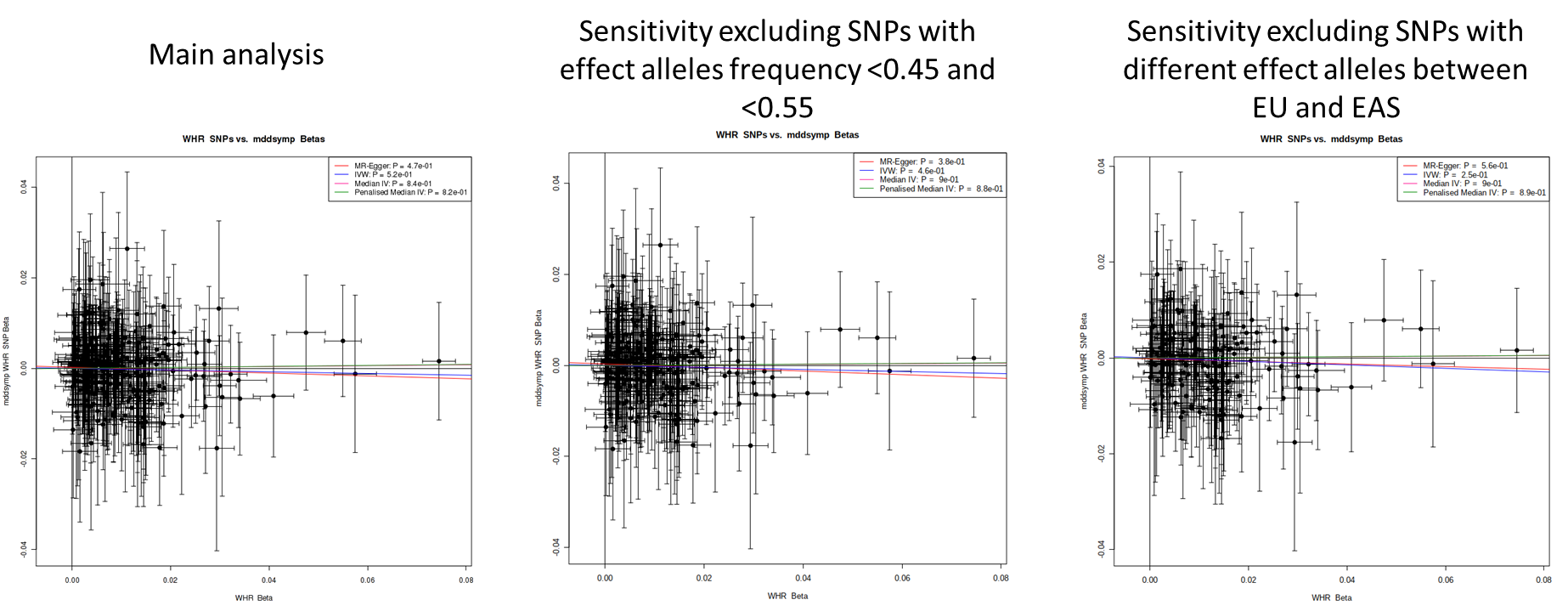


Fig. S12. Scatter plots of SNP-“EAS individuals living in East Asia” vs. SNP-WHR 2-sample MR analyses. Left is the main analysis as presented in Table 1, centre and right are the sensitivity analyses excluding SNPs as presented in Additional file 2: Tables S3-S4, respectively.


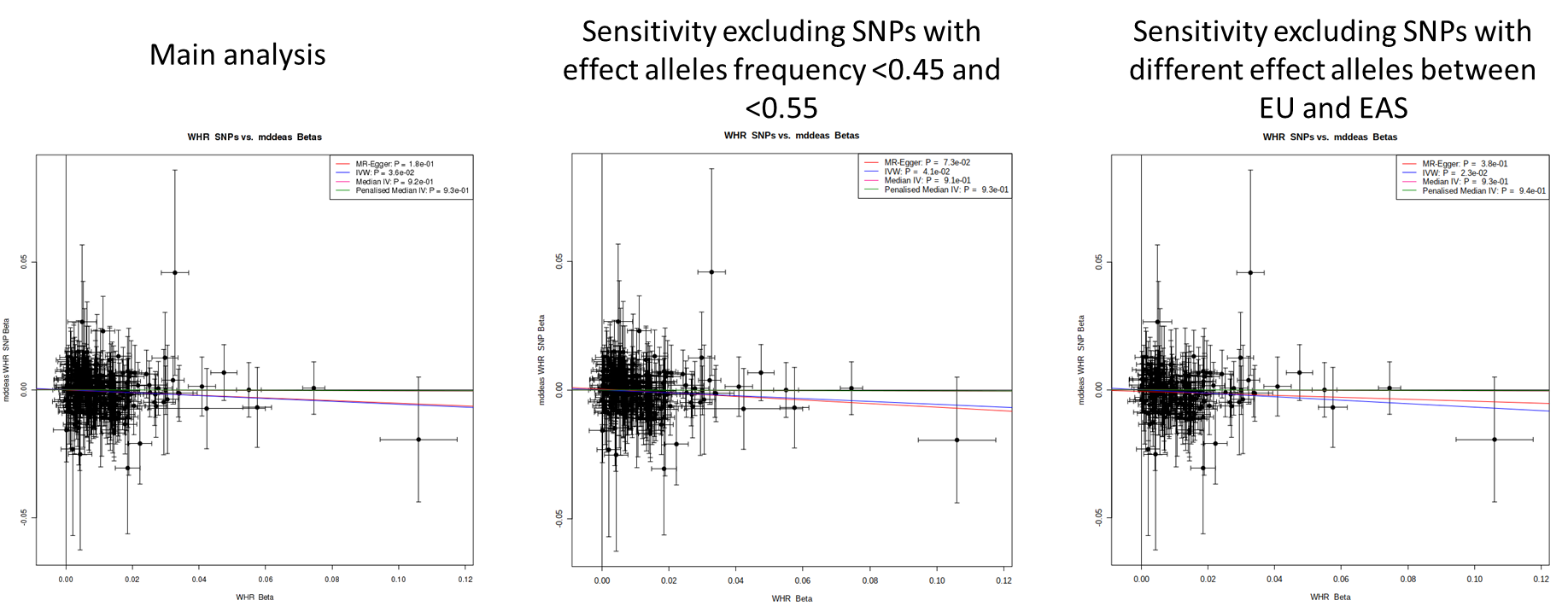


Fig. S13. Scatter plots of SNP-“EAS individuals living in Western countries (UK and USA)” vs. SNP-WHR 2-sample MR analyses. Left is the main analysis as presented in Table 1, centre and right are the sensitivity analyses excluding SNPs as presented in Additional file 2: Tables S3-S4, respectively.


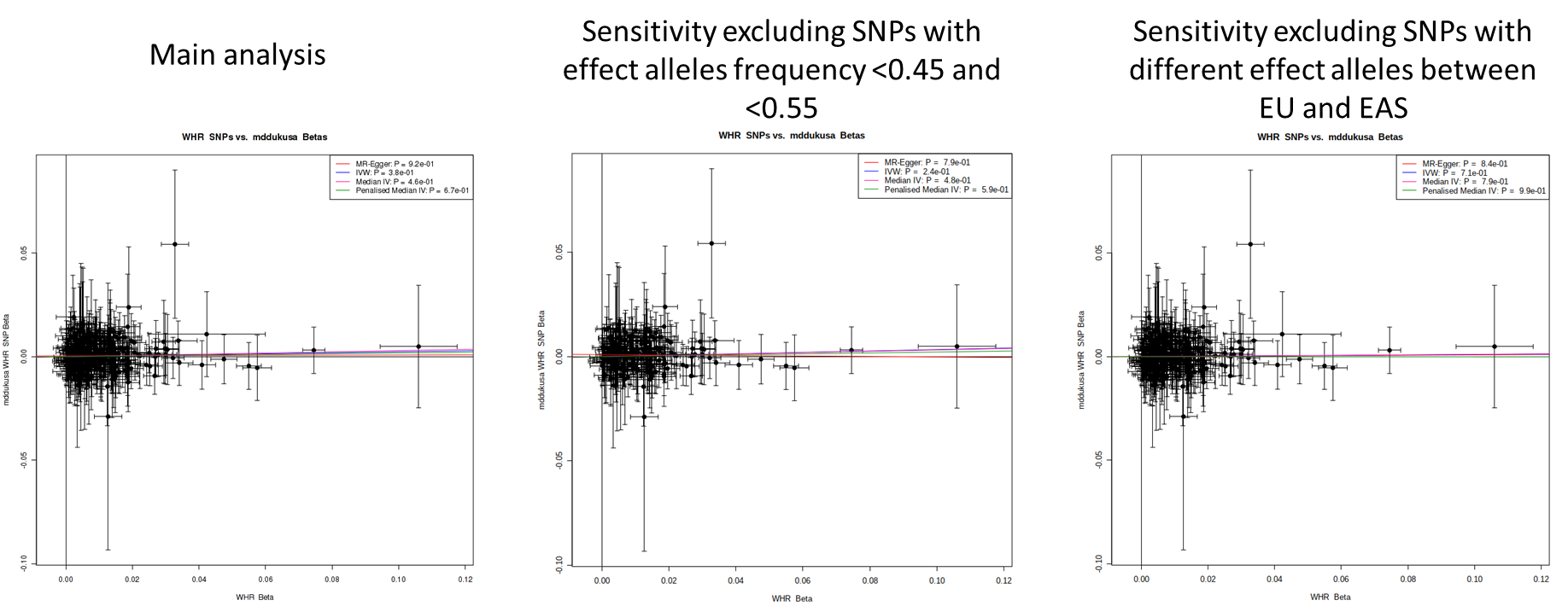

Supplement: Supplementary file 3 — Additional file 3: Fig. S1. Observational and genetic associationsbetween WHR and depressive symptoms. Fig. S2. The genetic 1-sample MR estimatesof BMI to depressive symptoms stratified by sex and region. Fig. S3. Thegenetic 1-sample MR estimates of BMI to major depression stratified by sex andregion. Figs. S4-S8. Scatter plots of SNP-depression outcome vs. SNP-BMI2-sample MR analyses. Figs. S9-S13. Scatter plots of SNP-depression outcome vs.SNP-WHR 2-sample MR analyses. [file 12916_2023_2735_MOESM3_ESM.docx]
